# Synthetic gene circuits for cell state detection and protein tuning in human pluripotent stem cells

**DOI:** 10.15252/msb.202110886

**Published:** 2022-11-11

**Authors:** Laura Prochazka, Yale S Michaels, Charles Lau, Ross D Jones, Mona Siu, Ting Yin, Diana Wu, Esther Jang, Mercedes Vázquez‐Cantú, Penney M Gilbert, Himanshu Kaul, Yaakov Benenson, Peter W Zandstra

**Affiliations:** ^1^ Institute of Biomedical Engineering (BME) University of Toronto Toronto ON Canada; ^2^ Donnelly Centre for Cellular & Biomolecular Research University of Toronto Toronto ON Canada; ^3^ Michael Smith Laboratories University of British Columbia Vancouver BC Canada; ^4^ School of Biomedical Engineering University of British Columbia Vancouver BC Canada; ^5^ Swiss Federal Institute of Technology (ETH) Zürich, Department of Biosystems Science and Engineering (D‐BSSE) Basel Switzerland; ^6^ Department of Cell and Systems Biology University of Toronto Toronto ON Canada; ^7^ School of Engineering University of Leicester Leicester UK; ^8^ Department of Respiratory Sciences University of Leicester Leicester UK

**Keywords:** cell fate control, classification, human pluripotent stem cells, protein tuning, synthetic gene circuits, Biotechnology & Synthetic Biology, Stem Cells & Regenerative Medicine

## Abstract

During development, cell state transitions are coordinated through changes in the identity of molecular regulators in a cell type‐ and dose‐specific manner. The ability to rationally engineer such transitions in human pluripotent stem cells (hPSC) will enable numerous applications in regenerative medicine. Herein, we report the generation of synthetic gene circuits that can detect a desired cell state using AND‐like logic integration of endogenous miRNAs (classifiers) and, upon detection, produce fine‐tuned levels of output proteins using an miRNA‐mediated output fine‐tuning technology (miSFITs). Specifically, we created an “hPSC ON” circuit using a model‐guided miRNA selection and circuit optimization approach. The circuit demonstrates robust PSC‐specific detection and graded output protein production. Next, we used an empirical approach to create an “hPSC‐Off” circuit. This circuit was applied to regulate the secretion of endogenous BMP4 in a state‐specific and fine‐tuned manner to control the composition of differentiating hPSCs. Our work provides a platform for customized cell state‐specific control of desired physiological factors in hPSC, laying the foundation for programming cell compositions in hPSC‐derived tissues and beyond.

## Graphical abstract

Synthetic gene circuits with sense‐and‐respond capabilities were designed to detect cell states and perform cell state‐specific control of desired protein expression in human pluripotent stem cells (hPSCs).

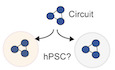

## Introduction

Robust gene‐regulatory programs enable stem cells to self‐renew and differentiate by sensing and responding to stimuli in a defined manner. Crucially, these regulatory circuits are capable of integrating multiple internal and external input signals to achieve a high degree of specificity, resulting in lineage or cell‐state‐specific activation of effector molecules (Arnold & Robertson, 2009; Ruiz‐Herguido *et al*, 2012). The production of effector molecules is often graded, where defined doses can lead to desirable proportions of downstream lineages (Zhang *et al*, 1998; Müller *et al*, 2012; Manfrin *et al*, 2019). The ability to engineer such gene‐regulatory circuits into human pluripotent stem cells (hPSC) *de novo* would enable efficient production of desired cell types or tissues for research and regenerative medicine applications (Galloway *et al*, 2013; Lipsitz *et al*, 2016; Teague *et al*, 2016; Prochazka *et al*, 2017; Santorelli *et al*, 2019).

With the goal to control human cell function, substantial effort has been directed toward synthetic gene circuit engineering in human cells (Tigges *et al*, 2009; Greber & Fussenegger, 2010; Wei *et al*, 2013; Duportet *et al*, 2014; Kiani *et al*, 2014; Morsut *et al*, 2016; Weinberg *et al*, 2017; Ausländer *et al*, 2018; Szenk *et al*, 2020), with recent exciting developments using hPSC (Lienert *et al*, 2013; Guye *et al*, 2016; Saxena *et al*, 2016; Gao *et al*, 2018; Velazquez *et al*, 2021). The majority of gene circuits implemented in human cells are logic gene circuits (Bronson *et al*, 2007; Rinaudo *et al*, 2007; Leisner *et al*, 2010; Auslände *et al*, 2012; Lohmueller *et al*, 2012; Cho *et al*, 2021). A handful of these circuits have been designed to detect cell‐internal endogenous input signals, enabling restriction of circuit action to desired cell types or cell states (Xie *et al*, 2011; Baertsch *et al*, 2014; Prochazka *et al*, 2014; Miki *et al*, 2015; Angelici *et al*, 2016; Doshi *et al*, 2020). Here we define a cell state as discrete if it can be clearly discriminated from other cell states on the basis of a predefined set of molecular inputs that are detected and integrated by a circuit. The underlying circuit integrates the inputs in a function that can be approximated by Boolean logic and autonomously “decides” if a desired downstream molecule, the output, is produced at high (On) or low (Off) concentrations. One type of circuit that allows such discrete cell state discrimination is cell “classifiers” (Xie *et al*, 2011; Mohammadi *et al*, 2017). Cell classifiers have been designed to detect and logically integrate endogenous microRNAs (miRNAs) and have proven useful for a variety of applications such as the specific killing of cancer cells (Xie *et al*, 2011; Dastor *et al*, 2018) or for screening miRNA drug candidates (Haefliger *et al*, 2016). Additionally, single endogenous miRNAs have been employed to regulate synthetic genes to discriminate hPSCs from differentiated cells (Brown *et al*, 2007), for selection of PSC‐derived mature cell types (Miki *et al*, 2015) or reprogrammed induced hPSC (di Stefano *et al*, 2011), and for specific killing of hPSC (Miki *et al*, 2015; Parr *et al*, 2016; Fujita *et al*, 2022). Interestingly, endogenous miRNAs have also been exploited to fine‐tune expression levels of synthetic and natural genes in human cells (Michaels *et al*, 2019). Such graded production of proteins is crucial for many applications where precise intervention of physiological behavior is required (Michaels *et al*, 2019).

Despite this progress, current implementations of cell classifiers result in arbitrary On and Off levels that are highly dependent on parameters such as the promoter strength and delivery system and thus are difficult to tune to the desired dose (Xie *et al*, 2011; Lapique & Benenson, 2014; Schreiber *et al*, 2016; Prochazka *et al*, 2017). Furthermore, miRNA‐based systems implemented in stem cells typically operate with a single miRNA input and a single protein output (Brown *et al*, 2007; di Stefano *et al*, 2011; Parr *et al*, 2016; Fujita *et al*, 2022), limiting their applications. To date, no circuit has been reported that allows precise tuning of multiple desired proteins from desired discrete cell states, a function that stem cells perform continuously during development and would enable powerful control over stem cell differentiation.

Here we design and implement synthetic gene circuits that are capable of performing cell‐state‐specific control of desired protein expression in hPSC. Specifically, we provide a platform for engineering gene circuits using transient transfections. The platform combines miRNA‐based logic gate computations (Xie *et al*, 2011) for cell state detection, with miRNA silencing‐mediated fine‐tuners (miSFITs; Michaels *et al*, 2019) to enable precise tuning of the output dose by pre‐selecting desired miSFITs targeted output constructs. We first outline the creation of a generic hPSC‐specific circuit (hPSC‐On circuit) using an automated miRNA identification and circuit design tool and a model‐guided combinatorial screening approach. We next highlight an empirical approach for design and implementation of a minimal circuit that is silenced in hPSC (hPSC‐Off circuit) and utilize this system for the autonomous induction of BMP4 dose‐mediated hPSC microtissue patterning to achieve control over the proportions of differentiated cell types. Our platform lays the foundation for rapid, model‐guided or empirical engineering of cell‐state‐specific circuits that have the ability to tune the output dose to desired levels in hPSC and their derivatives.

### Circuit design

**Figure 1:**
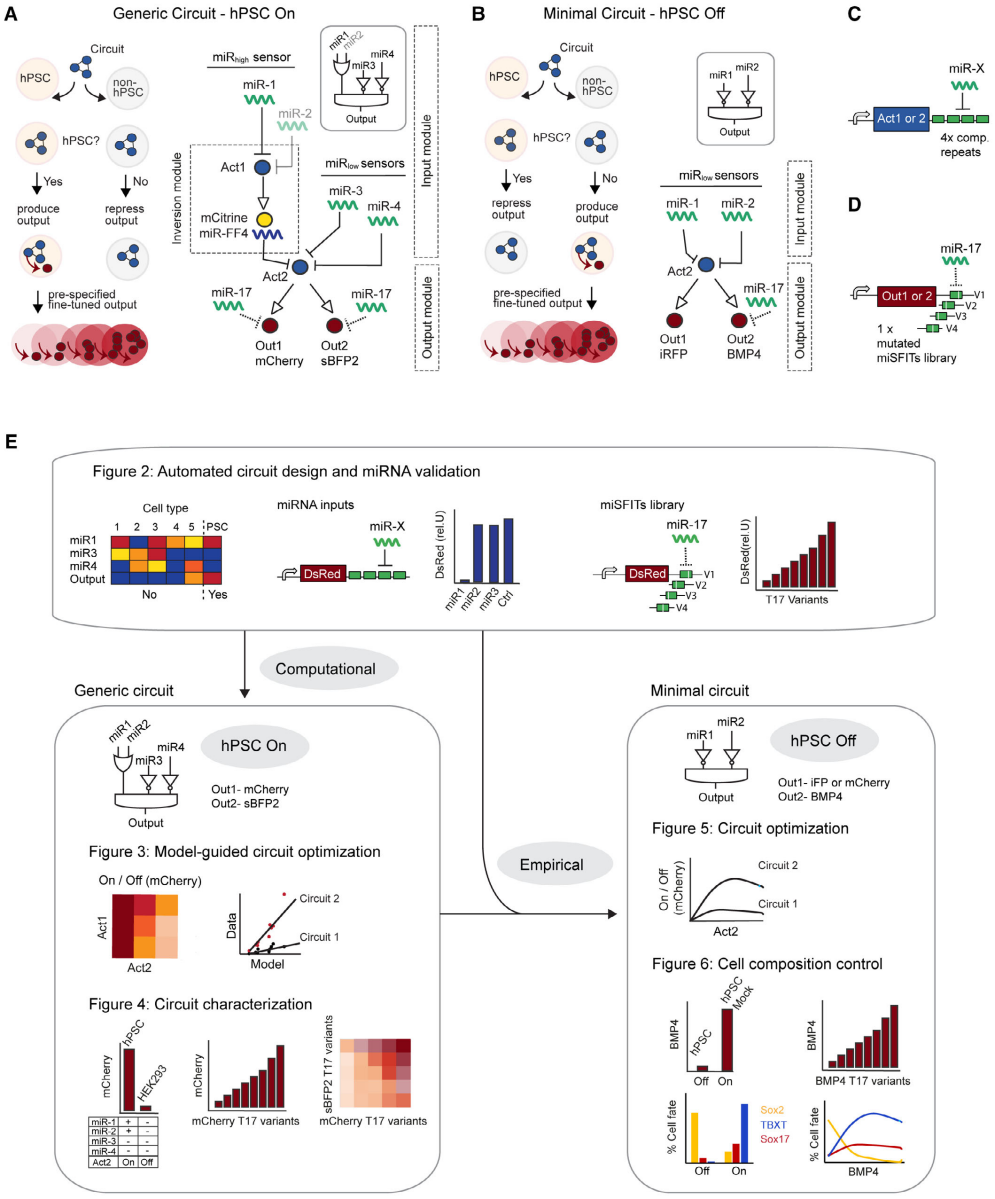
Circuit design A, BSchematic of generic circuit (A) and minimal circuit (B) with cellular performance (left) and circuit architecture (right). Logic input module (top) and output module (bottom). Act1 and Act2 are two different orthogonal synthetic transactivators, miR‐FF4 is a synthetic miRNA‐based repressor. Out is the output protein as indicated. (A) The generic circuit consists of one miR_high_ sensor (inversion module) that recognizes up to two miRNAs (miR1, miR2 = OR gate) and two miR_low_ sensors each recognizing one miRNA (miR3, miR4). The circuit performance can be approximated with the Boolean logic function Output = miR1 OR miR2 AND NOT miR3 AND NOT miR4 and is designed to be active in hPSC (hPSC On). (B) The minimal circuit shows two miR_low_ sensors detecting miR1 and miR2 performing the logic function Output = NOT miR1 AND NOT miR2, designed to be inactive in hPSC (hPSC Off). In both circuits, the output module is controlled by Act2 and shows two protein outputs that are further controlled by miSFITs, an miR‐17‐based target library.CSchematic of the miRNA‐based targeting approach used to create the input module to detect endogenous miRNA inputs in a discrete manner.DSchematic of the output fine‐tuning using miSFITs.EFlowchart and figure guidance for generic and minimal circuit development.

To establish our platform, we designed (i) a generic circuit that detects the pluripotent state, restricting circuit actuation to undifferentiated hPSCs (hPSC‐On circuit; Fig 1A), and (ii) a minimal circuit that represses output production in hPSC (hPSC‐Off circuit; Fig 1B). Our design uses a bow‐tie architecture (Prochazka *et al*, 2014) that allows decomposition of the circuit into two modules: (i) a logic multi‐input module that detects discrete cell states by recognizing a set of miRNAs (Xie *et al*, 2011) and (ii) a multi‐output module that uses a library of miRNA mediated fine‐tuners (miSFITs; Michaels *et al*, 2019) to independently tune the levels of multiple outputs to desired levels.

The generic circuit contains an input module composed of two kinds of miRNA sensors, here named miR_high_ and miR_low_ sensors (Fig 1A). miR_low_ sensors directly repress a synthetic transactivator, Act2, by placing four fully complementary repeats of a given miRNA in the 3′UTR of Act2, resulting in Act2 expression only when the given miRNA is absent or at low levels (Fig 1A and C). miR_high_ sensors are double inversion modules, where the endogenous miRNA represses a transactivator Act1 through four fully complementary repeats in the 3′UTR of Act1 (Fig 1A and C). Act1 in turn induces a repressor, a synthetic miRNA termed miR‐FF4, that does not exist in human cells (Xie *et al*, 2011; Schreiber *et al*, 2016). miR‐FF4 in turn represses Act2 (Fig 1A). Following this cascade, Act2 expression is induced only if a given endogenous miRNA input is highly expressed. An miR_high_ sensor can be targeted by two or more miRNAs, forming an OR gate, to improve the inversion performance and increase robustness to fluctuations in endogenous miRNAs (Xie *et al*, 2011; Schreiber *et al*, 2016; Fig 1A). Because all sensors converge on Act2, the integration of the miRNA signals can be approximated in an AND‐like logic function (Prochazka *et al*, 2014) Output = miR1 OR miR2 AND NOT(miR3) AND NOT(miR4). This means that Act2 is produced at high levels only if highly expressed miRNAs are recognized by the miR_high_ sensors AND if miRNAs that are not expressed or active in pluripotent state are recognized by miR_low_ sensors. If one of the miRNA inputs substantially differs to this profile, or multiple miRNAs differ slightly, Act2‐ and output expression is significantly repressed (Xie *et al*, 2011; Prochazka *et al*, 2014), thereby enabling discrete cell state detection. The minimal circuit, in contrast, operates only on miR_low_ sensors and has been built by targeting Act2 by four complementary repeats of two miRNAs that are highly expressed in hPSC, thereby repressing Act2 and with that the output expression (hPSC‐Off circuit; Fig 1B and C). The minimal circuit performs the AND‐like logic function Output = NOT(miR1) AND NOT(miR2).

In both generic and minimal circuit, the output module is controlled by Act2 and thus, indirectly, by the endogenous miRNAs. Act2 can activate one or multiple protein outputs (Fig 1A and B). To fine‐tune the expression levels of each output, we applied miSFITs, a previously reported approach that operates on a library of mutated target sites of miRNA‐17 (Michaels *et al*, 2019). miR‐17 is ubiquitously and strongly expressed among most human cell types, including hPSC (Data ref: Fogel *et al*, 2015a). To tune protein outputs, one repeat of an miRNA‐17 target site variant was placed in the 3′UTR of the protein outputs (Fig 1D). Each variant contains different mutations in the target site leading to reduced binding strength of endogenous miR‐17 and thus reduced repression. The decrease in repression strength depends on the position and identity of the mismatched nucleotides (Michaels *et al*, 2019). Thus, by selecting a desired mutant variant from the miSFITs library, expression of the output proteins can be tuned to desired levels (Michaels *et al*, 2019).

The generic circuit has been rationally designed where the number and combination of miR_high_ and miR_low_ sensors have been computationally predicted from miRNA expression data of hPSC and hPSC‐derived cell states (Fig 1E, left). The design of the minimal circuit, in contrast, was empirical based on miRNAs and learnings from the generic circuit implementation (Fig 1E, right).

## Results

### Automated circuit design and miRNA validation

In order to restrict circuit action to discrete cell states or cell types, a set of endogenous miRNAs that can clearly discriminate the cell state of interest (positive samples, here hPSC) from the other cell states (negative samples, here hPSC‐derived differentiated cells) require to be identified. To address the challenge of manually selecting such a set of miRNAs, we have applied and further modified a previously developed computational platform that automates the miRNA candidate search and circuit design procedure (Mohammadi *et al*, 2017). This platform uses a mechanistic mathematical model that predicts circuit output production from miRNA expression data by seeking a set of miRNA inputs and underlying circuit with the largest classification margin (cMargin). In other words, the largest fold change in calculated circuit output levels between positive samples and negative samples (Mohammadi *et al*, 2017).

**Figure 2:**
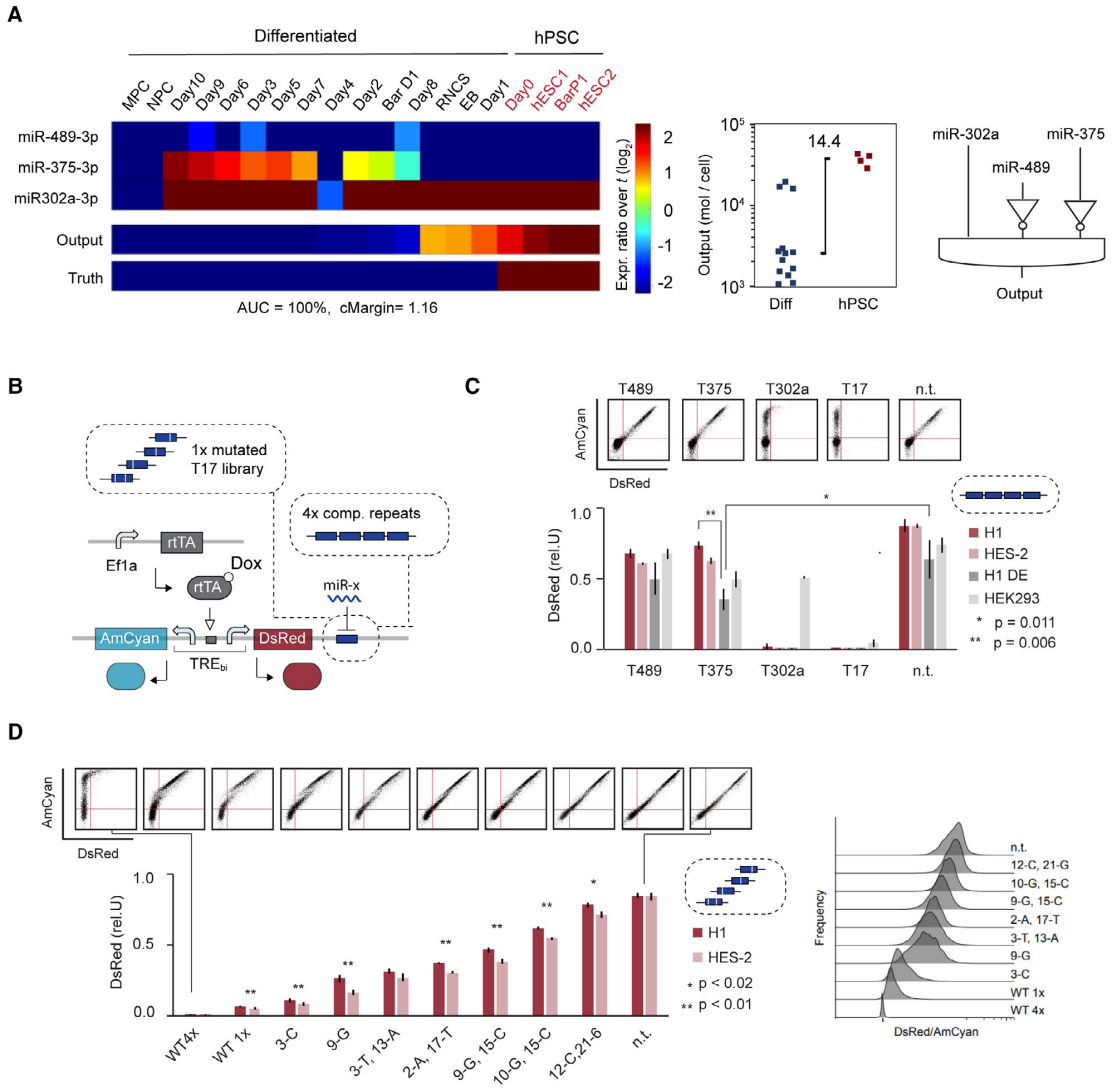
Automated circuit design and miRNA validation Summary of computationally identified circuit using a constraint of maximum three inputs. Shown are input miRNAs and predicted circuit output levels. Expression levels of identified miRNAs inputs are given as fold change over the pre‐set input abundance threshold (t) of the total miRNA pool (where *t* = 1%) (left). Calculated circuit output levels are given as mol/cell (middle) and logic connectivity of the identified miRNA is depicted (right). EB, embryoid bodies; MPC, mesenchymal progenitor cells; NPC, neural progenitor cells; R‐NSC, neural rosettes. miRNA expression data and nomenclature in Dataset EV1, raw output data and constraint files of the algorithm in Source data file. See also Fig EV1.Illustration of bidirectional miRNA sensor system.Bar chart showing relative DsRed expression of sensors containing the indicated miRNA target sites as four fully complementary repeats (discrete miRNA sensing). The control vector (n.t.) does not contain any target sites. Two‐tailed unpaired *t*‐tests were performed to compare the definitive endoderm derived from H1 (H1 DE) to H1 and the control vectors. For comparison to H1, data were normalized with the control vector (n.t.) before performing the *t*‐test. *P‐value = 0.011 and **P‐value = 0.006 are both considered very significant. Representative scatterplots of HES‐2 are shown on top.Bar chart showing relative DsRed expression of sensors containing different mutated target sequence of miR‐17 as listed in Appendix Table S1. Additionally, a sensor containing no target (n.t.) and two wildtype (WT) T17 target sites with 1× and 4× repeats were tested. Two‐tailed unpaired *t*‐tests were performed to compare H1 and HES‐2 for each sensor. *P‐values < 0.01, were considered very significant (**), < 0.02 significant (*) and > 0.5 not significant. Representative scatterplots of HES‐2 are shown on top. Data information: Each bar in (C) and (D) corresponds to mean ± s.d. from at least three biological replicates. See also Fig EV2. Source data are available online for this figure.

**Figure EV1:**
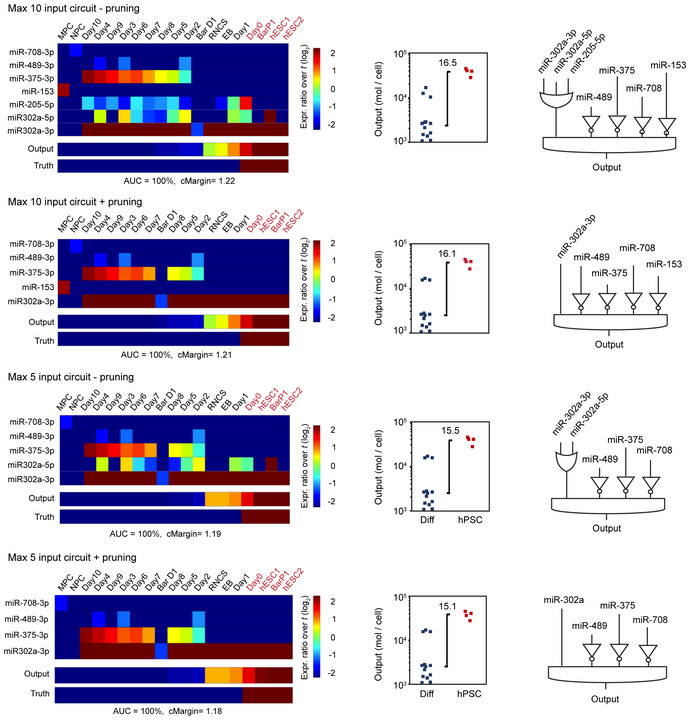
Summary of computationally identified circuits showing input miRNAs and predicted circuit output levels using different maximum input constraints Shown are identified circuits when ten or five maximum input number are used with or without pruning using miRNA data sets from Bar *et al* (2008a), Data ref: Bar *et al* (2008b), Lipchina *et al* (2011a), Data ref: Lipchina *et al* (2011b), Data ref: Fogel *et al* (2015a) and Fogel *et al* (2015b). Circuit identification using maximum of three input constraint is depicted in Fig 2A. Expression levels of identified miRNAs inputs are given as fold change over the pre‐set input abundance threshold (*t*) of the total miRNA pool (where *t* = 1%) (left). Calculated circuit output levels are given as mol/cell (middle) and logic connectivity of the identified miRNA is depicted (right). miRNA expression data and nomenclature can be found in Dataset EV1. Raw output data and constraint files of the algorithm in Source Data file. Related to Fig 2A. Source data are available online for this figure.

In order to apply the platform to identify hPSC‐specific miRNAs from different published miRNA sequencing sources, we have modified the miRNA pre‐selection step of the algorithm to allow selection of miRNA sequences instead of miRNA names (see Materials and Methods). Using three different data sets, covering four hPSC lines and 15 hPSC‐derived cell states (Fig 2A, Dataset EV1; Bar *et al*, 2008a; Data ref: Bar *et al*, 2008b; Lipchina *et al*, 2011a; Data ref: Lipchina *et al*, 2011b; Data ref: Fogel *et al*, 2015a; Fogel *et al*, 2015b), we identified a minimal set of miRNAs that allowed discrimination of hPSC from the other cell states with a cMargin of 1.16 corresponding to an average of 14.4‐fold change between the hPSC group and the differentiated group (Fig 2A). The algorithm identified three miRNAs: miRNA‐302a, which is highly expressed in hPSC and plays a critical role in maintenance of the pluripotent state (Lipchina *et al*, 2011a); and miR‐489 and miR‐375, which are not expressed in hPSC but are expressed at different levels in the negative samples (Fig 2A). miR‐375 is a key regulator during differentiation of hPSC toward pancreatic progenitors and mature beta‐ and alpha‐cells (Fogel *et al*, 2015b). The role of miR‐489 has been described in cancer but not, to the best of our knowledge, in hPSCs or during development. The underlying logic function the circuit performs can be approximated with: Output = miR‐302a AND NOT miR‐489 AND NOT miR‐375 (Fig 2A, right). By increasing the maximal circuit input number constraints and/or considering the unpruned circuit version, we found additional circuits with slightly improved performance (Fig EV1). We highlight that all identified circuits included miR‐302a, miR‐375, and miR‐489 among other miRNAs (Fig EV1), supporting the importance of the three miRNAs for hPSC classification. We also note that an additional highly expressed miRNA input forming an OR gate with miR‐302a might be beneficial for optimal circuit performance (Fig EV1).

**Figure EV2:**
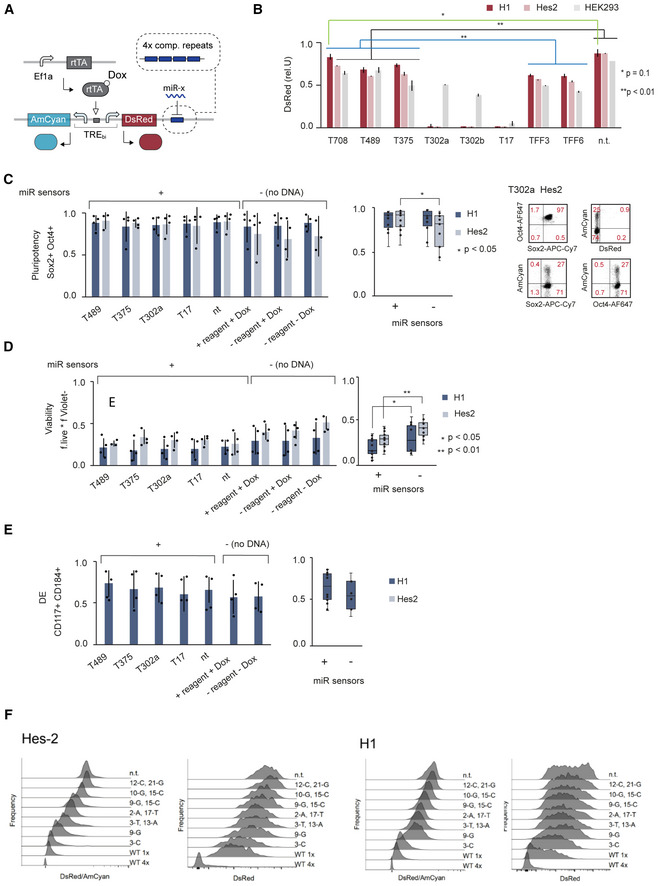
Characterization of computationally identified miRNA‐sensors on pluripotency marker expression, viability and endoderm differentiation in hPSC AIllustration of bidirectional miRNA sensor system.BBar chart showing relative DsRed expression of sensors containing the indicated miRNA target sites as four fully complementary repeats. The control vector (n.t.) does not contain any target sites. Two‐tailed unpaired *t*‐tests were performed to compare T708, T489 and T375 to TFF3, TFF6 or to n.t. for each cell line. *P*‐values < 0.01 were considered very significant (**), *P*‐value = 0.1 (*) was considered not significant. The bar groups samples with identical *P*‐values. Each bar chart corresponds to mean ± s.d. from at least three biological replicates. Related to Fig 2C.C–EBar chart left showing the fraction of Oc4^+^ Sox^+^ double‐positive pluripotent cells (C), viability (D) and CD184^+^ and CD117^+^ double‐positive cells marking definitive endoderm (DE) (D) for each miRNA sensor (+) and the untransfected samples (−) in different conditions as indicated. Bars show mean ± s.d. of at least three biological replicates. Individual samples are indicated as black dots. Box plot on the right shows all samples transfected with miRNA sensors (+) and all untransfected samples (−) in H1 and HES‐2. Box indicates first to third quartile range, horizontal line in box shows median, whiskers indicate the min‐max range and dots show inner data points (excluding min and max data point). Two‐tailed unpaired *t*‐tests were performed to compare the two sample groups. *P*‐values < 0.01 were considered very significant (**), < 0.05 significant (*) and > 0.5 not significant.FmisFITS library testing in hPSC. Histograms of the flow cytometry data shown in Fig 2D. Fluorescent intensity of DsRed (left) and DsRed normalized with internal AmCyan control (DsRed/AmCyan, right) shown for H1 and HES‐2 as indicated.

Next, we investigated if computationally identified miRNAs (miR‐302a, miR‐375, and miR‐489) and the fine‐tuner miRNA (miR‐17) showed expected activity in hPSC. For this, we analyzed the repression strength of each identified miRNA using a previously reported bidirectional miRNA reporter construct that expresses an untargeted internal control reporter AmCyan and an miRNA‐targeted reporter DsRed. Latter contains four fully complementary miRNA target sites in its 3′UTR (Xie *et al*, 2011) (Fig 2B). Upon transient transfection of the sensor system into hPSC lines H1 and HES‐2, we found that miRNA‐302a, which is highly expressed in hPSC (Fig 2A, Dataset EV1), can fully repress DsRed even when the construct is delivered at high copy number (Fig 2C). In contrast, reporters that detect miR‐489 and miR‐375, miRNAs that are not expressed in undifferentiated hPSC (Fig 2A, Dataset EV1), only slightly affected DsRed expression (Fig 2C). Our data show that sensors detecting miR‐489 and miR‐375 are expressed at significantly lower levels than a control construct that does not contain any target sites in DsRed (n.t.) but were significantly higher than constructs that contain mock miRNA target sites FF3 or FF6 (Rinaudo *et al*, 2007) that are not bound by any known endogenous human miRNA (Fig EV2B). Thus, we conclude that the observed small but significant changes in DsRed expression are likely due to differences in 3′UTR sequences and miR‐489 and miR‐375 are, as expected, not substantially active in hPSC. Additionally, we tested the same set of miRNA sensors in hPSC‐derived definitive endoderm (DE) and observed significant repression of the miR‐375 sensor, but no significant changes in miR‐489 and miR‐302a sensors (Fig 2C) in agreement with published expression levels (data labeled as Day 2 in Fig 2A, Dataset EV1). Finally, we tested HEK293 cells that show an miRNA expression profile similar to mesenchymal‐ and neural progenitor cells (MPC and NPC) in our data set (Fig 2A). As expected, the DsRed signal of all three sensors was not substantially repressed in HEK293 (Figs 2C and EV2B). Additionally, we characterized the effect of our transient transfection procedure using the miRNA sensors on viability, pluripotency state, and endoderm differentiation of hPSC (Fig EV2C–E).

We then used the same bidirectional sensor system to test if synthetic genes can be fine‐tuned in hPSC in a precise, step‐wise manner (Fig 2B and D). Specifically, we cloned eight variants selected from previously published miRNA‐17‐based misFITS library (Michaels *et al*, 2019), as a single target site into the 3′UTR of DsRed. Each variant differs from a perfectly complementary miR‐17 target site by one or two nucleotides (Appendix Table S1). We also tested 1× and 4× fully complementary miR‐17 target sites. Upon transient transfection, we measured DsRed expression relative to untargeted AmCyan in two hPSC lines (HES‐2 and H1). We found that each target variant leads to a distinct and defined DsRed expression level spanning the entire dynamic range from fully repressed to fully activated (Fig 2D). Compared with the HES‐2 line, the H1 line showed significantly higher DsRed expression for most sensors (Fig 2D).

In summary, we computationally identified, in an automated manner, a three‐input miRNA profile capable of discriminating hPSC from various differentiated cells, and show that miRNA expression data accurately predict relative repression strength of all three computationally identified miRNAs in hPSC, with near discrete sensing behavior. Further we show that endogenous miRNA‐17 target libraries (misFITS) allow production of exogenous reporter proteins at pre‐specified expression strengths in hPSC, thereby enabling protein tuning.

### Model‐guided combinatorial screening optimizes generic circuit performance in hPSC

**Figure 3:**
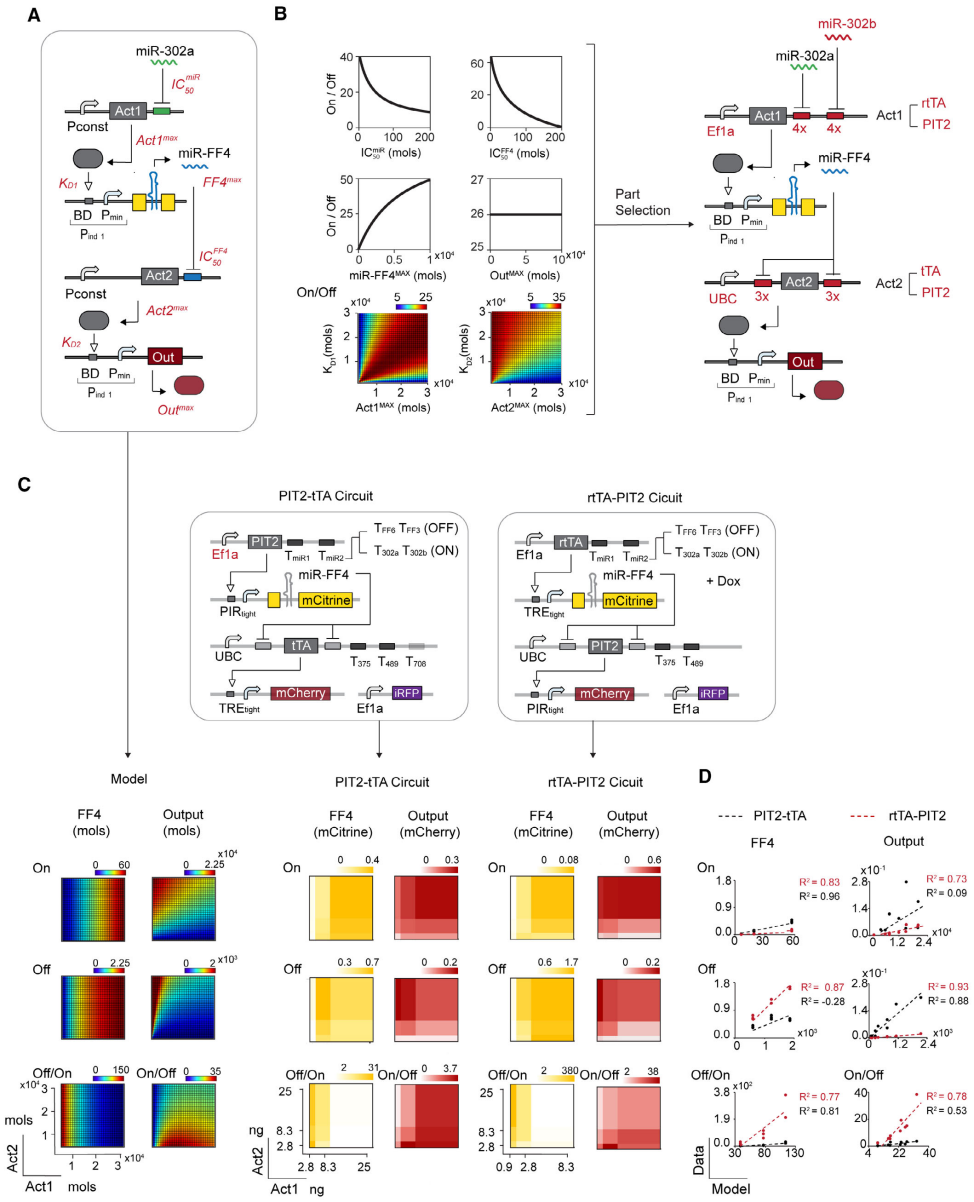
Model‐guided combinatorial screening optimizes generic circuit performance in hPSC Circuit schematic depicting the miR_high_ sensor within the whole bow‐tie circuit. The miR_high_ sensor recognizes miR‐302a in the 3′UTR of a constitutively expressed synthetic transactivator (Act1). Act1 induces a synthetic miRNA (miRFF4) that in turn represses a constitutively expressed transactivator Act2. Act2 induces expression of an output protein (here mCherry). In red: key parameters of each reaction. Act1_MAX_ and Act2_MAX_ are the maximal asymptotic expression achieved by the constitutive promoter driving the activator. Out_MAX_ and FF4_MAX_ are the maximal asymptotic expression when the inducible upstream promoter is fully activated. ${\mathrm{IC}}_{50}^{\mathrm{miR}}$ and ${\mathrm{IC}}_{50}^{\mathrm{FF}4}$ represent miRNA concentrations that lead to 50% knockdown in production of the protein targeted by the given miRNA. Associated equations in Materials and Methods.Dose–response curves of individual parameter screens (right, top and middle) and color plots of two‐parameter screens (right, bottom) for calculated On/Off ratio. Part selection based on parameter screen (highlighted in red).Combinatorial screen. Computational combinatorial screening of Act1_MAX_ and Act2_MAX_ using the model depicted in (A) (left). Experimental combinatorial screening in H1 (middle) with circuit schematic on top. Two different Act1‐Act2 circuit configurations are depicted: PIT2‐tTA (left) and rtTA‐PIT2 (right). Calculated FF4 and Output levels are shown for On, Off and Off/On (FF4) or On/Off (Output). Color plots show normalized mCitrine (left) and mCherry (right) expression levels for each Act1‐Act2 combination. Color bars show lowest and highest expression range on a linear scale with median as the midpoint.Correlation plots with calculated values on *X* axis (Model) and experimental data on *Y* axis (Data). A linear regression model (*y* = *a* + *b* × *x*) was used to compare experimental data with model predictions. Linear fit and coefficient of determination (*R* ^2^) as indicated. PIT2‐tTA circuit (black), rtTA‐PIT2 circuit (red). See also Fig EV4. Source data are available online for this figure.

While miR_low_ sensors typically achieve high dynamic ranges (Xie *et al*, 2011; Haefliger *et al*, 2016; Dastor *et al*, 2018), it has been difficult to engineer miR_high_ sensors (here to detect miR‐302a) with high dynamic ranges within the full bow‐tie configuration (Xie *et al*, 2011; Lapique & Benenson, 2014; Prochazka *et al*, 2014; Schreiber *et al*, 2016; Fig 3A). Reasons include the length of the signaling cascade, the unfavorable dynamics of certain components, and substantial variation in expression strength, growth rate, and transfection efficiency across different human cell types. Thus, to implement the generic circuit version, which uses computationally predicted miRNA inputs and requires a miR_high_ sensor, we undertook a model‐guided circuit optimization approach with the goal to increase the On/Off ratio (or dynamic range) of the miR_high_ sensor within an hPSC embedded bow‐tie circuit.

In line with previous models (Schreiber *et al*, 2016; Mohammadi *et al*, 2017), we modeled miRNA‐mediated repression and transcription‐factor‐mediated activation as non‐cooperative Hill‐like relationships between each upstream and downstream component. The model uses two kinds of parameters (Fig 3A, in red); parameters annotated with superscript MAX describe the uninhibited expression level of each protein. Parameters annotated with IC_50_ or K_D_ describe regulatory interactions. Specifically, Act1^MAX^ and Act2^MAX^ are the maximal asymptotic expressions achieved by the constitutive promoter driving the activator. Out^MAX^ and FF4^MAX^ are the maximal asymptotic expressions when the inducible upstream promoter is fully activated. ${\mathrm{IC}}_{50}^{\mathrm{miR}}$ and ${\mathrm{IC}}_{50}^{\mathrm{FF}4}$ represent miRNA concentrations that lead to 50% knockdown in production of the protein targeted by the given miRNA, where miR stands for endogenous miRNAs (here miR‐302a) and FF4 for synthetic miR‐FF4. K_D_ is the dissociation constant of synthetic activators (Act1 or Act2) for their respective promoters. The lower IC_50_ and K_D,_ the stronger the interaction. The model describes four reactions: (i) endogenous miRNA input (here miR302a)‐mediated Act1 repression (Act1^MAX^, ${\mathrm{IC}}_{50}^{\mathrm{miR}}$), (ii) Act1‐mediated FF4 production (FF4^MAX^, KD1), (iii) miR‐FF4‐mediated Act2 repression (Act2^MAX^, ${\mathrm{IC}}_{50}^{\mathrm{FF}4}$), and (iv) Act2‐mediated output production (Out^MAX^, KD2) (Fig 3A, equations and parameter description in Materials and Methods).

**Figure EV3:**
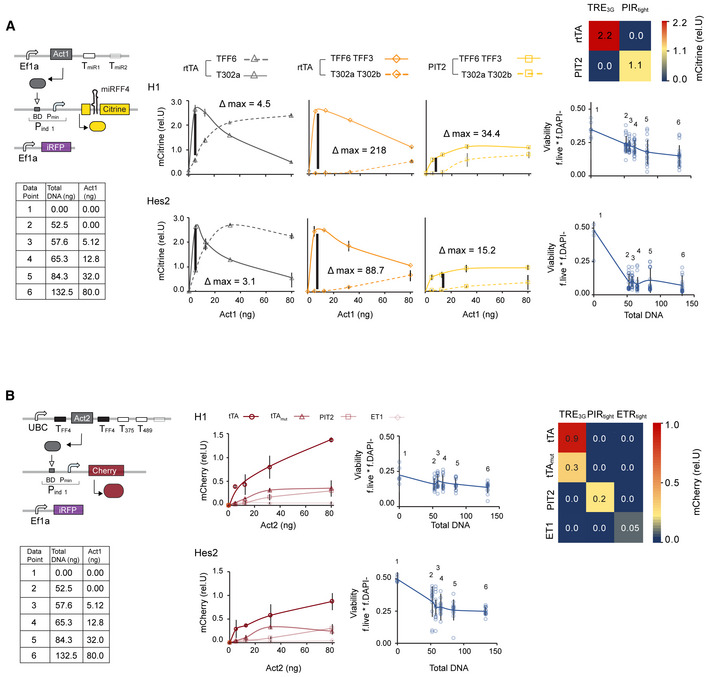
Circuit module characterizations A, BCharacterization of the FF4 inducible (A) and output inducible (B) module, respectively. Dose response function (middle) showing normalized fluorescence readouts of mCitrine and mCherry to changing Act1 and Act2 plasmid amounts, respectively. Curves serve as visual guides. Charts show the mean ± s.d. of at least three biological replicates. Viability as a function of DNA amount (left). Single data from all samples are plotted as circles. Mean values are indicated as dots. Line serves as a visual guide connecting mean values. Color plots show cross‐activation of each transactivator with each promoter using 32 ng of a given transactivator. Source data are available online for this figure.

Through parameter screening, we investigated the impact of each parameter on the dynamic range, which is the calculated On/Off ratio of the Output. The On and Off states were calculated with high physiological and zero miRNA input concentration, respectively (see Materials and Methods; Fig 3A and B and Appendix Fig S1). The model predicted that increasing miR‐FF4^MAX^ as well increasing Act1^MAX^ concentration leads to higher dynamic range. Therefore, we chose a strong promoter (Ef1a; Norrman *et al*, 2010) driving Act1 for our circuit design (Fig 3B and Appendix Fig S1). Next, the model predicted that increasing miR‐FF4 mediated repression of Act2, that is decreasing ${\mathrm{IC}}_{50}^{\text{miRFF}4}$, increases the dynamic range. Thus, we flanked Act2 with three fully complementary repeats of the miRFF4 target site in each the 5′UTR and 3′UTR of Act2, a design tested in previous studies (Schreiber *et al*, 2016; Gam *et al*, 2018) to increase miRFF4‐mediated repression (Fig 3B and Appendix Fig S1). The model also predicted that the dynamic range is higher at low Act2^MAX^ concentrations. Thus, we chose a relatively weak promoter (UBC; Norrman *et al*, 2010) to express Act2 (Fig 3B and Appendix Fig S1). Finally, the model predicted that increasing inhibition of Act1 by miR‐302a, that is decreasing ${\mathrm{IC}}_{50}^{\mathrm{miR}}$, increases the dynamic range (Fig 3B and Appendix Fig S1). Because this parameter is dependent on endogenous miRNA input activity, it is subject to variation, which led us to test different Act1 constructs driving FF4/mCitrine individually (Fig EV3). We found that Act1 targeted by four complementary repeats of miR‐302a alone (T302a) did not result in sufficient repression (Fig EV3A). To further decrease ${\mathrm{IC}}_{50}^{\mathrm{miR}}$, we designed new constructs that detect both miR‐302a and miR‐302b, where miR‐302b is, like miR‐302a, highly expressed in hPSC (Dataset EV1). This was done by placing four fully complementary target sites of both miRNAs in the 3′UTR of Act1, thereby forming an OR‐gate (T302a, T302b). We found that the OR gate highly increases miRF44/mCitrine repression (decreases ${\mathrm{IC}}_{50}^{\mathrm{miR}}$) and with that the dynamic range of miR‐FF4 expression compared with the construct that detects miR‐302a alone (Fig EV3).

**Figure EV4:**
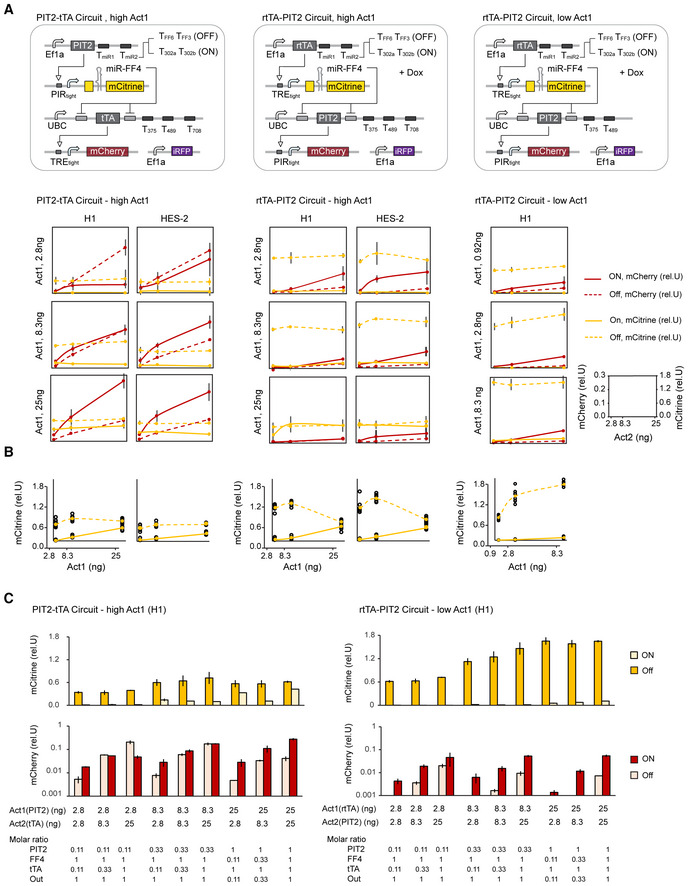
Extended analysis of experimental combinatorial screen Experimental combinatorial screening of Act1 and Act2 in three different circuit configurations: PIT2‐tTA using high levels of Act1 (left, data shown in Fig 3C), rtTA‐PIT2 using high levels of Act1 (middle) and rtTA‐PIT2 using low levels of Act1 (right, data shown in Fig 3C). For each circuit and each Act1 concentration, the normalized mCitrine/FF4 level (yellow) and the normalized Output levels (mCherry) (red) are shown in response to changing amount of Act2 expressing plasmids in the Off configuration (Act1 targeted by TFF3/TFF6) (solid lines) and On configuration (Act1 targeted by T302a/T302b) (dashed lines).Scatter plots showing normalized mCitrine expression in response to changing amounts of Act1 expressing plasmids across all Act2 levels of each circuit depicted in (A). Line connects mean values and serves as a visual guide.Bar chart of experimental data shown in Fig 3C. PIT2‐tTA circuit using high levels of Act1 (left) and rtTA‐PIT2 circuit using low levels of Act1 (right). Data information: All charts show mean ± s.d. of at least three biological replicates. Related to Fig 3C. Source data are available online for this figure.

Having identified the design of most key regulatory parts composing the generic circuit by single parameter screening, we were left with one combinatorial problem that required to be tested in a full circuit instantiation. Specifically, our model highlighted distinct but interconnected performance requirements of Act1 and Act2: Act1 achieved a high predicted On/Off ratio whenever KD or Act1^MAX^ was not particularly low. Act2, however, only led to a high predicted dynamic range when Act2^MAX^ was relatively low (Fig 3B and Appendix Fig S1). Thus, to identify the Act1‐Act2 combination that leads to highest dynamic range, we set up a combinatorial screen where we simultaneously changed the plasmid dosages of Act1 and Act2, which correspond to parameters Act1^MAX^ and Act2^MAX^ in our model (Fig 3C). Mathematical modeling of such a screen predicted that Act1^MAX^ affects FF4 production independently of Act2^MAX^, with the best Off/On performance when Act1^MAX^ is low (Fig 3C left). The model further predicted that Output production is affected by both Act1^MAX^ and Act2^MAX^ with the best On/Off ratio at intermediate Act1^MAX^ and low Act2^MAX^ (Fig 3C, left). Experimentally, we tested two circuit configurations with different combinations of transcription factors comprising Act1 and Act2, termed PIT2‐tTA‐ and rtTA‐PIT2 circuit (Fig 3C, middle). We combinatorically screened three different dosages of Act1 and Act2 plasmids each, resulting in nine Act1‐Act2 combinations for each circuit. The On state was generated by placing target sites for miR‐302a and miR‐302b (T302a T302b) in the 3′UTR of Act1; the Off state was simulated by adding mock miRNA target sites that are not responsive to any known human miRNA (TFF3 TFF6) in the 3′UTR of Act1 (Fig 3C, middle). FF4 and Output production were approximated by measuring mCitrine and mCherry expression, respectively (Fig 3C, middle). We observed strong inhibition of FF4/mCitrine expression at high doses of Act1 in the Off state when using rtTA as Act1 (Figs EV3A and EV4B), which is a result of cellular resource competition (Frei *et al*, 2020; Jones *et al*, 2020). Because of this inhibition, we used lower amounts of Act1 in the rtTA‐PIT2 circuit compared with the PIT2‐tTA circuit (see Source Data of Fig 3C).

To assess how accurately the data of the two different circuit configurations match our model predictions, we used a linear regression model and calculated the coefficient of determination (*R* ^2^) (Fig 3D). We found that rtTA‐PIT2 circuit performance correlates well with model predictions at all DNA amounts measured (*R* ^2^ > 0.73 in all cases; Fig 3D). In contrast, PIT2‐tTA circuit performance deviated from the model in FF4/mCitrine expression (Fig 3D), caused by inhibition of FF4/mCitrine expression at high Act1 concentration in the Off state (Figs 3C and EV4B), suggesting that lower Act1 levels would have been beneficial for this circuit configuration as well. However, even at lower Act1 levels, Output expression did not perform as expected within the PIT2‐rtTA circuit (Figs 3C and D, and EV4, data labeled with 2.8 and 8.2 ng Act1). Overall, the FF4/mCitrine levels were markedly lower and mCherry levels markedly higher in the PIT2‐tTA compared with the rtTA‐PIT2, consistent with observations from individual module testing (Fig EV3). Thus, the unexpected circuit behavior of PIT2‐tTA, as predicted by our mathematical model, resulted from an unfavorable combination of low FF4/mCitrine (low FF4^MAX^) and high Output production (due to low KD2 or high Act2^MAX^). Indeed, the best On/Off ratio for the PIT2‐tTA circuit was around ~4‐fold, while the best On/Off ratio for the rtTA‐PIT2 circuit was ~38‐fold. We note, however, that the high On/Off ratio in the rtTA‐PIT2 circuit comes at the cost of a very low On state (Figs 3C and EV4). As suggested by the model, the expression levels in the On state can be increased with increasing amounts of Act2, with the trade‐off of reducing the On/Off ratio (Fig EV4C). Overall, the rTA‐PIT2 circuit is the preferred circuit configuration because it shows computationally predictable signaling performance. We selected the optimal molar ratio of components rtTA:FF4:PIT2:Cherry to be 0.11:1:0.33:1, at which we achieved the second highest On/Off ratio and increased On state level compared with configuration with the highest On/Off levels (Fig EV4C).

To sum up, to optimize the dynamic range of the miR_high_ sensor in hPSC, we used a model‐guided part selection approach to design the core components of our generic circuit. Next, we applied a model‐guided combinatorial screen where computationally identified key parameters Act1^MAX^ and Act2^MAX^ were systematically and simultaneously changed within the entire generic circuit. By comparing two different Act1‐Act2 circuit configurations, this approach allowed us to quickly identify the circuit and DNA amounts that performed best in hPSCs according to design criteria.

### Generic circuit allows cell state specific output tuning in hPSC

**Figure 4:**
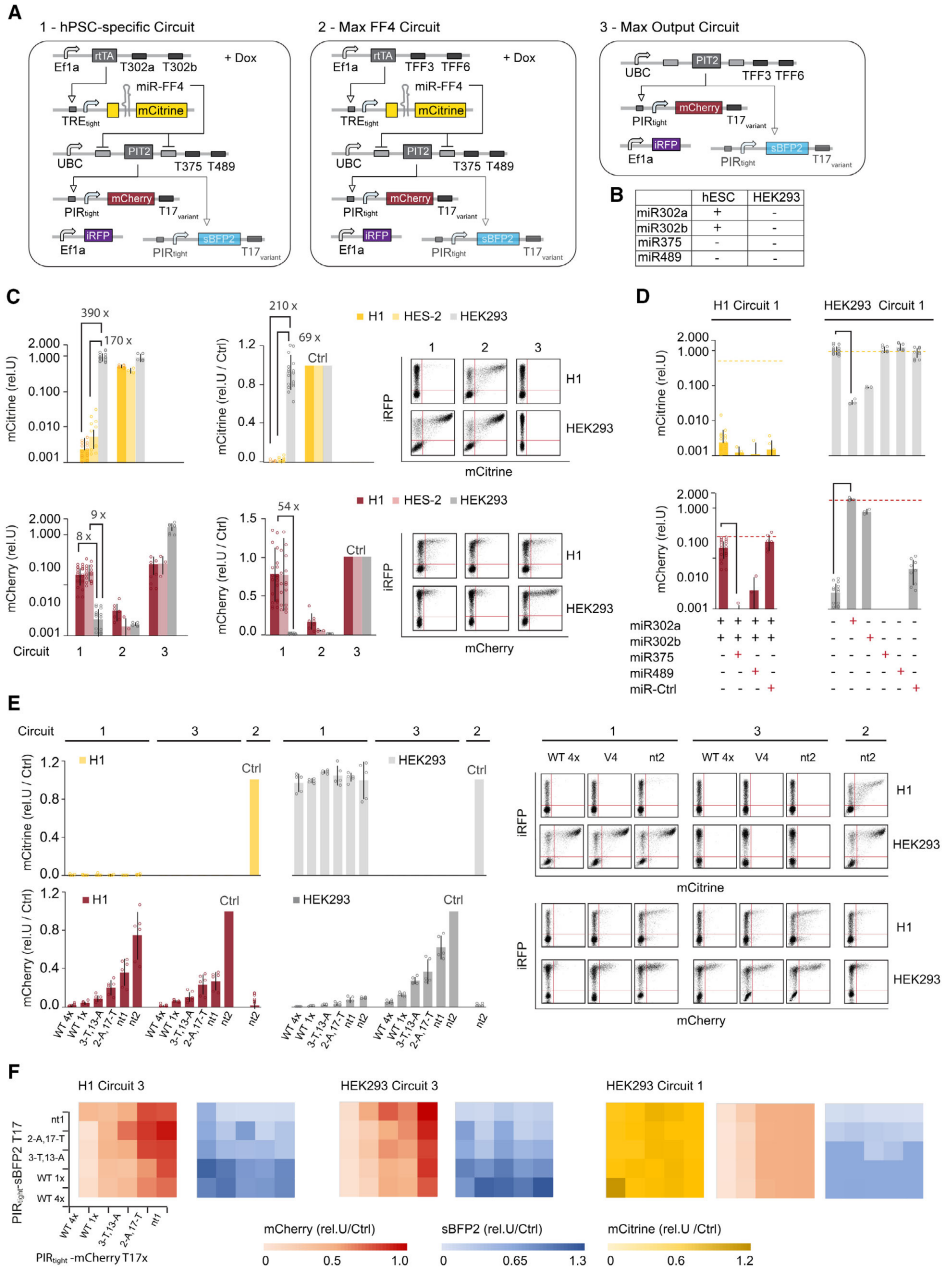
Generic circuit allows cell state specific output tuning in hPSC Circuit schematics depicting the hPSC specific circuit (circuit 1) recognizing miRNAs miR302a/b, miR375 and miR489 and two control circuits that measure the maximal levels of FF4/mCitrine (circuit 2) and the maximal output/mCherry level (circuit 3).Summary of the expression profile of hPSC and HEK293. See also Appendix Fig S2E.Bar chart showing the performance of circuit 1–3 in H1, HES‐2 and HEK293. mCitrine (top) and mCherry (bottom) expression was normalized to internal Ef1a‐iRFP control (rel.U.) (left) and further normalized to maximal mCitrine (circuit 2) (top) or maximal mCherry (bottom) (rel.U./Ctrl) (right). Samples below detection limit are labeled with b.d.Bar chart showing performance of circuit 1 in response to administration of miRNA mimics in H1 (left) and HEK293 (right). First sample (with profile + + − − − for H1 and − − − − − for HEK292) has no miRNA administered. For the other samples, miRNA dose with highest change in response has been selected from miRNA titration experiments (see Appendix Fig S2C). Maximal mCitrine and mCherry levels (as calculated in B) are depicted as yellow and red dashed lines, respectively. Chart is shown in a base 10 logarithmic scale.Bar chart showing fine‐tuning of output using miSFITs library. Shown are FF4/mCitrine (top) and Output/mCherry (bottom) of circuit 1 and 3 for different T17 variants. Data were normalized to control circuits.Color plots showing mean mCherry and mean sBFP2 expression normalized to control circuits. Color bars show lowest, median, and highest expression values. Data information: Each bar (in C–E) and color square (in F) show mean ± s.d. of at least three biological replicates. See also Appendix Fig S2. Source data are available online for this figure.

Having identified a circuit configuration with predictive miR_high_ sensor performance, we aimed to assess if our generic circuit could successfully distinguish between cell states on the basis of endogenous miRNA expression levels. To perform these experiments, we tested the better performing circuit rtTA‐PIT2 in hPSC lines H1 and HES‐2 and in HEK293 (Fig 4A–C). Specifically, we compared the circuit detecting the identified hPSC‐specific miRNA input profile (Fig 4A, circuit 1) to a circuit where T302a/T302b is replaced with TFF3/TFF6 to measure the maximal FF4/mCitrine production (Fig 4A, circuit 2), and a positive control circuit without miR_high_ sensor and mock miR_low_ sensors to measure the maximal Output/mCherry production (Fig 4A, circuit 3). Because HEK293 expresses substantially lower levels of miR‐302a/b compared with hPSCs (Figs 4B and EV2B), we expected them to express higher rtTA, leading to increased FF4 and reduced Output expression in HEK293 compared with hPSC. Indeed, our data show that HEK293 expresses FF4/mCitrine at a very high level, leading to at least 8‐fold higher repression of Output/mCherry compared with hPSC lines H1 and HES‐2 (Fig 4C, left). Normalizing the data for the observed promoter bias between the cell lines, we observed more than 50‐fold output repression in HEK293 compared to hPSCs (Fig 4C, right).

To further characterize circuit performance to changing miRNA amounts and testing of the full logic miRNA input profile, we measured FF4/mCitrine and Output/mCherry expression in response to externally administered miRNA mimics. We found that addition of miR‐375 and miR‐489 mimics each fully repress output production in hPSC (Fig 4D). However, we note that administration of miRNA mimics and siRNAs has proven difficult (Appendix Fig S2A–C) and LNA‐administration has be completely ineffective (Appendix Fig S2D) using our transient transfection setup in hPSC. Therefore, we have characterized the circuit performance in response to all input miRNAs in HEK293 (Fig 4D and Appendix Fig S2C). We found that administration of miR‐302a and miR‐302b can substantially repress FF4/mCitrine, resulting in full release of the Output expression confirming accurate signal processing of the miR_high_ sensor within the bow‐tie framework (Fig 4D and Appendix Fig S2C). Finally, consistent with observations in hPSC, miR‐375 and miR‐489 mimics can further reduce output production in HEK293, suggesting that cell types or cell states that differ in more than one miRNA from the hPSC‐specific profile will have a better off‐target behavior.

Having confirmed the accurate logic input signal processing, we evaluated if the output could be fine‐tuned within the whole circuit configuration. Because the output level in the On state is relatively low in hPSC, we increased the Act2 amount to 25 ng/96 well leading to a rTA:FF4:PIT2:mCherry molar ratio of 0.1:1:1:1. This resulted in a threefold increase in output level and a threefold decrease in On (H1)/Off (HEK293) ratio compared with previous circuit conditions used at 0.1:1:0.3:1 molar ratio between circuit plasmids (Appendix Fig S2E), consistent with previous characterizations (Fig 4C). When testing the positive control circuit with a small selection of the miR‐17 targeted misFITS library, we found that the output could be tuned in hPSC similarly to in HEK293 (Fig 4E, circuit 3), with the exception of one variant that behaved differently between the two lines (Appendix Fig S2F). As expected, we observed reduced output expression in HEK293, but not in H1, when using the PSC specific circuit for all tuners (Fig 4E, circuit 1). Because the highest On state is already weak, by futher tuning the levels down, we observed not only a shift in the mean value of the expression intensity but also in the number of cells that scored as mCherry positive (Fig 4E, scatter plots and source data for Fig 4).

Finally, we evaluated if we could independently tune two outputs by testing all combinations of miSFITs targeting mCherry and sBFP2 constructs, respectively. When testing circuit 3 in hPSC and HEK293, we found that mCherry and sBFP broadly follow the expected trend from low to high expression for each selected T17 variant (Fig 4F). However, compared with when a single reporter is used, repressive effects are observed when a variant is co‐expressed with another highly expressive variant, suggesting competition between PIT2 activators and/or cellular resources (Frei *et al*, 2020; Jones *et al*, 2020; Fig 4F, circuit 3). When testing the hPSC‐specific circuit (circuit 1) in HEK293, we found that FF4/mCitrine is strongly expressed and both outputs are significantly repressed in all output combinations compared with output levels in circuit 3 (Fig 4F, circuit 1), demonstrating that non‐hPSC‐specific circuit repression can be achieved for all variants in all combinations. Interestingly, we found that our two positive controls without T17 sites (n.t.1 and n.t.2) gave significantly different expression strengths (Fig 6E and F). Constructs n.t.1 and n.t.2 have identical promoter, CDS and polyA sequence, but differ in their backbone and UTR sequences; n.t.1 is identical to the library constructs but with fully complementary T17, while n.t.2 is the construct used in previous experiments (Appendix Table S2).

To sum up, our data demonstrate that the generic circuit produces output specifically in hPSC but not in HEK293 and is responsive to all four miRNA inputs. This provides evidence that logic integration of hPSC‐specific miRNA inputs, approximated with Output = miR‐302a OR miR‐302b AND NOT miR‐375 AND NOT miR‐489, is feasible. We also demonstrate that the discrete signaling can easily be tuned to desired protein levels with the use of miR‐17‐based miSFITs, allowing the independent tuning of at least two outputs.

### Optimization of minimal circuit

Thus far, we have demonstrated the rational model‐guided design of generic circuits detecting the hPSC state (hPSC On). Because an miR_high_ sensor is not always required to discriminate desired cell states, we next used an empirical approach to create a minimal circuit that operates only with miR_low_ sensors. Specifically, we used the inverted logic to repress the output in hPSC state (hPSC Off). Such circuits can be useful to eliminate hPSC in hPSC‐derived therapeutics or to avoid expression of effector molecules that are required to act at the later stages during hPSC differentiation.

**Figure 5:**
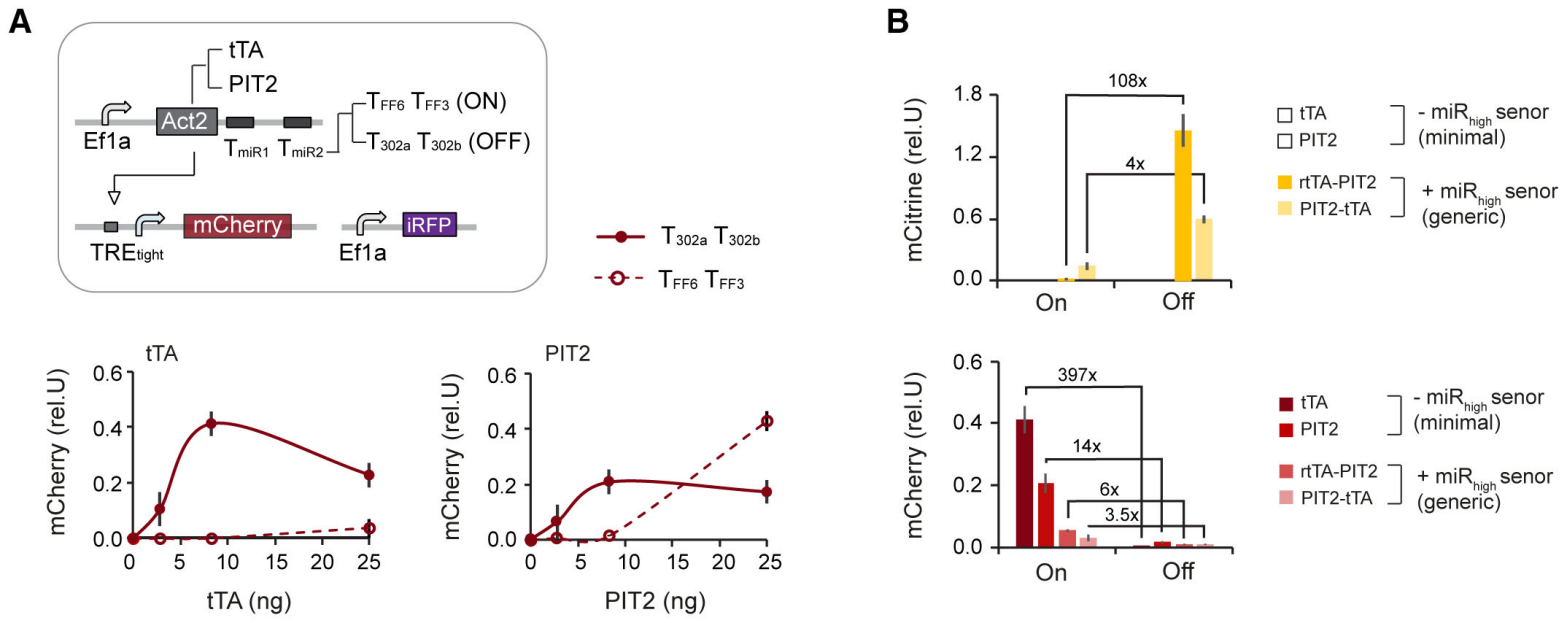
Optimization of minimal circuit Circuit schematics (left). Chart shows normalized Output production in response to changing amounts of plasmids encoding Act2, tTA (left) and PIT2 (right). Two transactivator constructs have been tested, one contains miR302a/b target sites (Off), the other by mock miRFF3/FF6 target sites (ON).Comparison of the maximal dynamic range across the two generic circuit versions and the two miR_low_ only circuit versions. For miR_low_ only circuits and PIT2‐tTA circuit, the configuration with highest dynamic range in Output has been selected. For rtTA‐PIT2 circuit, because of the low On state in the circuit with highest dynamic range, the circuit configuration with highest Off/On ratio in mCitrine/FF4 and highest On level in mCherry has been selected. Data information: Charts and color plots show mean ± s.d. of at least three biological replicates. Source data are available online for this figure.

To create the minimal circuit with inverted logic, we employed the previous miR_high_ miRNAs (miR‐302a and miR‐302b) as miR_low_ miRNAs by targeting the 3′UTR of Act2 with four complementary repeats of T302a and T302b target sites (Fig 5A). Thereby we created a circuit that is Off in hPSC computing the following logic operation Output = NOT(miR‐302a) AND NOT(miR302b). To simulate the On state, we targeted Act2 with mock TFF4 TFF6 target sites (Fig 5A). In this design, we used an Ef1a promoter to drive Act2, because the UBC promoter did not lead to saturation in activation of the downstream mCherry output (Fig EV3B) and to low output expression in the generic circuit (Fig 4C). We characterized two different Act2 constructs, Ef1a‐driven tTA and Ef1a‐driven PIT2 by measuring the output response to changing activator amounts in H1 using transient transfection (Fig 5A). We found that tTA outperforms PIT2, both in terms of the overall repression behavior in the Off state and the dynamic range. Best On/Off ratio lies at 397‐fold and 14‐fold for tTA and PIT2, respectively (Fig 5B). Interestingly, using PIT2 as Act2, the Off state at highest PIT2 amounts is higher than the On state, highlighting the challenges with high dose expression and associated cellular competition effects (Lillacci *et al*, 2018; Frei *et al*, 2020; Jones *et al*, 2020; Fig 5A). We note that the dynamic range of these minimal circuits with inverted logic largely exceeds generic circuits' performance that includes a miR_high_ sensor (Fig 5B).

To sum up, minimal circuits can be rapidly created and optimized with a single titration experiment. These circuits are not just easier to create but also largely exceed the On state expression and dynamic range compared to the generic circuits and are therefore recommended to use if the cell state can be effectively discriminated without miR_high_ miRNAs.

### Circuit‐mediated BMP4 secretion enables control over cell composition and pattern formation in micropatterned hPSC colonies

Next, we deployed the optimized minimal circuit in a proof‐of‐principle scenario to achieve control over cell fate specification and differentiation outcomes from hPSC. Specifically, we investigated if we could use our minimal circuit to control differentiated (endoderm, ectoderm, and mesoderm) cell compositions in response to circuit‐regulated developmental morphogen BMP4, which is typically administered exogenously to induce *in vitro* differentiation (Etoc *et al*, 2016; Tewary *et al*, 2017; Martyn *et al*, 2018; Minn *et al*, 2020). To do so, we evaluated our ability to control the secretion levels of BMP4 in the absence of exogenous BMP4 in micropatterned hPSC (RUES2) colonies.

**Figure 6:**
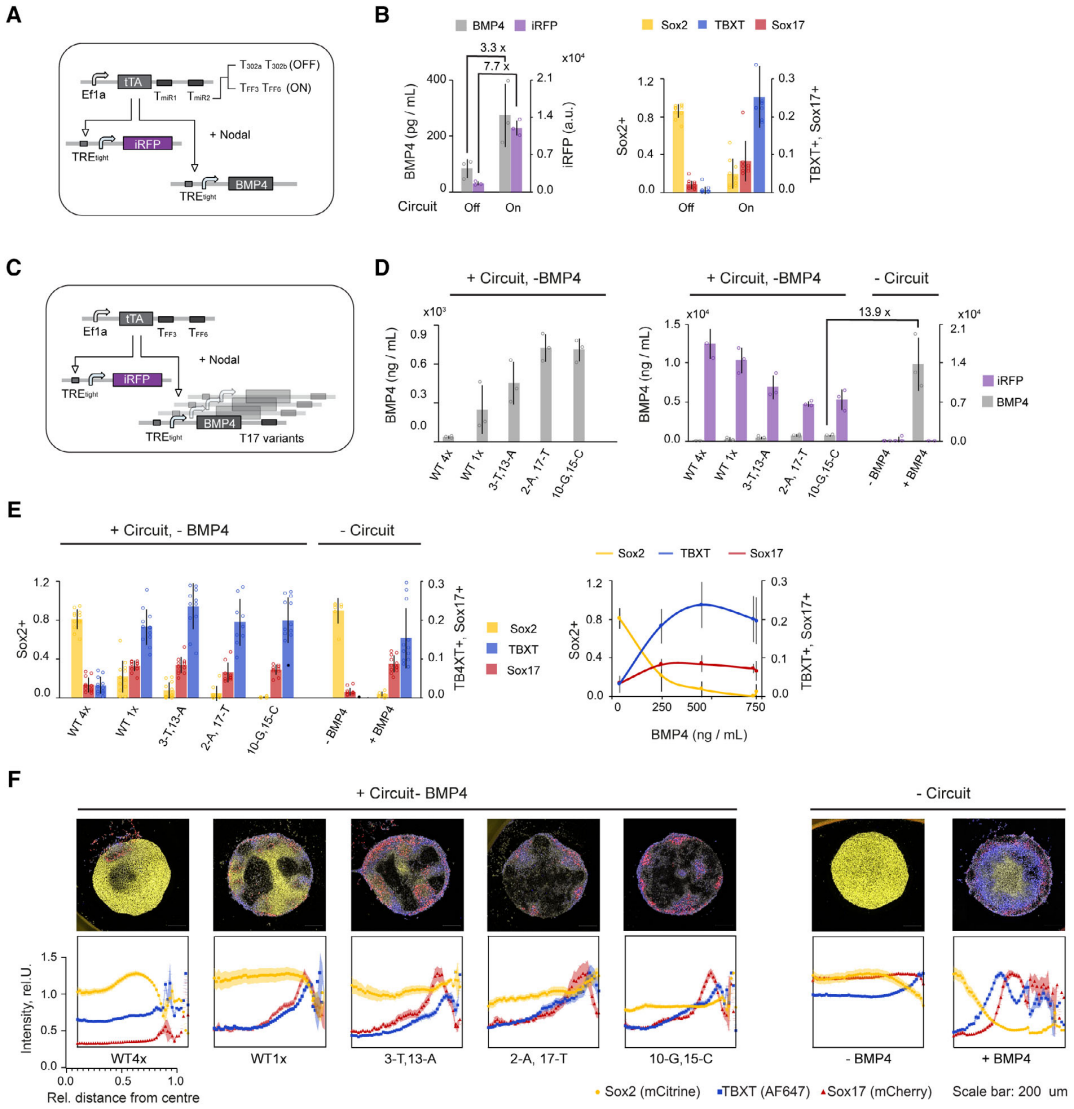
Circuit‐mediated BMP4 secretion enables control over cell composition and pattern formation in micropatterned hPSC colonies A, BDiscrete circuit‐mediated control of BMP4 by endogenous miRNAs. (A) Circuit schematic. Minimal circuit controlling BMP4 in response to the endogenous miRNAs miR‐302a/b target sites (Off) or mock target sites TFF3/TFF6 (On). (B) Micropatterned RUES2 have been transfected with On and Off circuits. Bar chart shows BMP4 concentration as determined by ELISA and average iRFP expression as measured by Flow Cytometry (left). Corresponding bar chart showing the average fraction of cells expressing a given germ‐layer marker (right).C–FCircuit‐mediated tuning of BMP4 = using miSFITs library. (C) Circuit schematic. Minimal circuit in mock On state controlling untargeted iRFP and miSFITs‐targeted BMP4. Five miSFITs variants have been transfected on micropatterned RUES2 cells. (D). Bar chart on the left shows BMP4 concentration for each circuit variant determined from culture media by ELISA. Bar chart on the right shows the same secreted BMP4 concentration along cell internal iRFP expression signal, measured by Flow Cytometry in comparison to untransfected controls in the absence (−BMP4) and presence (+BMP4) of BMP4 (right). (E) Bar chart showing the relative fraction of cells expressing a given germ‐layer marker as analyzed from confocal microscopy images (right). Dose response chart of the marker fraction in response to measured BMP4 concentration (left). See also representative Flow Cytometry scatter plots in Fig EV5C. (F) Radial marker profiles of each germ‐layer marker analyzed from confocal microscopy images. Charts on the left show normalized intensity of marker expression at a given colony position, where *x* = 0 is the center of the colony and *x* = 1 is the edge of the colony.Representative microscopy image of a single colony (top) showing composite of Sox2/mCitrine (yellow), Sox17/mCherry (red) and TBXT/AF647 (blue). Data information: Each bar show mean ± s.d. from at least three biological replicates for BMP4 and iRFP data and from at least 20 colonies for germ‐layer data. Scale bar represents 200 μm. See also Fig EV5. Source data are available online for this figure.

**Figure EV5:**
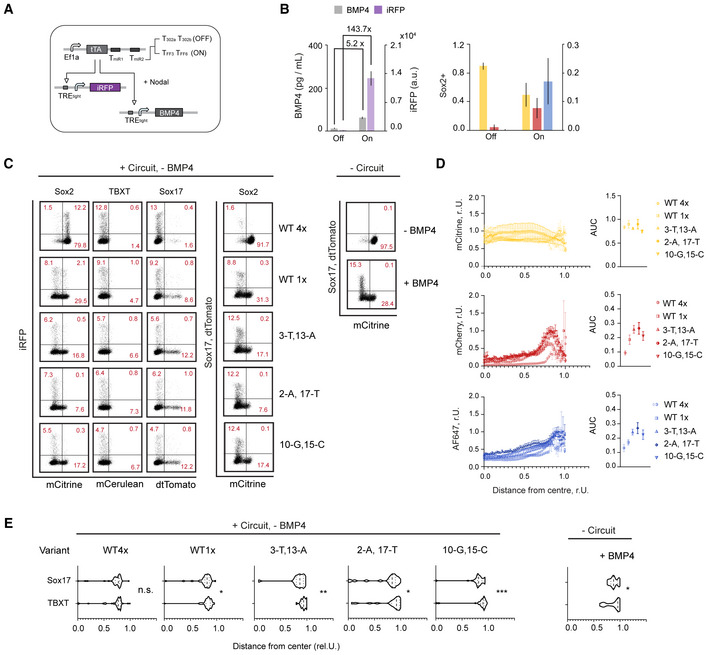
Extended analysis of BMP4‐mediated cell composition control Circuit schematic.Control of BMP4 in response to endogenous miRNAs. Micropatterned RUES2 have been transfected with in (A) illustrated On and Off circuits using tTA at 2.8 ng/96 well (in contrast, data in Fig 6B used tTA construct at 25 ng/96 well). Bar chart on the left shows average BMP4 concentration released from each circuit as determined by ELISA and absolute iRFP expression as measured by Flow Cytometry. Each bar corresponds to mean ± s.d. from three biological replicates. Bar chart on the right shows the average fraction of cells expressing a given germ‐layer marker analyzed from at least 20 colonies from Confocal Microscopy images. Related to Fig 6B.Representative Flow Cytometry scatter plots for microscopy data depicted in Fig 6D–F.Radial marker profiles of Sox2, TBXT and Sox17 for all miSFITs variants (left) and associated AUC values (right) of data depicted in Fig 6D–F.Spatial divergence of Sox17 and TBXT of data depicted in Fig 6D–F. Data information: Charts in (C–E) show average ± standard error calculated from at least 20 colonies.

First, we aimed to show that BMP4 production could be controlled in a discrete manner in response to endogenous miRNA inputs. For this, we used the best performing minimal hPSC Off circuit from Fig 5 (with tTA as Act2). We modified the circuit to control two outputs; BMP4 and iRFP, where the latter was used to identify transfected and BMP4 expressing cells (Fig 6A). Upon transient transfection into reporter hPSC line RUES‐2 (see Materials and Methods), we found that secretion of BMP4 was repressed 3.3‐fold when using the tTA construct containing miR302a/b targets (Off) compared with the tTA construct containing miRFF3/FF6 targets (On), whereas intracellular iRFP expression showed a 7.7‐fold change in On/Off ratio (Fig 6B, left). The dynamic range could be further increased when less tTA construct was used, with the trade‐off of achieving a lower On state (Fig EV5B). Analyzing the number of cells expressing a given germ‐layer marker, we found that the Off state, by repressing BMP4, locks the cells into a primarily Sox2 positive state (Fig 6B, right). In the On state, around 10% of cells express Sox17 and 10% TBXT, while the fraction of cells expressing Sox2 is less than 30%, indicating the differentiation into the three germ layers (Fig 6B, right).

Next, we investigated if we can tune BMP4 expression and secretion with our miSFITs library. For this, we designed a circuit in which Act2 controls a library of miSFITs‐targeted BMP4 constructs (Fig 6C). The circuit also controls iRFP that is not targeted by the miSFITs library, as a way to identify transfected cells. Consistent with our design expectations, we achieved graded levels of secreted BMP4 that saturated with the highest expressing tuners (2‐A,17‐T and 10‐G,15‐C V4) (Fig 6D, left). iRFP expression, on the other hand, decreased with increasing BMP4 expression, possibly due to cell resource limitations (Frei *et al*, 2020; Jones *et al*, 2020; Fig 6D, right).

We also tested whether different miSFITs variants encoding different BMP4 secretion levels could be used to control the relative composition of differentiated germ‐layer cells. We found that the cells adopted specific fates in a BMP4 dose‐dependent manner, where the proportion of ectoderm‐like Sox2^+^ cells continually and rapidly decreased and endoderm‐like Sox17^+^ and mesoderm‐like TBXT^+^ cells continually increased until all marker levels saturated as BMP4 levels saturated (Fig 6E). Interestingly, the apparent concentration of BMP4 required to achieve the same cell composition was at least 10 times lower when produced endogenously from circuit constructs (Fig 6D and E). In terms of cell composition of the germ layers, the variant with the lowest BMP4 level (WT4x) resembled untransfected controls in the absence of exogenous BMP4 (−BMP4) while variants with medium to high BMP4 levels resembled colonies with externally administered BMP4 (+BMP4) (Fig 6E and F).

To evaluate if circuit‐controlled BMP4 could induce gastrulation‐like pattern formation, we characterized the spatial marker expression from the colonies and compared the results with externally administered BMP4. For samples that were treated with externally administrated BMP4, we observed highly reproducible gastrulation‐like patterns as reported previously (Etoc *et al*, 2016; Tewary *et al*, 2017; Martyn *et al*, 2018; Minn *et al*, 2020). Gastrulation‐like patterns are concentric rings of germ layer markers with extra‐embryonic‐like tissue (CDX2^+^) at the colony edge (not measured here) followed by Sox17^+^, TBXT^+^, and Sox2^+^ cells radially distributed inward (Fig 6F). In contrast, colonies that expressed BMP4 endogenously from our circuit constructs appeared more heterogeneous and showed several sparsely populated regions that did not express any of the three germ‐layer markers (Fig 6F, left). Interestingly, BMP4 producing cells, as measured with iRFP, did not retain Sox2 expression when BMP4 was produced at medium to high levels and did not adopt an endoderm (SOX17^+^) or mesoderm (TBXT^+^) fate independent of the BMP4 concentration (Fig EV5C). Notably, despite the sparsely populated regions, colony‐wide patterns formed, with Sox17 and TBXT showing higher expression at the edge of the colony compared with the center (Fig 6F, bottom). The relative radial position of Sox17 and TBXT switched compared to samples treated with exogenous BMP4, with Sox17 closer to the center than TBXT (Figs 6F and EV5E). Additionally, Sox17 and TBXT had become more separated with increasing BMP4 production (Figs 6F and EV5E).

In summary, our data indicate that our minimal circuit can create different germ‐layer compositions from hPSC in a fine‐tuned manner with consistent BMP4 dose‐dependent cell fate changes using misFITs. Additionally, BMP4 production can be effectively repressed in response to cell‐state‐specific miRNAs, providing evidence that cell‐state‐specific and regulated control of secreted factors may be useful for the control of cell composition from differentiating hPSC. Further, we showed that recombinantly released BMP4 can trigger pattern formation in micropatterned hPSC colonies that are distinct from patterns previously reported using exogenous BMP4.

## Discussion

Cells constantly process signaling inputs in a cell state and context‐specific manner. Despite impressive achievements in the field of synthetic biology, rational engineering of artificial circuits that better represent the capabilities and complexity of natural biological computing systems remains challenging. Here we demonstrate gene circuits that are able to exert precise cell state‐specific control of desired proteins in hPSC. Specifically, we have implemented gene circuits that detect an hPSC‐specific miRNA profile and can produce up to two protein outputs that can be individually tuned to desired levels. Our circuits merge two critical functionalities: first, they allow restriction of circuit action to desired cell states, which is crucial for applications where conditional control of desired biomolecules is required; and second, they can produce the desired biomolecule at defined doses, which is important in many systems where the biomolecule exerts its function at precise physiological ranges. These functionalities can be useful for various applications but fit particularly well with the challenges of controlling developmental processes. During development, different intermediate cell states or lineages differentially control a small set of effectors or regulatory factors that operate at defined amounts. This happens through very complex, often poorly understood, interactions between gene regulatory networks (GRNs) and environmental signals (Tewary *et al*, 2018). Here we show that the same function can be achieved with a rationally engineered, synthetic, and programmable system that operates orthogonally in hPSCs and during early differentiation, opening the door to control the production and dose of desired natural or synthetic factors from desired cell states in emerging hPSC‐derived 2D and 3D tissues.

Classification of cell states and circuit design are nontrivial tasks. Here we have employed a previously established computational algorithm that automates the classification and circuit design procedure (Mohammadi *et al*, 2017). While the previous study examined the theoretical performance of this toolbox in the context of cancer classification (Mohammadi *et al*, 2017), we have demonstrated and expanded its usefulness in the context of stem cells and changing states during differentiation. Interestingly, it appears that undifferentiated hPSCs can be discriminated from different cell states with only a few miRNAs.

We have further demonstrated that a deterministic biochemical model can give useful and unexpected insights into the performance of a circuit of this complexity and can be helpful for reducing the number of genetic parts that need to be tested. However, we would like to note that the model used herein does not capture inter‐ and intra‐cellular variabilities and expression dynamics. To gain a more accurate understanding of the circuit, stochastic ODE models (Gillespie, 1977; Elowitz *et al*, 2002) or bin‐dependent ODE models designed for transient transfections (Wang *et al*, 2019; Stelzer & Benenson, 2020) are recommended. For deterministic ODE models of the bow‐tie circuit, we would like to refer to Haefliger *et al* (2016). Interestingly, the better performing circuit (rtTA‐PIT2 circuit) used the same transactivator combination as in our previous bow‐tie implementation in Hela cells (Prochazka *et al*, 2014). Because many differences exist between the pervious HeLa and this hPSC circuit implementation, such as a different version of miR_high_ sensor (here adopted from Schreiber *et al*, 2016), different miRNAs inputs, and different vector backbones, it suggests that the correct combination and levels of synthetic transactivators (Act1 and Act2) seem to be a main driver of optimal circuit performance and cannot easily be exchanged within the circuit architecture. It further suggests that the other parameters within the circuit have been sufficiently optimized within previous implementations (Xie *et al*, 2011; Prochazka *et al*, 2014; Schreiber *et al*, 2016). We also showed that creating a circuit version without miR_high_ sensors is dramatically less time‐consuming and leads to a higher dynamic range, though at the expense of reduced sensing capabilities. Therefore, we recommend an implementation scheme where the simplest circuit version proposed by the algorithm is selected, with preference for circuit input combinations that do not require miR_high_ sensors.

Further, our work demonstrated that the use of miSFITs (Michaels *et al*, 2019) is a straightforward approach to achieve defined output expression in hPSCs. We further documented that a few of the mutated T17 target sites behave differently in the context of a different 3′UTR and between different cell lines (hPSC vs HEK293), consistent with previous observations (Michaels *et al*, 2019). This suggests that either selection through screening or a comprehensive optimization of 3′UTR and backbones to enable consistent behavior across different cell types might be beneficial for certain applications. Importantly, we also demonstrate that miSFITs are an excellent option to tune multiple proteins independently in the same cell or sample. In fact, within our architecture, this would have not been possible with inducible systems that tune outputs using externally administered chemical inducers (such as pristinamycin for PIT2 or doxycycline for rtTA) (Gossen & Bujard, 1992; Weber *et al*, 2002; Szenk *et al*, 2020). Applying miSFITs for fine‐tuning also renders the system suitable for *in vivo* applications where chemical inducers are difficult to administer at defined levels and actuation is temporarily limited. Other means to change the dose, such as changing the transcriptional activity by modifying the Act2‐inducible promoter, is possible but inherently more difficult because it will generate a different dose–response function for output production, affecting the overall logic sensing performance and with this the entire system. Our data also revealed that precise tuning of two or more output genes using plasmid transfection is challenging because of cellular and circuit‐based resource competition. Recent studies characterized how synthetic genes compete with each other for cellular resources in human cells, with both computational models to capture and practical solutions to dampen this competition effect (Lillacci *et al*, 2018; Frei *et al*, 2020; Jones *et al*, 2020). Our results are consistent with a previous report of translational‐level resource redistribution by miRNAs, where miRNA suppression of one protein's translation increases production of other proteins (Frei *et al*, 2020). Future efforts to mitigate or accurately predict cellular resource competition will help improve overall circuit performance and the utility of miSFITs for tuning multiple genes independently.

Finally, we demonstrated that physiological factors such as BMP4 can be secreted into the cultivation media and fine‐tuned with miSFITs technology, enabling the control of differentiated cell compositions. Although transient transfection and the relatively low number of output‐producing cells may be a disadvantage for certain applications, we show that our setup is suitable for applications that aim to control secreted physiological factors, where the effect can be propagated across the population level. Further, we took advantage of context explorer (Ostblom *et al*, 2019) to rapidly quantify spatial marker expression in individual colonies and found that colony‐wide pattern formation could be achieved with circuit‐induced BMP4, despite short‐range diffusible effects and unequal cell density distribution, highlighting the strong self‐organization potential of hPSCs. Interestingly, circuit‐released BMP4 achieved differentiation and pattern formation at substantially lower concentrations compared with exogenously applied BMP4, creating opportunities to generate desired cell types in a manner that may more closely resemble natural developmental processes .

Moving forward, the computational and genetic tools developed here can be applied to generate new customized circuits that control not only desired secreted factors, but also transcription factors, small regulatory RNAs and other signaling and gene expression regulators from desired cell states or developmental stages. Our circuits, in combination with micropatterning, gastruloid, or organoid technologies, open the door to gain new insights into molecular mechanisms that guide pattern formation and other fundamental processes during early development or organogenesis. Additionally, our circuits can be applied to targeted killing of undesired cell types during differentiation, cell state tracking, re‐enforcement (or lock‐in) of desired cell states, direct conditional cell reprogramming *in vitro* and *in vivo*, and conditional production of therapeutic agents from hPSC‐derived cell products.

### Limitations

There are several key limitations that need to be addressed before the here implemented circuits can become widely applicable to program hPSCs, hPSC‐derived products, and other human cells. First, the current system works robustly with a high dynamic range only when miR_low_ sensors are used (minimal circuits), but produces very low yield when the input module comprises a miR_high_ sensor (generic circuits). Thus, the On state and dynamic range of the circuit output need to be further increased when miR_high_ sensors are required for cell state identification. This could be achieved with extended combinatorial screening using an extended library of genetic parts and new one‐pot circuit optimization techniques such as “poly‐transfection” (Gam *et al*, 2018). Further improvements can be achieved by optimizing the dynamics of the parts, such as delaying the production of Act2 with recombinase‐based systems, which can remove leakage in transient systems (Lapique & Benenson, 2014; Prochazka *et al*, 2014). Second, due to the long cascade of transcriptional components, the circuit is relatively slow in responding to changes. Thus, implementations of a circuit that aims to discriminate very close states may require faster turnover rate of the circuit proteins and/or stable integration of the circuit. Alternatively, faster‐acting RNA‐only (Wroblewska *et al*, 2015) or protein‐only (Gao *et al*, 2018) circuits could be merged with miSFITs technology. Third, the current transient transfection setup has several shortcomings, including high cell‐to‐cell variability and loss of plasmids over time, therefore limiting their applications. Stable integration of circuits of this complexity has proven challenging due to the low integration efficiency in hPSCs and because, once integrated, circuit silencing and unexpected cross‐activation from genome to the circuit or within circuit components can occur (Duportet *et al*, 2014; Fitzgerald *et al*, 2020). Finally, the large circuit size limits high‐efficiency viral‐based delivery strategies and with this their *in vivo* applications. With current fast development of genome editing tools, automated procedures, and new gene regulatory tools for mammalian cells, we believe these challenges can be overcome in the near future to enable straightforward use of our circuits for advanced differentiation control of hPSCs, direct reprogramming, basic research, and beyond.

## Materials and Methods

### Automated miRNA identification

miRNA expression data were obtained from published sources (Bar *et al*, 2008a; Data ref: Bar *et al*, 2008b; Lipchina *et al*, 2011a; Data ref: Lipchina *et al*, 2011b; Data ref: Fogel *et al*, 2015a; Fogel *et al*, 2015b). Cell source and miRNA expression data are summarized in Dataset EV1. miRNA sequencing data from (Fogel *et al*, 2015b) were renamed according to miRBase nomenclature, and mean values of replicates were calculated (Dataset EV1). In order to access miRNA expression data from different published sources that use different miRNA naming conventions, we modified the pre‐processing pipeline of the code developed by (Mohammadi *et al*, 2017). Instead of utilizing the miRNA names as provided by the sources, the provided miRNA sequences were determined by referring to the miRBase versions specified by the source papers. Furthermore, hairpin miRNA sequences were also obtained from the respective miRBase versions, and they were matched and eliminated from the dataset. Then, miRNA names, sequences, and sequencing data from the three sources were compared and combined to generate a merged dataset. Lastly, similar miRNAs within this merged dataset were combined just as Mohammadi *et al* (2017) did for a single dataset. The optimal input miRNA set was searched based on this finalized merged dataset.

The algorithm with the modified pre‐processing pipeline can be accessed on GitHub: https://github.com/jwon0408/SynNetModified.

To run the algorithm, total maximal miRNA inputs and the maximal miRNA input number of each gate have been modified as shown in Source Data of Fig 2 Raw output data files for Fig 2A can be found in Source Data of Fig 2.

The definitions of the circuits and quantitative search tool outputs are the following:

#### AUC

Area under the receiving operating characteristic curve. A measure to quantify the circuit output. It assesses circuit performance by scoring how well positive samples can be separated from negative samples.

#### cMargin

Classification margin. A circuit performance score assessing the ratio in output production between positive (here hPSC) and negative (here hPSC‐derived cell states) samples. Specifically, the cMargin is the calculated average of the following two margins: the average margin (the overall ratio between outputs in positive versus negative samplesets) and the worst margin (the smallest ratio among any pair of positive and negative samplesets).

#### Truth

Binary truth value that annotates each sample that leads to output production above the classification margin as true (1) and below the classification margin as false (0).

#### Expression ratio over *t*

The ratio of expression above or below the binarization threshold *t* given as miRNA copies/cell. The binarization threshold *t* was set to 1% of total miRNA abundance. Above this threshold, an miRNA is considered to be biologically relevant (Mohammadi *et al*, 2017).

#### Pruning

A post‐processing step using a bootstrap‐based algorithm that prunes off miRNAs that do not significantly improve overall circuit performance.

For more details on the model and definitions, we would like to refer to (Mohammadi *et al*, 2017).

### Modeling and simulation

Circuit modeling and simulations were performed in MATLAB. The model describes a miR_high_ sensor within a bow‐tie architecture detecting one miRNA‐input (miR‐302a) and controlling one protein output (as depicted in Fig 3A). The model was derived from (Schreiber *et al*, 2016) and assumes non‐cooperative Hill‐like relationships for miRNA‐mediated repression and transcription‐factor mediated activation.

The following four steady‐state equations were used to describe the circuit reactions: miRNA‐mediated Act1 repression (1), Act1‐mediated FF4 production (2), miR‐FF4‐mediated Act2 repression (3), and Act2‐mediated output production (4):

(1) $$\left[\mathrm{Act}1\right]\left(\mathrm{miR}\right)=\mathrm{Act}1^{\mathrm{MAX}}\left(1-\frac{\left[\mathrm{miR}\right]}{{\mathrm{IC}}_{50}^{\mathrm{miR}}+\left[\mathrm{miR}\right]}\right),$$

where [Act1] is the calculated steady‐state concentration of the transactivator; Act1^MAX^ is the maximal steady‐state activator concentration, without any miRNA‐mediated repression. [miR] stands for miRNA input concentration, and ${\mathrm{IC}}_{50}^{\mathrm{miR}}$ for input miRNA concentration that elicits half the knockdown.

(2) $$\left[\mathrm{FF}4\right]\left(\mathrm{Act}1\right)=\mathrm{miR}‐\mathrm{FF}4^{\mathrm{MAX}}\left(\frac{\left[\mathrm{Act}1\right]}{K_{D1}+\left[\mathrm{Act}1\right]}\right),$$

where [miR‐FF4] represents steady‐state concentration of miR‐FF4, [Act1] is the activator level computed with equation (1), K_D1_ is the dissociation constant of Act1 from its promoter. miR‐FF4^MAX^ is the maximal expression level of miR‐FF4 from an inducible promoter under transactivator saturation.

(3) $$\left[\mathrm{Act}2\right]\left(\mathrm{miR}‐\mathrm{FF}4\right)=\mathrm{Act}2^{\mathrm{MAX}}\left(1-\frac{\left[\mathrm{miR}‐\mathrm{FF}4\right]}{{\mathrm{IC}}_{50}^{\mathrm{FF}4}+\left[\mathrm{miR}‐\mathrm{FF}4\right]}\right),$$

where [Act2] is the steady‐state concentration of the central knot activator; Act2^MAX^ is the maximal steady‐state activator concentration without any miRNA‐mediated repression. [miR‐FF4] is the miR‐FF4 concentration calculated in equation (2), and ${\mathrm{IC}}_{50}^{\mathrm{FF}4}$ is the miR‐FF4 concentration that elicits half the repression.

(4) $$\left[\text{Out}\right]\left(\mathrm{Act}2\right)={\text{Out}}^{\mathrm{MAX}}\left(\frac{\left[\mathrm{Act}2\right]}{K_{D2}+\left[\mathrm{Act}2\right]}\right),$$

[Out] is the steady‐state concentration of the circuit output, and Out^MAX^ is the maximal output concentration from an inducible promoter under Act2 saturation. [Act2] is the activator concentration calculated in (3). K_D2_ is the dissociation constant of Act1 from its promoter.

The parameters for numerical simulations (Fig 3 and Appendix Fig S1) were derived from Mohammadi *et al* (2017). Specifically, we used their optimized parameter set: ${\mathrm{IC}}_{50}^{\mathrm{miR}}$: 20 molecules/cell, ${\mathrm{IC}}_{50}^{\mathrm{FF}4}$: 20 molecules/cell, K_D1_ = K_D2_: 10,251 molecules/cell, Act1^MAX^ = Act2^MAX^: 9,755 molecules/cell, miR‐FF4^MAX^: 3,000 molecules/cell, and Out^MAX^ 30,000 molecules/cell. The ON state was simulated with [miR] = 3,000 molecules/cell and the Off state with [miR] = 0 molecules/cell. For each parameter scan, the varied parameters were tested in the range indicated in the plot and the fixed parameters were set at the numbers indicated above. For the correlation plot in Fig 5D we used 30,000, 10,000, and 3,333 molecules per cell for both Act1^MAX^ and Act2^MAX^ to calculate FF4 and output production.

### Cloning

Bidirectional miRNA sensor plasmids were cloned using standard cloning techniques. Briefly, different miRNA target sites (Appendix Table S1) were designed with SalI and NotI overhangs, ordered as ssDNA from Sigma Aldrich, annealed by temperature decrease from 95 to 4°C, and phosphorylated using T4 Polynucleotide Kinase (NEB, M0201) according to manufactures' protocol. Annealed fragments were purified using the GenElute™ PCR Clean‐Up Kit (Sigma Aldrich, NA1020). Bidirectional precursor construct (pZ073) Xie *et al*, 2011 obtained from Benenson lab at ETH Zurich was digested with 30 U of SalI‐HF (New England Biolabs (NEB), R3138S) and 30 U of NotI‐HF (NEB, R3189) using CutSmart Buffer in a total reaction volume of 50 μl for fours at 37°C. Digested backbone was purified from 0.7% Agarose Gel using the GenElute™ Gel extraction Kit (Sigma Aldrich, NA1111). Vector and miRNA target inserts were ligated at 1:10 molar ratio using 400 U of T4 DNA Ligase (NEB, M0202) using provided Ligase buffer in a reaction volume of 20 μl over night at 16°C. Then 4 μl of ligation mix was transformed into 50 μl of home‐made chemically competent *Escherichia coli* DH5alpha obtained from New England Biolabs (C2987I) and selected on LB Agar plates containing 100 μg/ml Ampicillin (Sigma‐Aldrich, A0166‐25G).

All remaining plasmids were generated using Goldengate cloning with the MoClo toolkit (Addgene Kit # 1000000044) as described in (Weber *et al*, 2011). Briefly, level 0 constructs were generated by cloning a given part (promoter, miRNA target sites, spacer sequences, protein coding sequences, or polyA) into level 0 destination vectors from the MoClo toolkit or into home‐made level 0 vectors that can restore the Kozak sequence (Appendix Table S2). The DNA parts were either (i) ordered as dsDNA from Twist Bioscience, (ii) as ssDNA oligos from IDT Technologies or Sigma Aldrich, annealed and phosphorylated as described above or (iii) PCR amplified from other vectors using Phusion® DNA polymerase (NEB, M0530L) according to manufactures’ protocol. PCR fragments were analyzed using Agarose Gel Electrophoresis and purified from Gel using the GenElute Gel Extraction Kit (Sigma, NA1111) or MinElute Gel Extraction Kit (Qiagen, 28604) according to manufactures’ protocol. Cloning of level 0 vectors was performed with 40 fmol of level 0 backbone and 40 fmol of DNA insert using 10 U *BpiI* (Thermo Scientific, ER1011), 400 U T4 Ligase (NEB, M0202S), 0.15 μl BSA (NEB, B9000S), and 1.5 μl T4 ligation buffer (NEB, B0202S) at a final volume of 15 μl. Assembly was performed using the following cycle condition: 37°C 15 min (1 cycle), 37°C 2 min followed by 16°C 5 min (50 cycles), 37°C 15 min, 50°C 5 min, 80°C 5 min, 4°C until use. Then 4 μl of the MoClo mix was transformed into 50 μl NEB 5‐alpha competent *E. coli* (NEB, C2987I) or into 50–100 μl home‐made chemically competent *E. coli* 5‐alpha. Clones were selected with *lacZ*‐based blue‐white screening method on LB agar plates containing 100 μg/ml Ampicillin (Sigma‐Aldrich, A0166‐25G), 100 μM IPTG (Sigma‐Aldrich, I6758‐5G), and 40 μg/ml X‐gal (BioShop Canada, XGA001.1).

Expression units were assembled from cloned level 0 parts into level 1 destination vectors provided by the MoClo kit using 40 fmol of each vector and 20 U of *BsaI‐HFv2* (NEB, R3733L) with remaining conditions as described above. Clones were selected based on the same *lacZ* selection strategy as described above but with 50 μg/ml Spectinomycin (BioShop Canada, SPE201.5) instead of Ampicillin for selection.

All vectors used for assembly and transfections were purified with GeneJET plasmid miniprep kit (Thermo Fisher Scientific, K0503) or GenElute™ Plasmid MiniPrep Kit (Sigma, PLN70). Sequence of every cloned plasmid was verified using Sanger Sequencing performed at the Centre for Applied Genomics at the Hospital for Sick Children, University of Toronto or at Genewiz Inc. Prior to transfections, plasmid quantity was determined using Nanodrop and quality was verified on 0.6–1% Agarose Gels. A complete list of plasmids cloned in this study can be found in Appendix Table S2. Constructs that are not restricted for sharing under MTA are provided on Addgene.

### siRNA, miRNA mimics, and miRNA inhibitors

MiRNA mimics were obtained from Dharmacon as Human miRIDIAN microRNA Human mimics. Hsa‐miR‐302a‐3p (C‐300653‐05‐0002), hsa‐miRNA‐302b‐3p (C‐300669‐05‐0002), has‐miR‐489‐3p (C‐300749‐07‐0002), hsa‐miR‐375 (C‐300682‐05‐0002) and miR‐Ctrl Negative Ctrl #1 (CN‐001000‐01‐05). siRNA FF4 was custom‐designed as Silencer™ Select siRNA from Thermo Fisher Scientific (see Rinaudo *et al*, 2007 for sequence) and compared with siRNA Ctrl: Silencer Select Negative Control No. 1 siRNA (Thermo Fisher Scientific, 4390843). miRNA inhibitors were purchased from Qiagen as miRCURY LNA miRNA inhibitors: LNA‐miR‐302a‐3p (YI04100713‐ACA), LNA‐miR302b‐3p (YI04101540‐ACA) and used in comparison to the Negative Control B LNA (YI00199007‐ADA).

### Cell culture

hPSC lines H1 (WAe001‐A) (Thomson, 1998) and HES‐2 (ESIBIe002‐A) (Reubinoff *et al*, 2000) were obtained from Christina Nostro and Gordon Keller lab at the University of Toronto, respectively. H1 and HES‐2 were thawed and maintained on irradiated mouse embryonic fibroblasts (MEF) and cultured for 6 days in maintenance medium comprising Dulbecco's minimum essential media DMEM/F12 (77.5% v/v, Gibco), Knockout Serum Replacement (20% v/v, KOSR, Gibco), GlutaMax™ (2 mM, Invitrogen; 35050061), penicillin/streptomycin (0.5% v/v, Gibco; 15140122), non‐essential amino acids (1% v/v, Gibco), β‐mercaptoethanol (0.1 mM, Sigma), and 20 ng/ml basic fibroblast growth factor (bFGF; PeproTech). Cells were maintained at 37°C humidified air with 5% CO_2_ with daily medium exchange. Before transfection, cells were transferred and grown in Essential 8™ Medium (Gibco, A1517001) supplemented with 0.5% Penicillin/Streptomycin Solution (Gibco; 15140122) on 2% Geltrex™ (Gibco, A1413302) coated plates at a split ratio of 1:6 for at least one passage. Media was exchanged daily, and splitting was performed every 4–5 days when cells reached 75–85% confluency using TrypLE™ (Gibco, 12605028). Cultures were propagated for at most four passages before being replaced by fresh cell stock.

The hPSC RUES2 line (Martyn *et al*, 2018) was obtained from Prof Ali Brivanlou's lab at the Rockefeller University. This line is genetically engineered to co‐express fluorescent reporters with the following germline markers SOX2‐mCitrine, SOX17‐dtTomato, and TBXT‐mCerulean. The cell line was cultured using Geltrex (Life Technologies, Catalog # A1413301, 1:50 dilution) and mTeSR medium (StemCell Technologies, Catalog # 85850) without penicillin/streptomycin. Cells were clump passaged using ReLeSR (Stem Cell Technologies, Catalog # 05872) and maintained at 37°C with 5% CO_2_. Media was changed daily, and cells were passaged once they reached 75–80% confluence.

HEK293 cells were obtained from Princess Margaret University Health Network (Pan's lab) and cultured at 37°C, 5% CO_2_ in DMEM (Gibco; 11965092), supplemented with 10% fetal bovine serum (FBS; Gibco; 12483020), 1% GlutaMAX™ (Gibco; 35050061) and 1% Penicillin/Streptomycin Solution (Gibco; 15140122). Splitting was performed using 0.25% Trypsin–EDTA (Gibco; 25200072) every 2–3 days until cells reached 90–100% confluency.

All cell lines have been tested for Mycoplasma at the Hospital for Sick Children. hESC H1 and HES‐2 have been karyotyped at Thermo Fisher Scientific and WiCell.

### Transfections

All transfections of hESC H1 and HES‐2 were performed using Lipofectamine Stem Transfection Reagent (Thermo Fisher Scientific, STEM00015). For transfections in pluripotency state, cells were dissociated into single cells with TrypLE™ for 4–5 min at 37°C and seeded into 2% Geltrex‐coated 96‐ or 12‐well plates (Fisher Scientific) in Essential 8™ Medium (Gibco, A1517001) supplemented with 0.5% Penicillin/Streptomycin Solution (Pen/Strep, Invitrogen; 15140122) and ×10 μM Y27632 Rho‐rock inhibitor (Reagents Direct, 53‐B85). 1–1.2 × 10^4^ cells were seeded into 100 μl culture media per 96‐well for transfections without antibody staining and 1 × 10^5^ cells were seeded into 500 μl culture media per 12‐well for transfections with subsequent antibody staining. Cells were incubated at 37°C, 5% CO_2_ for around 24 h until transfection was performed at around 55–65% confluency. For transfections with subsequent differentiation into endoderm lineage, cells were seeded as patches into 12‐well plates on 2% Geltrex using NutriStem® hESC XF (Biological Industries, 05‐100‐1A) supplemented with 0.5% Penicillin/Streptomycin Solution. Cells were grown for 2–3 days before transfection was performed at around 90% confluency. At the day of transfection, medium was replaced with fresh growth medium (Essential 8 or Nutristem respectively) supplemented with Doxycycline hyclate (Dox, Sigma, D9891) at a final concentration of 0.8 μg/ml if required. Plasmid DNA and miRNA mimics, if needed, were mixed as indicated in Source Data files. Lipofectamine Stem Reagent was diluted 25‐fold with Opti‐MEM I Reduced Serum (Life Technologies 31985‐962). For each 96‐well and 12‐well sample, 10 and 100 μl respectively of diluted reagent was mixed with the DNA mixture, incubated for 10–12 min at room temperature, and added to the cells. For samples in pluripotency state, medium was replaced after 24 h with fresh Essential 8™ including Pen/Strep and Dox if needed but no Rho‐Rock inhibitor using a volume of 150 μl and 1.5 ml per 96‐well and 12‐well, respectively. Cells were harvested for flow cytometry and antibody staining 48 h after transfection. For differentiations, medium was replaced 24 h after transfection with endoderm‐inducing media (see below).

### Endoderm differentiations

hESC H1 cells were seeded from MEF condition into 12‐well plates and grown in Nutristem hESC XF (Biological Industries, 05‐100‐1A) on 2% Geltrex for 1–2 days until they reached 90% confluency. Some samples were transfected using Lipofectamine Stem Reagent as described above, untransfected samples were kept in fresh Nutristem. Twenty‐four hours after transfection (= Day0), endoderm differentiation was initiated. At day 0, pluripotency medium was replaced with endoderm base medium (RPMI1640 (Gibco, 11875093), 1% GlutaMAX™, 1% penicillin–streptomycin solution) supplemented with 2 μM CHIR 99021 (Reagent Direct, 27‐H76) and 100 ng/ml Activin A (home‐made). After 24 h_,_ medium was replaced with endoderm base medium supplemented with 100 ng/ml Activin A, 50 μg/ml Ascorbic Acid (Sigma‐Aldrich; A8960), and 5 ng/ml human FGFb (Peprotech; 100‐18B). Medium was replaced daily for 3 days using 1.5 ml per 12 well. On day 2 after endoderm induction, medium was supplemented with Doxycycline at a final concentration of 0.8 μg/ml for samples transfected with bidirectional reporter systems and some of the control samples.

### Micropatterned plate preparation

The protocol employed has been reported elsewhere (Tewary *et al*, 2019; preprint: Kaul *et al*, 2020). Briefly, custom Nexterion‐D Borosilicate (Schott, D263) thin glass coverslips (dimensions 110 × 74 mm) were spin‐coated with Lipidure‐CM5206™ (Lipidure) (NOF America Corporation). Lipidure was reconstituted in 100% ethanol at 2.5 mg/ml. The coverslips were sterilized by covering their side with isopropanol alcohol and spinning them at 2,500 rpm for 30 s on the Laurell Spin Processor (Laurell Technologies Corporation, Model # WS‐650Mz‐23NPPB). This was followed by addition of 1.5 ml Lipidure solution on the sterilized side of the coverslip and spinning it at 2,500 rpm for another 30 s. To create micropatterns, the Lipidure‐coated side of the coverslip was exposed to deep UV for 20 min through a quartz photomask. The diameter of each micropattern was 1 mm. The coverslips were subsequently attached to 96‐well bottomless microtiter plates (VWR, Catalog # 82050‐714) using epoxy (Loctite, M‐31CL). Carboxyl groups on the photo‐activated regions were activated with 50 μl/well of 0.05 g/ml N‐(3‐Dimethylaminopropyl)‐N′‐ethylcarbodiimide hydrochloride (Sigma‐Aldrich, 03450) and N‐Hydrosuccinimide (Sigma‐Aldrich, 130672) solution for 20 min. The wells were then rinsed followed by the addition of 2% Geltrex. The plate was subsequently left on an orbital shaker overnight at 4°C.

### BMP4‐dependent differentiation of micropatterned RUES2 colonies

Micropatterned plates were thoroughly rinsed before the addition of cells. RUES2 cell suspension was created using TrypLE when the culture had reached 75–80% confluence. Cells were seeded at a density of 25,000 cells/wellwith the seeding medium containing the ROCK‐inhibitor (ROCK‐I) Y‐27632 (Reagents Direct, 53‐B85‐100). The seeding media consisted of DMEM, Knockout Serum Replacement (ThermoFisher, 10828028), Penicillin/Streptomycin (ThermoFisher, 15140122), 2‐Mercaptoethanol (ThermoFisher, 21985023), Glutamax (ThermoFisher, 35050061), Non‐Essential Amino Acids (ThermoFisher, 11140050), B27 without Retinoic Acid (ThermoFisher, 12587010), and bFGF (PeproTech, 100‐18B) supplemented at a concentration of 20 ng/ml. The plate was left at 37°C for 3 h after which the ROCK‐I containing seeding media was removed. The cells were then transfected as described above using NutriStem (100 μl/well) without ROCK‐I. The cells were incubated with DNA‐Transfection reagent mixture for 24 h until the micropatterns reached confluency. For the experiment where we tested exogenous BMP4, medium was replaced with NutriStem and left in Nutristem for 24 h until they reached confluency. At this point, differentiation was induced by replacing NutriStem with the N2B27 media supplemented with either BMP4 and NODAL or only NODAL (for transfected samples). NODAL (R&D Systems, Catalog # 3218‐ND) was added at 100 ng/ml. BMP4 (R&D Systems, Catalog # 314‐BP‐MTO) was used at a concentration of 50 ng/ml. For negative control, BMP4 was not added to the N2B27 media. The volume of media used for differentiation was 150 μl/well. Thirty‐six hours after induction of differentiation, the cell culture supernatant was collected for ELISA, three replicas were harvested for live flow cytometry, and remaining replicas (12 for each condition) were fixed and subsequently stained with DAPI and TBXT and measured by confocal microscopy.

### Confocal imaging of micropatterned colonies

Micropatterned samples were fixed with 4% paraformaldehyde and permeabilized using 100% cold methanol. Cells were exposed to the Human/Mouse Brachyury Affinity Purified Polyclonal Goat IgG antibody (R&D Systems, Catalog # AF2085) overnight at 4°C. The antibody was incubated in 2% FBS in HBSS. The samples were washed and immersed in the secondary antibody solution containing DAPI for 90 min at room temperature and subsequently rinsed.

Images were acquired using the Zeiss LSM 800 Confocal Microscope. We used magnification of 20× and obtained 5 z‐slices/colony. The RUES2 cell line contains endogenous tags for the markers SOX2 (mCitrine), SOX17 (tdTomato), and TBXT (mCerulean). We imaged SOX2 using the 488 nm diode laser (545 short pass filter) and SOX17 via the 561 nm diode laser (610 short‐pass filter). Due to the mCerulean signal being faint, we stained TBXT with the AlexaFluor 647 secondary antibody and imaged the signal with the 647 nm diode laser (655 long‐pass filter). Finally, the 647 nm diode laser was used to image the iRFP.

BMP4 concentration released into the media was quantified using the BMP‐4 Human ELISA Kit from (ThermoFisher, EHBMP4). Standards and protocols were done according to the instructions of the kit. The ELISA results were analyzed for absorbance at both 450 and 550 nm using the Tecan Infinite M Plex, and standard curve was calculated using CurveExpert software.

### Flow cytometry

Cells were incubated with TrypLE for 5 min at 37°C, mixed with equal volume of growth medium, dissociated to single cells by pipetting, and transferred to 96‐well v‐bottom plate for dead/live and/or antibody staining. Cells were spun down at 1,200 rpm (335G, Rotor Sx4750, Beckman Coulter Allegra X‐12R centrifuge) for 3 min and washed once with HF Buffer (HBSS, Gibco, 14175103) supplemented with 4% FBS. Cells that were not stained with antibodies (which were all transfections in 96‐well plates in pluripotency state) were incubated with a dead/live stain chosen based on compatibility with fluorescent reporter combination. Dead/live stains used were DAPI (Sigma‐Aldrich, D9542) incubated at a concentration of 0.1 μg/ml in HF for 3 min at room temperature or Zombie NIR™ (BioLegend, 423106) incubated in PBS at a 1:500 dilution for 15 min at room temperature. Cells have then been washed once with HF, suspended to single cells, and used for Flow Cytometry.

For definitive endoderm staining, half of a well from a 12‐well plate (2–4 × 10^5^ cells) were dissociated, washed once with HF, and co‐stained with conjugated PE‐CY7 anti‐human CD184 (BD, 560669) and APC anti‐human CD117 (BD, 550412) or with the corresponding isotype controls PE‐CY7 Mouse Ig2akappa Isotype (BD, 557907) and APC mouse IgG1kappa Isotype (BD, 555751). Each antibody was diluted to 1:200 and incubated in PBS containing LIVE/DEAD™ Fixable Violet Dead Cell Stain (diluted 1:1,000) for 30 min at room temperature. Cells were washed twice with HF buffer before using for Flow Cytometry.

For pluripotency markers staining, all cells from a 12‐well (4–6 × 10^5^ cells) were harvested and stained with LIVE/DEAD™ Fixable Violet Dead Cell Stain (diluted 1:1,000) for 30 min at room temperature. Cells were washed in HF buffer and incubated in 4% Formaldehyde diluted in HF Buffer for 15 min at room temperature. Cells were washed with HF and stored up to 1 week at 4°C if necessary. For permeabilization, cells were gently resuspended in 100% ice‐cold Methanol. After 2 min, twice the volume of HF was added to the cells and spun down at 1,200 rpm for 3 min. Then cells were resuspended with primary antibodies anti‐Oct‐3/4 (BD, 611203) and anti‐Sox2 (D6D9) (Cell Signaling Technologies, 3579S) diluted 1:100 in HF buffer and incubated for 30 min at room temperature. Cells were washed three times with HF buffer and incubated with secondary antibodies APC‐Cy7 goat anti‐rabbit IgG (BD, 611203) used at 1:200 dilution and AF647 Donkey anti‐mouse IgG (Thermo Fisher Scientific, A‐31571) used at 1:400 dilution and incubated for 15 min at room temperature. Negative background controls with only secondary antibodies were generated. Cells were washed once with HF and used for Flow Cytometry.

For both, pluripotency and endoderm antibody staining, single antibody‐stained controls, unstained controls with and without Dead/Live stain, and single Dead/Live stain controls using ArC™ Amine Reactive Compensation Beads (Thermo Fisher Scientific, A10628) were prepared for gating and cross‐talk evaluation.

Flow Cytometry was performed using BD LSR Fortessa Analyzer for samples shown in Figs 2, EV1, EV2, 3, EV3, EV4, 4, 5, 6. Micropatterned RUES2 cells (Fig 6) were measured using Beckman Coulter CytoFLEX LX flow cytometer. We used Calibration beads (BD, 642412) to ensure consistent machine performance and SPHERO RainBow Calibration particles (BD, 559123) to track and correct for consistency of PMT settings for each channel across different experiments.

### Analysis of flow cytometry data

All flow cytometry data were analyzed using FlowJo software. Single color controls were used to estimate the cross talk of each fluorophore into each channel. Compensation was performed using the FlowJo compensation matrix if necessary. The values in the various charts, labeled as relative expression units (rel.U.), were calculated as previously (Xie *et al*, 2011; Prochazka *et al*, 2014): (i) First single and live cells were gated based on their forward and side scatter readouts and the absence of dead/live marker. (ii) From this selection, cells that are positive in a given fluorophore were gated using fluorophore‐negative, transfection marker‐positive single‐color control such that 99.9% of cells in this single‐color control sample fall outside of the selected gate. (iii) For each positive cell population in a given channel, the mean value of the fluorescent intensity was calculated and multiplied by the frequency of the positive cells. This value was used as a measure for the total reporter signal in a sample. The total reporter signal of a circuit output was then normalized with the total signal of the reporter used as a transfection control to counterbalance possible transfection variation. The procedure can be summed in the following formula:

Reporter intensity of a sample in relative units (rel.U.) = [mean (Reporter in Reporter + cells) × Frequency (Reporter + cells)]/[mean (Transfection Marker in Transfection Marker + cells) × frequency (Transfection Marker + cells)].

Reporter intensity in absolute units (a.u.) as in Fig 6B and D = [mean (iRFP Reporter in Reporter + cells) × Frequency (iRFP Reporter + cells)].

Frequency of cells expressing a given marker as in Fig EV2C and E was calculated from (i) single cells and live cells were gated based on their forward and side scatter readouts and the absence of dead/live marker. (ii) From this selection, cells that are positive in a given marker were gated using control samples generated either with samples stained with only secondary antibodies (Fig EV2C) and isotype controls (Fig EV2E), respectively.

Data points in figures are shown as mean ± standard deviation (s.d.) of three or more independent biological samples. A two‐sided unpaired *t*‐test was performed on selected sample combinations as indicated in figures. Homoscedastic or heteroscedastic *t*‐tests were performed based on the results of an *F*‐test using a *P*‐value of 0.05. *P*‐values of the *t*‐test are indicated in figures as required.

Viability in Fig EV2D is calculated from the fraction of “live” cells in the Side and Forward Scatter multiplied with the fraction of cells that are negative for LIVE/DEAD™ Violet stain.

Correlation plots in Fig 3D were created with a simple linear regression model of the form *y* = *a* + *b* × *x*. The regression coefficient (*R* ^2^) was calculated to evaluate which circuit fits better with model predictions.

### Analysis of micropatterned colonies

CellProfiler was used to identify nuclear regions based on the intensities in the DAPI images. The intensities of Sox2, Sox17, and TBXT were then measured within these nuclear regions for each channel. Next, the single‐cell data of nuclear location and protein intensity were analyzed using ContextExplorer (CE; Ostblom *et al*, 2019) as described elsewhere (preprint: Kaul *et al*, 2020). Shortly, using the DBSCAN algorithm in CE, we clustered cells into colonies and assigned xy‐coordinates to each cell relative to the colony center. Cells were then grouped into hexagonal or annular bins to create the aggregation or line plots, respectively, and calculated the mean intensities and standard error of the markers within these bins.

To analyze the differences in germ‐layer marker expression (e.g., whether more cells exhibited SOX17 for a certain tuner or condition vs others), we calculated “area under the curve” (AUC) for the radial profiles of each marker. We first normalized the intensity values calculated via CE such that the highest radial intensity = 1. Next, we calculated the “area under the curve” (and the associated standard error) using the lowest radial intensity as baseline. Therefore, each condition/tuner had its unique baseline dependent on the marker expression. The “area under the curve” analysis were performed in GraphPad Prism 8.1.

## Author contributions

**Laura Prochazka:** Conceptualization; data curation; formal analysis; supervision; funding acquisition; validation; investigation; visualization; methodology; writing – original draft; project administration; writing – review and editing. **Yale S Michaels:** Conceptualization; formal analysis; methodology; writing – original draft; writing – review and editing. **Charles Lau:** Formal analysis; investigation. **Ross D Jones:** Formal analysis; investigation; writing – review and editing. **Mona Siu:** Formal analysis; investigation. **Ting Yin:** Formal analysis; investigation. **Diana Wu:** Formal analysis; investigation. **Esther Jang:** Formal analysis; investigation. **Mercedes Vázquez‐Cantú:** Formal analysis; investigation; writing – review and editing. **Penney M Gilbert:** Resources; writing – review and editing. **Himanshu Kaul:** Formal analysis; funding acquisition; investigation; writing – review and editing. **Yaakov Benenson:** Resources; supervision; funding acquisition; writing – review and editing. **Peter W Zandstra:** Conceptualization; resources; supervision; funding acquisition; methodology; writing – review and editing.

## Disclosure and competing interests statement

The authors declare that they have no conflict of interest but want to disclose that PWZ is scientific founder and consultant of Notch Therapeutics and LP is a scientist at Notch Therapeutics. YB is founder and shareholder of Pattern Biosciences.

## Supporting information




Appendix

Expanded View Figures PDF

Dataset EV1

Source Data for Expanded View

PDF+

Source Data for Figure 2

Source Data for Figure 3

Source Data for Figure 4

Source Data for Figure 5

Source Data for Figure 6

## Data Availability

The vector maps of all plasmids generated in this study as listed in Appendix Table S2 are available on Addgene. The code for automated miRNA identification with modified pre‐processing pipeline is available on GitHub: https://github.com/jwon0408/SynNetModified. Source data for Figs 2, EV1, EV2, 3, EV3, EV4, 4, 5, 6 are provided. Additional raw data can be shared upon request.
