# Supplementary material for: Synthetic gene circuits for cell state detection and protein tuning in human pluripotent stem cells
Source: Mol Syst Biol. 2022 Nov 11;18(11):e10886. doi: 10.15252/msb.202110886 (PMC9650275; doi:10.15252/msb.202110886)
Supplement: Supplementary file 1 — Appendix [file MSB-18-e10886-s008.pdf]

Appendix Information for

Synthetic gene circuits for cell state detection and protein tuning in human pluripotent stem cells

Laura Prochazka[1,2], Yale S. Michaels[3,4], Charles Lau[1,2,3,4], Ross D. Jones[3,4,] Mona Siu[3,4], Ting Yin[1,2], Diana Wu[1,2], Esther Jang[1,2], Mercedes Vázquez-Cantú[1,2,5], Penney M. Gilbert[1,2,6], Himanshu Kaul[3,4,7], Yaakov Benenson[5], Peter W. Zandstra[3,4*]

Appendix Figure S1: Parameter screens related to Figure3A-B

Appendix Figure S2: Supplementary data related to Figure 4

Appendix Table S1: misFITs library details

Appendix Table S2: Plasmid list

Appendix Figure S1

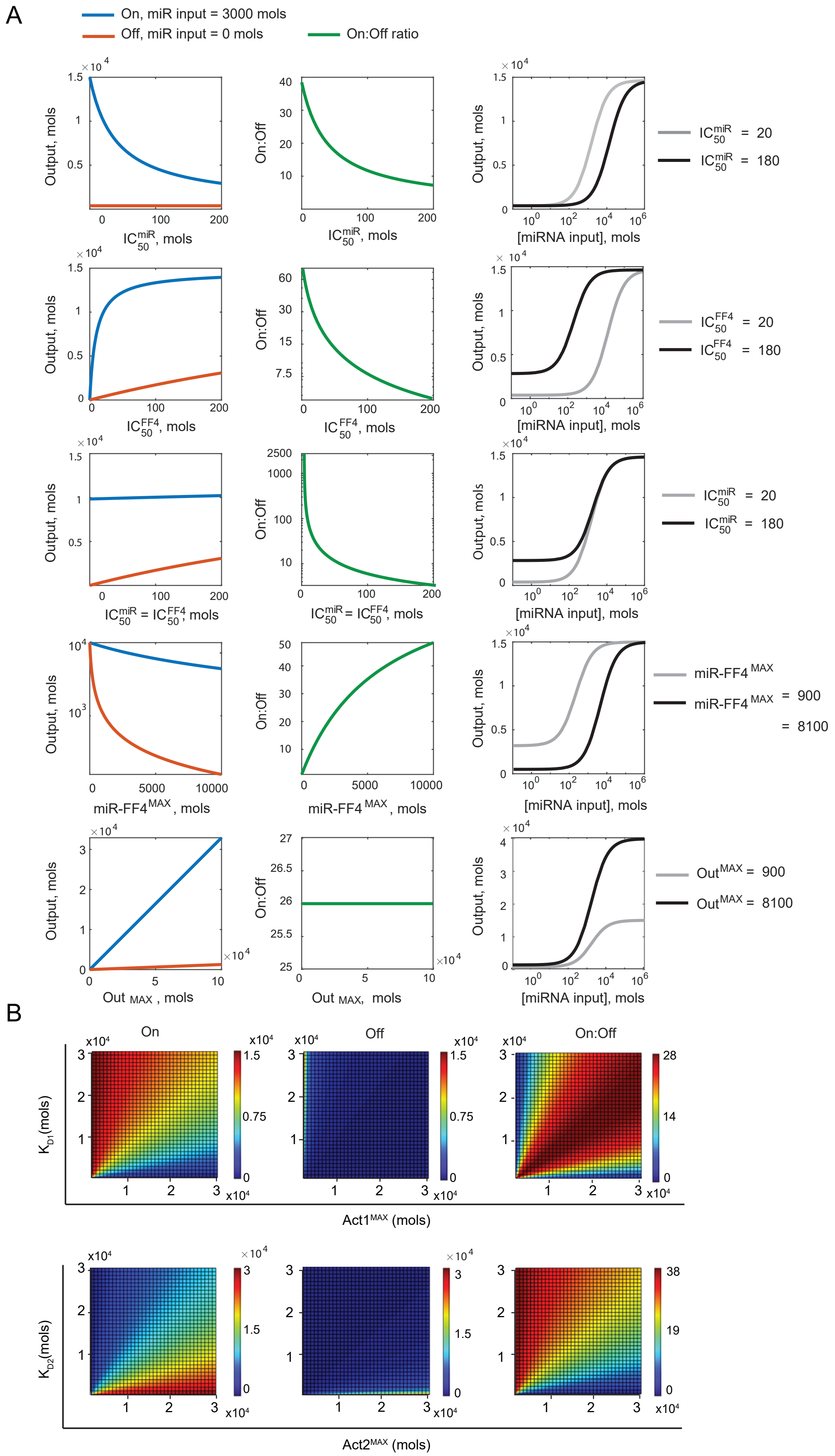

Appendix Figure S1 A) Single parameter screening. Shown are dose response curves of individual parameters (IC_{50}^{miR} , $IC_{50}^{FF4,}$ miR-FF4^{MAX} and Out^{MAX}) for On and Off state (left), On / Off ratio (middle) and input – output transfer function for each parameter at low and high miRNA levels (right). **B)** Combinatorial screening of KD and Act^{MAX} for Act1 (top) and Act2 (bottom). Shown are calculated output levels in On state (left), Off state (middle) and On / Off ratio. Related to Figure 3A and 3B.

Appendix Figure S2

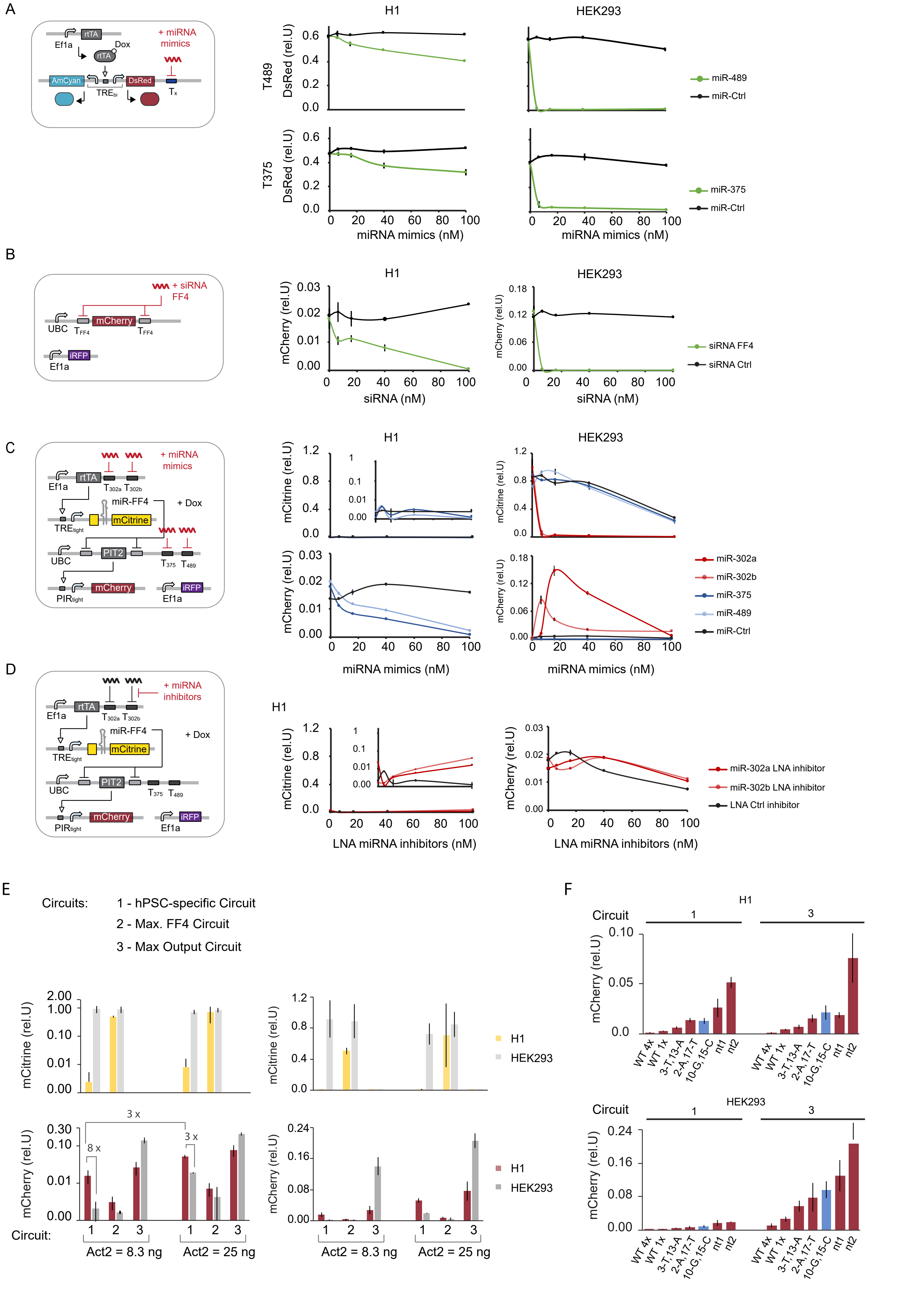

Appendix Figure S2 A) Repression of bi-directional reporter by miR-375 and miR-489 mimics. DsRed is either targeted with four fully complementary miR-489 (top) or miR-375 (bottom) targets sites. Charts show normalized DsRed expression in response to increasing amounts of miRNA mimics in H1 and HEK293 as indicated. **B)** Repression strength of siRNA FF4 on UBC driven mCherry reporter flanked with FF4 targets sites (same design as Act2 expressing constructs in Figure 3 and 4). Chart shows normalized mCherry expression in response to increasing amounts of siRNA FF4 **C)** hPSC-specific circuit (circuit 1) in response to miRNA mimic administration. Charts show mCitrine (top) and mCherry (bottom) expression in response to increasing concentrations of miRNA mimics in H1 (left) and HEK293 (right). Related to Figure 4D. **D)** hPSC-specific circuit (circuit 1) in response to LNA inhibitors. Charts show mCitrine (top) and mCherry (bottom) expression in response to increasing amounts of miR-302a and miR-302b LNA inhibitors in H1 (left) and HEK293 (right). **E)** Comparison of circuit performance using 8.3 and 25 ng of Act2-expressing plasmid, corresponding to a 0.1:1:0.3:1 and a 0.1:1:1:1 molar ratio between circuit plasmids, respectively. Bar charts show FF4 / mCitrine expression (top) and output / mCherry expression (bottom) in log scale (left) and linear scale (right). Related to Figure 4C. **F)** Bar chart showing fine tuning of output using miSFITs library in H1 and HEK293. Related to Figure 4E. All charts show mean ± s.d. of at least three biological replicates.

Appendix Table S1: miSFITs T17 variant target sequences

Target _ID	Base change(s)	Target sequence 5' – 3'	Oligo sequence (incl. cloning overhangs) 5'-3'
V2	10-G, 15-C	ctacctgcaGtgtaCgcactttg	**ggccgcaaa**gaagaccaGCTTGCTACACGGGTCAACATACTAACACGCAActacctgc aGtgtaCgcactttgTCATCCTCCATACGCAGCTATGCggtgAGGGTActgtcttcagG
V4	2-A, 17-T	cAacctgcactgtaagTactttg	**ggccgcaaa**gaagaccaGCTTGCTACACGGGTCAACATACTAACACGCAAcAacctg cactgtaagTactttgTCATCCTCCATACGCAGCTATGCggtgAGGGTActgtcttcagG
V5	9-G	ctacctgcGctgtaagcactttg	**ggccgcaaa**gaagaccaGCTTGCTACACGGGTCAACATACTAACACGCAActacctgc Gctgtaagcactttg TCATCCTCCATACGCAGCTATGCggtgAGGGTActgtcttcagG
V8	3-T; 13-A	ctTcctgcactgAaagcactttg	**ggccgcaaa**gaagaccaGCTTGCTACACGGGTCAACATACTAACACGCAActTcctgc actgAaagcactttgTCATCCTCCATACGCAGCTATGCggtgAGGGTActgtcttcagG
V11	9-G, 15-C	ctacctgcGctgtaCgcactttg	**ggccgcaaa**gaagaccaGCTTGCTACACGGGTCAACATACTAACACGCAActacctgc GctgtaCgcactttgTCATCCTCCATACGCAGCTATGCggtgAGGGTActgtcttcagG
V12	12-C, 21-G	ctacctgcactCtaagcactGtg	**ggccgcaaa**gaagaccaGCTTGCTACACGGGTCAACATACTAACACGCAActacctgc actCtaagcactGtgTCATCCTCCATACGCAGCTATGCggtgAGGGTActgtcttcagG
V15	3-C	ctCcctgcactgtaagcactttg	**ggccgcaaa**gaagaccaGCTTGCTACACGGGTCAACATACTAACACGCAActCcctgc actgtaagcactttgTCATCCTCCATACGCAGCTATGCggtgAGGGTActgtcttcagG
WT	None	Ctacctgcactgtaagcactttg	**gccgcaaa**gaagaccaGCTTGCTACACGGGTCAACATACTAACACGCAActacctgca ctgtaagcactttgTCATCCTCCATACGCAGCTATGCggtgAGGGTActgtcttcagG

Appendix Table S2: Plasmid list

Bi-directional reporters

Name	Description	Backbone	Source of insert	Deposit on Addgene
pLP110	AmCyan-TRE-DsRed-T708-5p	pZ073	ssDNA, Seq from miRBase	Yes
pLP111	AmCyan-TRE-DsRed-T489-3p	pZ073	ssDNA, Seq from miRBase	Yes
pLP112	AmCyan-TRE-DsRed-T375-3p	pZ073	ssDNA, Seq from miRBase	Yes
pLP113	AmCyan-TRE-DsRed-T302a-3p	pZ073	ssDNA, Seq from miRBase	Yes
pLP114	AmCyan-TRE-DsRed-T302b-3p	pZ073	ssDNA, Seq from miRBase	Yes
pLP115	AmCyan-TRE-DsRed	pZ073	Self-ligation without insert	Yes
pLP110	AmCyan-TRE-DsRed-T708-5p	pZ073	ssDNA, Seq from miRBase	Yes
pLP111	AmCyan-TRE-DsRed-T489-3p	pZ073	ssDNA, Seq from miRBase	Yes
pLP118	AmCyan-TRE-DsRed-TFF3	pZ073	ssDNA, Seq from (Leisner et al., 2010)	Yes
pLP119	AmCyan-TRE-DsRed-TFF6	pZ073	ssDNA, Seq from (Leisner et al., 2010)	Yes
pLP176	AmCyan-TRE-DsRed-T17 WT1x	pZ073	ssDNA, Seq from miRBase	Yes
pLP177	AmCyan-TRE-DsRed-T17 V4	pZ073	ssDNA, Seq from (Michaels et al., 2019)	Yes
pLP178	AmCyan-TRE-DsRed-T17 V2	pZ073	ssDNA, Seq from (Michaels et al., 2019)	Yes
pLP179	AmCyan-TRE-DsRed-T17 V5	pZ073	ssDNA, Seq from (Michaels et al., 2019)	Yes
pLP180	AmCyan-TRE-DsRed-T17 V8	pZ073	ssDNA, Seq from (Michaels et al., 2019)	Yes
pLP181	AmCyan-TRE-DsRed-T17 V7	pZ073	ssDNA, Seq from (Michaels et al., 2019)	Yes
pLP182	AmCyan-TRE-DsRed-T17 V11	pZ073	ssDNA, Seq from (Michaels et al., 2019)	Yes
pLP183	AmCyan-TRE-DsRed-T17 V12	pZ073	ssDNA, Seq from (Michaels et al., 2019)	Yes
pLP188	AmCyan-TRE-DsRed-T17 WT	pZ073	ssDNA, Seq from (Michaels et al., 2019)	Yes
pLP189	AmCyan-TRE-DsRed-T17 V10	pZ073	ssDNA, Seq from (Michaels et al., 2019)	Yes
pLP190	AmCyan-TRE-DsRed-V13	pZ073	ssDNA, Seq from (Michaels et al., 2019)	Yes
pLP191	AmCyan-TRE-DsRed-neg Ctrl	pZ073	ssDNA, Seq from (Michaels et al., 2019)	Yes
pLP192	AmCyan-TRE-DsRed-V15	pZ073	ssDNA, Seq from (Michaels et al., 2019)	Yes
pLP193	AmCyan-TRE-DsRed-T17 V9	pZ073	ssDNA, Seq from (Michaels et al., 2019)	Yes

MoClo Level 0 backbones (to restore Kozak sequence)

Name	Description	Backbone	Deposit on Addgene
pLP301	5’ Kozak pICH41258	pICH41258	Yes
pLP302	5’ Kozak pAGM1287	pAGM1287	Yes
pLP303	5’Kozak pICH41308	pICH41308	Yes
pLP220	3’ Kozak pAGM1276	pAGM1276	Yes
pLP221	3’ Kozak pICH41246	pICH41246	Yes
pLP222	3’ Kozak pICH41295	pICH41295	Yes

MoClo level 0 library

Name	Description	Level 0 Backbone	Source of insert	Deposit on Addgene
pLP133	UBC	pICH41233	dsDNA, Seq from (Schreiber et al., 2016), seq modified to make compatible for MoClo cloning	Yes
pLP134	PIT2	pICH41258	dsDNA, Seq from (Schreiber et al., 2016), codon modified for compatibility with MoClo cloning, originates from (W. Weber, Kramer, et al., 2002)	Yes
pLP135	miR302a	pAGM1263	PCR from pLP113, Seq from miRBase	Yes
pLP136	miR302a	pAGM1299	PCR from pLP113, Seq from miRBase	Yes
pLP137	miR302b	pAGM1276	PCR from pLP114, Seq from miRBase	Yes
pLP138	miR302b	pAGM1301	PCR from pLP114. Seq from miRBase	Yes
pLP139	SV40 polyA	pICH41276	PCR from pJS23 (Schreiber et al., 2016)	Yes
pLP140	5’UTR Spacer	pICH41246	ssDNA	Yes
pLP142	T375 4x	pAGM1299	PCR from pLP112, Seq from miRBase	Yes

pLP143	T489 4x	pAGM1301	PCR from pLP111, Seq from miRBase	Yes
pLP144	T708 4x	pICH53388	PCR from pLP110, Seq from miRBase	Yes
pLP145	PolyA	pICH53399	PCR from pLP103, polyA originates from pJS32 (Schreiber et al., 2016)	Yes
pLP146	UBC	pICH41295	PCR from pLP133, Seq originates from(Schreiber et al., 2016)	Yes
pLP147	TFF6 4x	pAGM1299	PCR from pLP119, Seq originates from (Leisner et al., 2010)	Yes
pLP148	TFF6 4x	pAGM1301	PCR from pLP119, Seq originates from (Leisner et al., 2010)	Yes
pLP149	TFF6 4x	pICH53388	PCR from pLP119, Seq originates from (Leisner et al., 2010)	Yes
pLP164	TFF3	pAGM1301	PCR from pLP118, Seq originates from (Leisner et al., 2010)	Yes
pLP194	TFF3 4x	pAGM1287	ssDNA, Seq originates from (Leisner et al., 2010)	Yes
pLP195	TFF6 4x	pAGM1287	ssDNA, Seq originates from (Leisner et al., 2010)	Yes
pLP196	T375 4x	pAGM1287	ssDNA, Seq from miRBase	Yes
pLP197	sBFP	pAGM1301	PCR from pLP088/ pCS187 (Stelzer & Benenson, 2020)	Yes
pLP198	mCherry	pAGM1301	PCR from pLP027/pKH026 (Prochazka et al., 2014)	Yes
pLP199	iRFP	pAGM1301	PCR from pLP029/pCS184 (Prochazka et al., 2014)	Yes
pLP206	tTA	pICH41258	PCR from Addgene plasmid #24415, codon modified for compatibility with MoClo cloning	Yes
pLP210	ET1	pICH41258	dsDNA, seq from (Prochazka et al., 2014), originates from (W. Weber, Kramer, et al., 2002)	Yes
pLP223	ETRtight	pAGM41295	dsDNA, seq from (Prochazka et al., 2014), ETR binding sites originate from (W. Weber, Fux, et al., 2002)	Yes
pLP233	TFF4 3x	pICH41246	dsDNA, Seq from (Xie et al., 2011)	Yes
pLP235	TFF4 3x	pICH41264	dsDNA, Seq from (Xie et al., 2011)	Yes
pLP237	tTAmut	pICH41258	dsDNA, Seq from (Roney et al., 2016)	Yes
pLP238	miR375 miR489 miR708	pICH53388	PCR from pLP169	Yes
pLP240	TRE3G	pICH41233	PCR from pLP089, Seq originally from Addgene #61474	Yes
pLP242	5'UTR Spacer (restores Kozak)	pLP221	dsDNA	Yes
pLP243	5'UTR Spacer	pICH41246	dsDNA	Yes
pLP245	Citrine FF4	pICH41258 with Kozak OH	PCR from pJS41 (Schreiber et al., 2016)	Yes
pLP247	3'UTR spacer	pICH41264	ssDNA	Yes
pLP248	mCherry	pICH41258	PCR from pLP027 (Prochazka et al., 2014)	Yes
pLP251	PIRtight	pICH41233	PCR from (Prochazka et al., 2014), PIR binding site originates from (W. Weber, Kramer, et al., 2002)	Yes
pLP252	ETRtight	pICH41233	PCR from (Prochazka et al., 2014), ETR binding site originally from (W. Weber et al., 2009)	Yes
pLP253	5'UTR spacer	pICH41264	ssDNA, Seq fom pLP027 (Prochazka et al., 2014)	Yes
pLP254	rtTA	pICH41258	PCR from pLP059 / Addgene #61472	Yes
pLP255	Ef1a	pICH41233	PCR from pLP027 (Prochazka et al., 2014)	Yes
pLP257	T375 4x	pAGM1299	ssDNA, Seq from miRBase	Yes
pLP258	T489 4x	pAGM1301	ssDNA, Seq from miRBase	Yes
pLP259	T708 4x	pICH53388	ssDNA, Seq from miRBase	Yes
pLP305	TFF3 4x	pAGM1299	ssDNA, Seq from (Leisner et al., 2010)	Yes
pLP306	TFF6 4x	pAGM1301	ssDNA, Seq from (Leisner et al., 2010)	Yes

pLP314	T302a 4x	pICH41264	ssDNA, Seq from miRBase	Yes
pLP315	TFF3 4x	pICH41264	ssDNA, Seq from (Leisner et al., 2010)	Yes
pLP316	TFF4 3x	pAGM1299	ssDNA, Seq from (Xie et al., 2011)	Yes
pLP317	T375-3p	pAGM1301	ssDNA, Seq from miRBase	Yes
pLP318	T489 4x	pICH53388	ssDNA, Seq from miRBase	Yes
pLP319	TFF3 4x	pAGM1301	ssDNA, Seq from (Leisner et al., 2010)	Yes
pLP320	TFF6 4x	pICH53388	ssDNA, Seq from (Leisner et al., 2010)	Yes
pLP332	Spacer	pICH41264	ssDNA	Yes
pLP333	Spacer	pICH53388	ssDNA	Yes
pLP334	TRE3G	pLP222	PCR from pLP089, Seq originates from Addgene #61474	Yes
pLP337	BMP4	pLP301	dsDNA, Seq from USCS, hg38_knownGene_ENST00000245451.9 range=chr14:53950032-53952222, CDS only, then codon optimized with IDT tool	Yes
pLP364	PIRtight	pLP222	PCR from pLP269, Seq from (E. Weber et al., 2011), binding site originates from (W. Weber, Kramer, et al., 2002)	Yes
pLP365	mCherry	pLP301	PCR from pLP027 (Prochazka et al., 2014)	Yes
pLP366	sBFP2	pLP301	PCR from pLP027 (Prochazka et al., 2014)	Yes
pLP367	T17 4x	pICH53388	ssDNA, Seq from miRBase	Yes
pLP368	T17 V10	pICH53388	ssDNA, Seq from (Michaels et al., 2019)	Yes
pLP349	T17 V2	pICH53388	ssDNA, Seq from (Michaels et al., 2019)	Yes
pLP350	T17 V4	pICH53388	ssDNA, Seq from (Michaels et al., 2019)	Yes
pLP351	T17 V8	pICH53388	ssDNA, Seq from (Michaels et al., 2019)	Yes
pLP352	T17 V1	pICH53388	ssDNA, Seq from (Michaels et al., 2019)	Yes
pLP353	T17 V7	pICH53388	ssDNA, Seq from (Michaels et al., 2019)	Yes
pLP354	T17 V14	pICH53388	ssDNA, Seq from (Michaels et al., 2019)	Yes
pLP355	neg ctrl	pICH53388	ssDNA, Seq from (Michaels et al., 2019)	Yes
pLP356	T17 V12	pICH53388	ssDNA, Seq from (Michaels et al., 2019)	Yes
pLP357	T17 V9	pICH53388	ssDNA, Seq from (Michaels et al., 2019)	Yes
pLP358	T17 V5	pICH53388	ssDNA, Seq from (Michaels et al., 2019)	Yes
pLP359	T17 V11	pICH53388	ssDNA, Seq from (Michaels et al., 2019)	Yes

MoClo level1 library (functional plasmids)

Name	Description	Level 1 Backbone	Deposit to Addgene
pLP264	UBC-TFF4-ET1-TFF4-T375-T489-T708-polyA	pICH47802	Yes
pLP265	UBC-TFF4-PIT2-TFF4-T375-T489-T708-polyA	pICH47802	Yes
pLP266	UBC-TFF4-tTA-TFF4-T375-T489-T708-polyA	pICH47802	Yes
pLP267	UBC-TFF4-tTAmut-TFF4-T375-T489-T708-polyA	pICH47802	Yes
pLP268	ETRtight-mCherry-polyA	pICH47742	Yes
pLP269	PIRtight-mCherry-polyA	pICH47742	Yes
pLP271	TRE3G-mCherry-polyA	pICH47742	Yes
pLP275	TRE3G-Citrine FF4-polyA	pICH47742	Yes
pLP292	Ef1a-spacer-rtTA-TFF6-TFF3-polyA	pICH47802	Yes
pLP293	Ef1a-spacer-rtTA-T302a-T302b-polyA	pICH47802	Yes
pLP294	Ef1a-spacer-PIT2-TFF6-TFF3-polyA	pICH47802	Yes
pLP295	Ef1a-spacer-PIT2-T302a-T302b-polyA	pICH47802	Yes

pLP299	PIRtight-mCitrine FF4-polyA	pICH47742	Yes
pLP324	Ef1a-spacer-rtTA-T302a-polyA make map	pICH47802	Yes
pLP325	Ef1a-spacer-rtTA-FF3-polyA make map	pICH47802	Yes
pLP327	UBC-TFF4-PIT2-TFF4 T375 T489 make map	pICH47742	Yes
pLP342	TRE3G-iRFP-spacer-polyA	pICH47742	Yes
pLP343	TRE3G-BMP4-spacer-polyA	pICH47742	Yes
pLP360	UBC-TFF4-PIT2-TFF4 TFF3 TFF6 make map	pICH47742	Yes
pLP370	PIRtight_mCherry_spacer	pICH47802	Yes
pLP371	PIRtight_sBFP_spacer_polyA	pICH47822	Yes
pCL039	PIRtight-Cherry-Spacer-T17 WT 1x-polyA	pICH47802	Yes
pCL040	PIRtight-Cherry-Spacer-T17 V2-polyA	pICH47802	Yes
pCL041	PIRtight-Cherry-Spacer-T17 V4-polyA	pICH47802	Yes
pCL042	PIRtight-Cherry-Spacer-T17 V8-polyA	pICH47802	Yes
pCL043	PIRtight-Cherry-Spacer-T17 4x-polyA	pICH47802	Yes
pCL044	PIRtight-sBFP-Spacer-T17 WT 1x-polyA	pICH47822	Yes
pCL045	PIRtight-sBFP-Spacer-T17 V2-polyA	pICH47822	Yes
pCL046	PIRtight-sBFP-Spacer-T17 V4-polyA	pICH47822	Yes
pCL047	PIRtight-sBFP-Spacer-T17 V8-polyA	pICH47822	Yes
pCL048	PIRtight-sBFP-Spacer-T17 4x-polyA	pICH47822	Yes
pCL056	TRE3G-BMP4-Spacer-T17 WT 1x-polyA	pICH47742	Yes
pCL057	TRE3G-BMP4-Spacer-T17 V2-polyA	pICH47742	Yes
pCL058	TRE3G-BMP4-Spacer-T17 V4-polyA	pICH47742	Yes
pCL059	TRE3G-BMP4-Spacer-T17 V8-polyA	pICH47742	Yes
pCL060	TRE3G-BMP4-Spacer-T17 4x-polyA	pICH47742	Yes
pCL112	EF1a-spacer-tTA-T375 4x-T489 4x	pICH47802	Yes
pCL113	EF1a-spacer-tTA-T375 4x-T375 4x	pICH47802	Yes
pCL114	EF1a-spacer-tTA-T302a 4x-T302b 4x	pICH47802	Yes
pCL115	EF1a-spacer-tTA-TFF3 4x-TFF6	pICH47802	Yes
pCL116	EF1a-spacer-PIT2-T375 4x-T489	pICH47802	Yes

Other plasmids used in this study (not cloned)

**Name**	**Description**	**Source**	**Deposit to Addgene**
pLP026 / pKH025	Ef1a-mCitrine	(Prochazka et al., 2014)	No
pLP027 / pKH026	Ef1a-mCherry	(Prochazka et al., 2014)	No
pLP029 / pCS184	Ef1a-iRFP	(Prochazka et al., 2014)	No
pLP088 / pCS187	Ef1a-sBFP2	(Stelzer & Benenson, 2020)	No
pLP076 / pJS37	UBC-TFF4-mCherry-TFF4-polyA	(Schreiber et al., 2016)	No
pLP085 / pZ145	AmCyan-TRE-DsRed-T17	(Xie et al., 2011)	No

References

Leisner, M., Bleris, L., Lohmueller, J., Xie, Z., & Benenson, Y. (2010). Rationally designed logic integration of regulatory signals in mammalian cells. *Nature Nanotechnology*, *5*(9). https://doi.org/10.1038/nnano.2010.135

Michaels, Y. S., Barnkob, M. B., Barbosa, H., Baeumler, T. A., Thompson, M. K., Andre, V., Colin-York, H., Fritzsche, M., Gileadi, U., Sheppard, H. M., Knapp, D. J. H. F., Milne, T. A., Cerundolo, V., & Fulga, T. A. (2019). Precise tuning of gene expression levels in mammalian cells. *Nature Communications*, *10*(1). https://doi.org/10.1038/s41467-019-08777-y

Prochazka, L., Angelici, B., Haefliger, B., & Benenson, Y. (2014). Highly modular bow-tie gene circuits with programmable dynamic behaviour. *Nature Communications*, *5*. https://doi.org/10.1038/ncomms5729

Roney, I. J., Rudner, A. D., Couture, J. F., & Kærn, M. (2016). Improvement of the reverse tetracycline transactivator by single amino acid substitutions that reduce leaky target gene expression to undetectable levels. *Scientific Reports*, *6*. https://doi.org/10.1038/srep27697

Schreiber, J., Arter, M., Lapique, N., Haefliger, B., & Benenson, Y. (2016). Model-guided combinatorial optimization of complex synthetic gene networks. *Molecular Systems Biology*, *12*(12). https://doi.org/10.15252/msb.20167265

Stelzer, C., & Benenson, Y. (2020). Precise determination of input-output mapping for multimodal gene circuits using data from transient transfection. *PLoS Computational Biology*, *16*(11). https://doi.org/10.1371/journal.pcbi.1008389

Weber, E., Engler, C., Gruetzner, R., Werner, S., & Marillonnet, S. (2011). A modular cloning system for standardized assembly of multigene constructs. *PLoS ONE*, *6*(2). https://doi.org/10.1371/journal.pone.0016765

Weber, W., Fux, C., Daoud-El Baba, M., Keller, B., Weber, C. C., Kramer, B. P., Heinzen, C., Aubel, D., Bailey, J. E., & Fussenegger, M. (2002). Macrolide-based transgene control in mammalian cells and mice. *Nature Biotechnology*, *20*(9). https://doi.org/10.1038/nbt731

Weber, W., Kramer, B. P., Fux, C., Keller, B., & Fussenegger, M. (2002). Novel promoter/transactivator configurations for macrolide- and streptogramin-responsive transgene expression in mammalian cells. *Journal of Gene Medicine*, *4*(6). https://doi.org/10.1002/jgm.314

Weber, W., Schuetz, M., Dénervaud, N., & Fussenegger, M. (2009). A synthetic metabolite-based mammalian inter-cell signaling system. *Molecular BioSystems*, *5*(7). https://doi.org/10.1039/b902070p

Xie, Z., Wroblewska, L., Prochazka, L., Weiss, R., & Benenson, Y. (2011). Multi-input RNAi-based logic circuit for identification of specific cancer cells. *Science*, *333*(6047). https://doi.org/10.1126/science.1205527
