# Supplementary material for: Synthetic gene circuits for cell state detection and protein tuning in human pluripotent stem cells
Source: Mol Syst Biol. 2022 Nov 11;18(11):e10886. doi: 10.15252/msb.202110886 (PMC9650275; doi:10.15252/msb.202110886)
Supplement: Supplementary file 2 — Expanded View Figures PDF [file MSB-18-e10886-s007.pdf]

Expanded View Figures

Figure EV1. Summary of computationally identified circuits showing input miRNAs and predicted circuit output levels using different maximum input constraints.

Shown are identified circuits when ten or five maximum input number are used with or without pruning using miRNA data sets from Bar *et al* (2008a), Data ref: Bar *et al* (2008b), Lipchina *et al* (2011a), Data ref: Lipchina *et al* (2011b), Data ref: Fogel *et al* (2015a) and Fogel *et al* (2015b). Circuit identification using maximum of three input constraint is depicted in Fig 2A. Expression levels of identified miRNAs inputs are given as fold change over the pre-set input abundance threshold (t) of the total miRNA pool (where $t = 1\%$) (left). Calculated circuit output levels are given as mol/cell (middle) and logic connectivity of the identified miRNA is depicted (right). miRNA expression data and nomenclature can be found in Dataset EV1. Raw output data and constraint files of the algorithm in Source Data file. Related to Fig 2A.

Source data are available online for this figure.

© 2022 The Authors

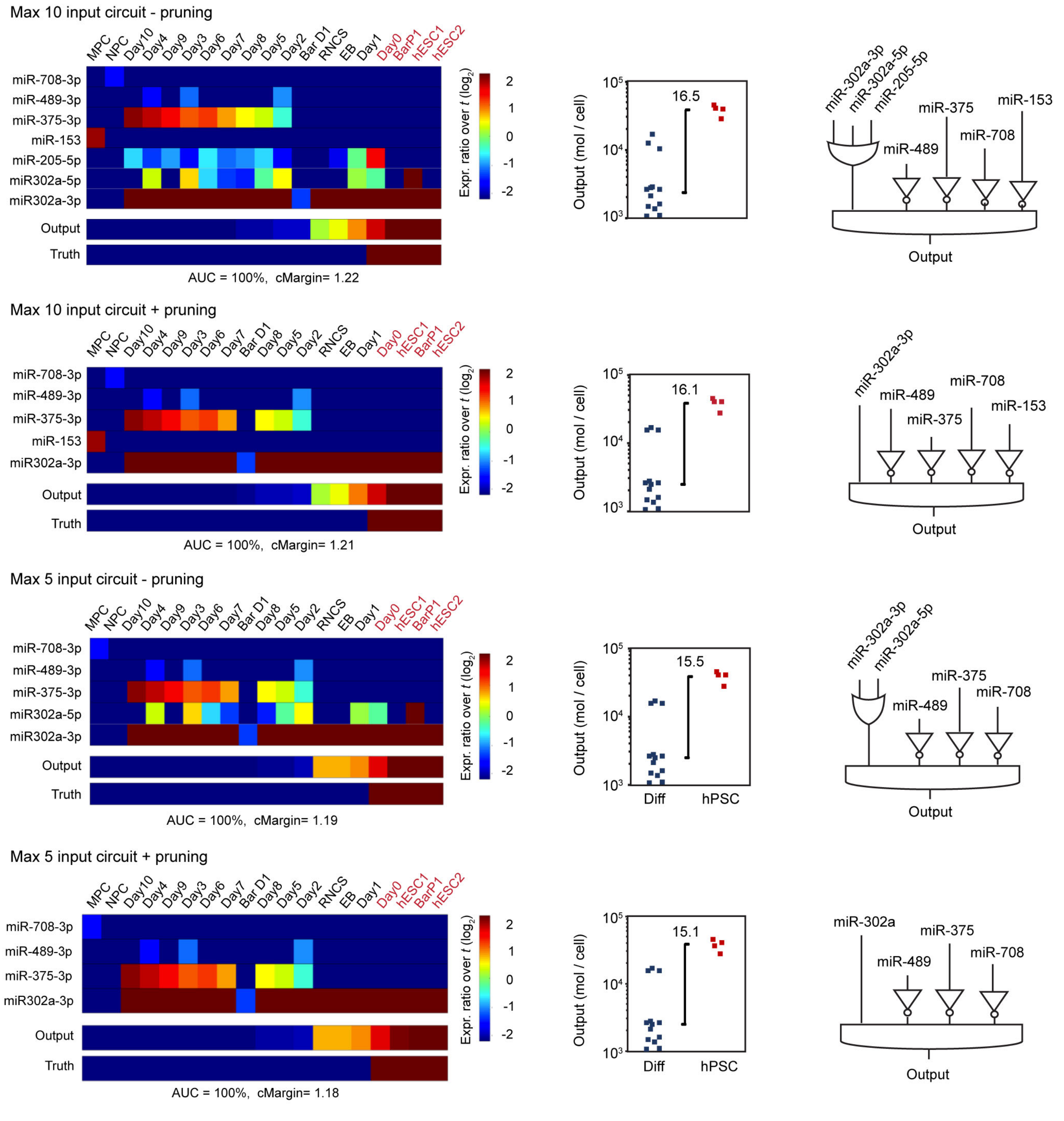

Figure EV1.

 © 2022 The Authors

Figure EV2. Characterization of computationally identified miRNA-sensors on pluripotency marker expression, viability and endoderm differentiation in hPSC.

A Illustration of bidirectional miRNA sensor system.

B Bar chart showing relative DsRed expression of sensors containing the indicated miRNA target sites as four fully complementary repeats. The control vector (n.t.) does not contain any target sites. Two-tailed unpaired *t*-tests were performed to compare T708, T489 and T375 to TFF3, TFF6 or to n.t. for each cell line. *P*-values < 0.01 were considered very significant (**), *P*-value $= 0.1$ (*) was considered not significant. The bar groups samples with identical *P*-values. Each bar chart corresponds to mean $\pm$ s.d. from at least three biological replicates. Related to Fig 2C.

C–E Bar chart left showing the fraction of $Oc4^{+}$ Sox^{+} double-positive pluripotent cells (C), viability (D) and $CD184^{+}$ and $CD117^{+}$ double-positive cells marking definitive endoderm (DE) (D) for each miRNA sensor (+) and the untransfected samples (–) in different conditions as indicated. Bars show mean $\pm$ s.d. of at least three biological replicates. Individual samples are indicated as black dots. Box plot on the right shows all samples transfected with miRNA sensors (+) and all untransfected samples (–) in H1 and HES-2. Box indicates first to third quartile range, horizontal line in box shows median, whiskers indicate the min-max range and dots show inner data points (excluding min and max data point). Two-tailed unpaired *t*-tests were performed to compare the two sample groups. *P*-values < 0.01 were considered very significant (**), < 0.05 significant (*) and > 0.5 not significant.

F misFITS library testing in hPSC. Histograms of the flow cytometry data shown in Fig 2D. Fluorescent intensity of DsRed (left) and DsRed normalized with internal AmCyan control (DsRed/AmCyan, right) shown for H1 and HES-2 as indicated.

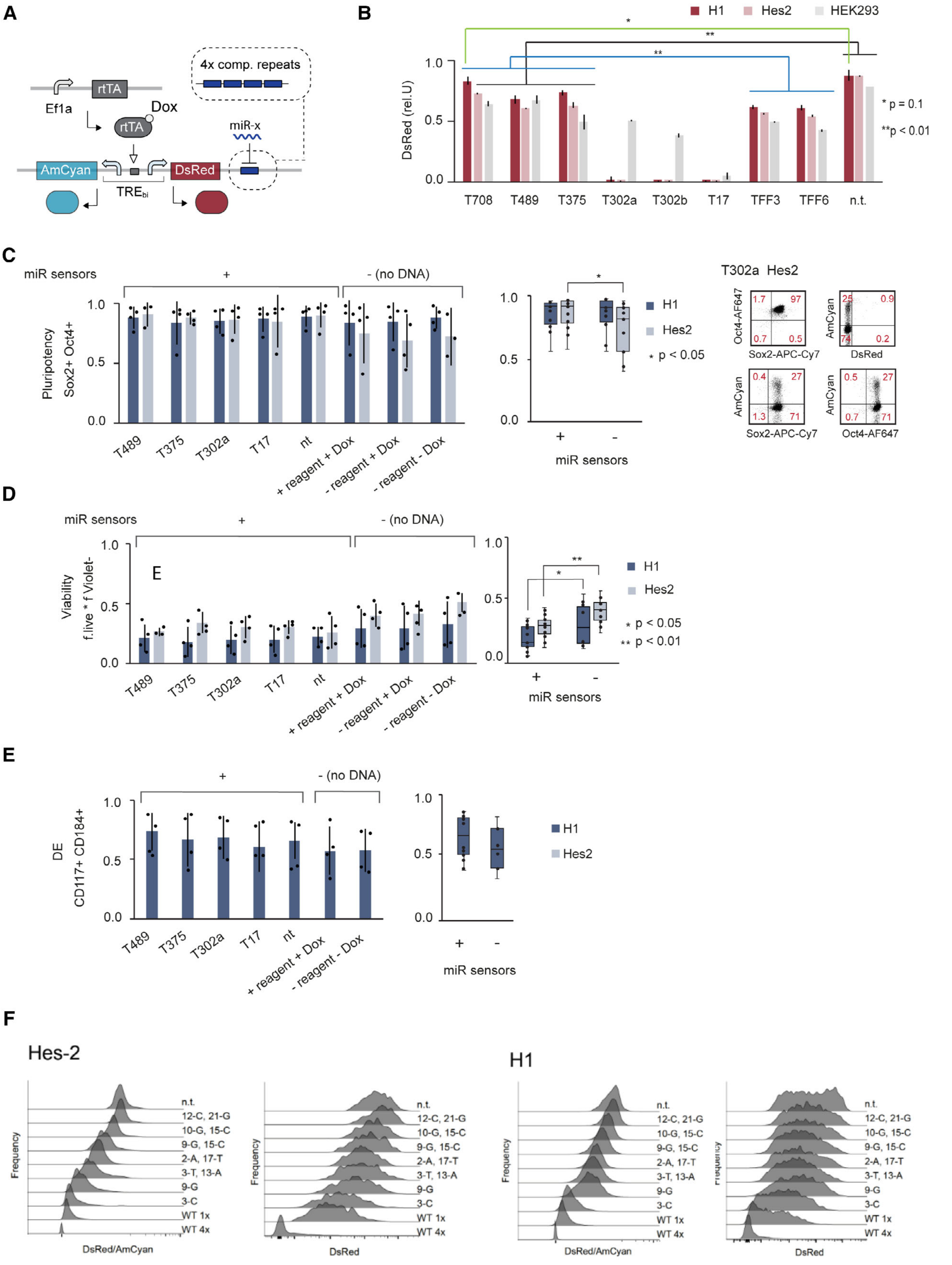

Figure EV2.

© 2022 The Authors

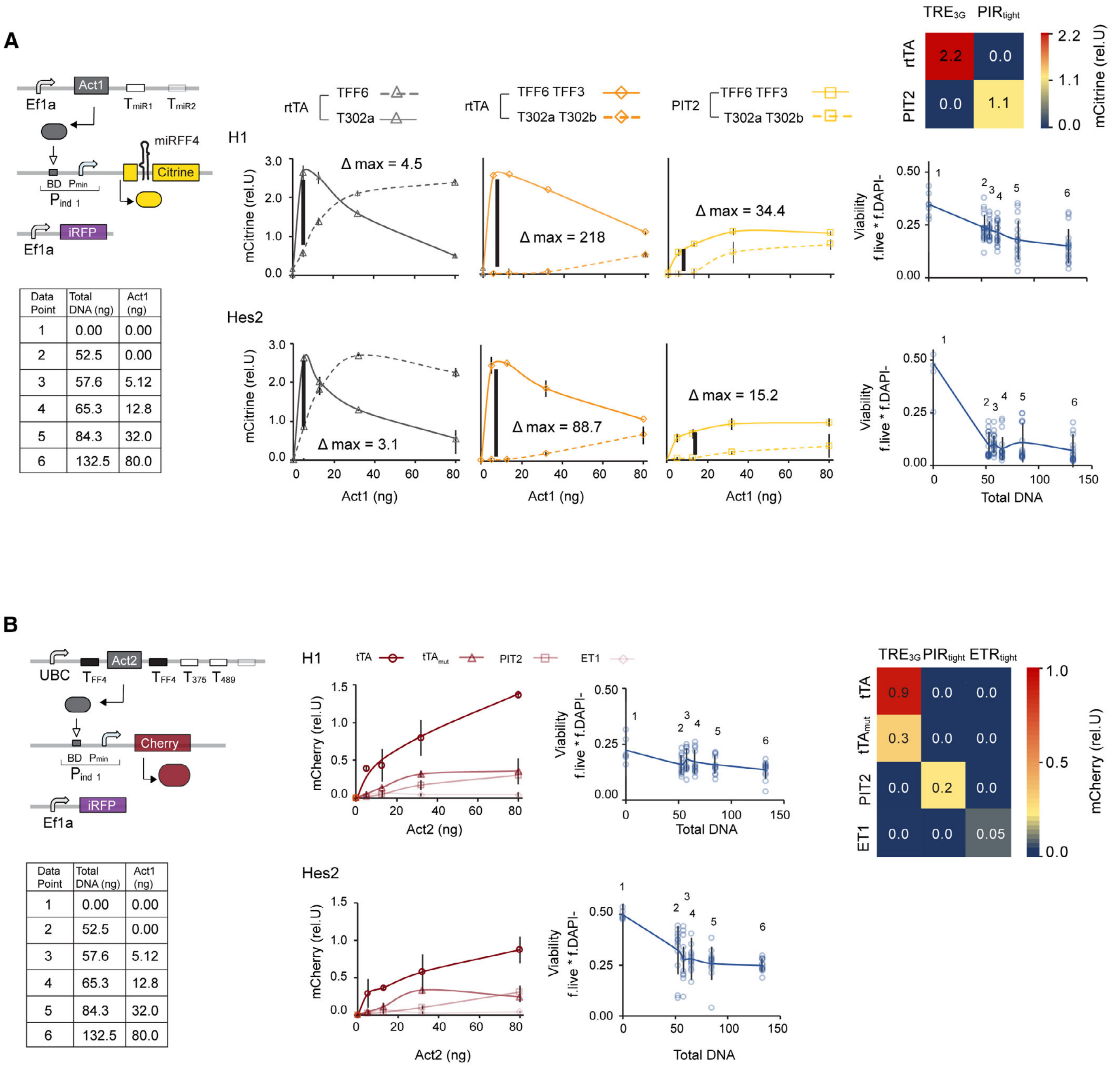

Data Point	Total DNA (ng)	Act1 (ng)
1	0.00	0.00
2	52.5	0.00
3	57.6	5.12
4	65.3	12.8
5	84.3	32.0
6	132.5	80.0

Data Point	Total DNA (ng)	Act1 (ng)
1	0.00	0.00
2	52.5	0.00
3	57.6	5.12
4	65.3	12.8
5	84.3	32.0
6	132.5	80.0

Figure EV3. Circuit module characterizations.

A, B Characterization of the FF4 inducible (A) and output inducible (B) module, respectively. Dose response function (middle) showing normalized fluorescence readouts of mCitrine and mCherry to changing Act1 and Act2 plasmid amounts, respectively. Curves serve as visual guides. Charts show the mean ± s.d. of at least three biological replicates. Viability as a function of DNA amount (left). Single data from all samples are plotted as circles. Mean values are indicated as dots. Line serves as a visual guide connecting mean values. Color plots show cross-activation of each transactivator with each promoter using 32 ng of a given transactivator.

Source data are available online for this figure.

© 2022 The Authors

Figure EV4. Extended analysis of experimental combinatorial screen.

A Experimental combinatorial screening of Act1 and Act2 in three different circuit configurations: PIT2-tTA using high levels of Act1 (left, data shown in Fig 3C), rtTA-PIT2 using high levels of Act1 (middle) and rtTA-PIT2 using low levels of Act1 (right, data shown in Fig 3C). For each circuit and each Act1 concentration, the normalized mCitrine/FF4 level (yellow) and the normalized Output levels (mCherry) (red) are shown in response to changing amount of Act2 expressing plasmids in the Off configuration (Act1 targeted by TFF3/TFF6) (solid lines) and On configuration (Act1 targeted by T302a/T302b) (dashed lines).

B Scatter plots showing normalized mCitrine expression in response to changing amounts of Act1 expressing plasmids across all Act2 levels of each circuit depicted in (A). Line connects mean values and serves as a visual guide.

C Bar chart of experimental data shown in Fig 3C. PIT2-tTA circuit using high levels of Act1 (left) and rtTA-PIT2 circuit using low levels of Act1 (right).

Data information: All charts show mean ± s.d. of at least three biological replicates. Related to Fig 3C.
Source data are available online for this figure.

 © 2022 The Authors

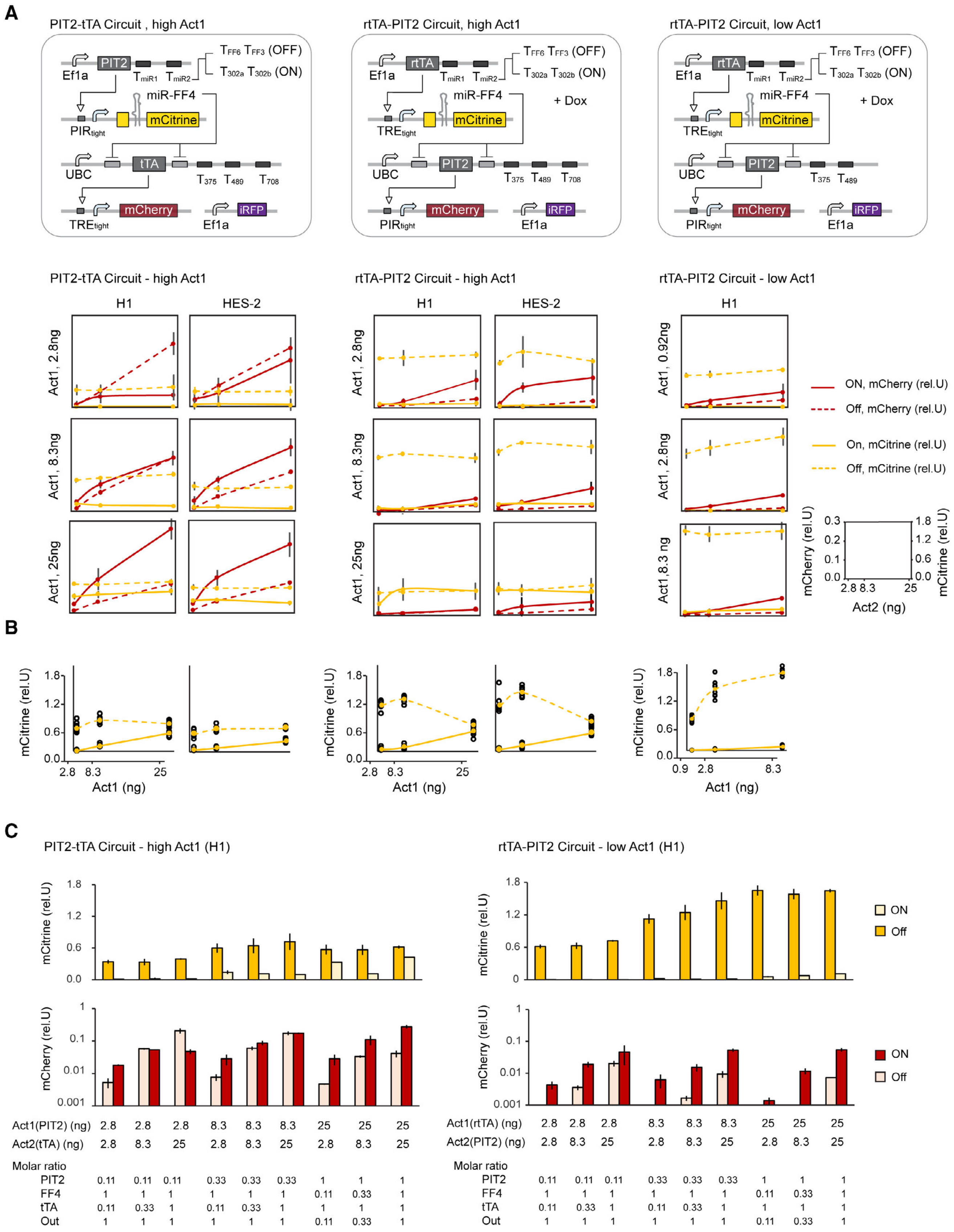
A
PIT2-tTA Circuit , high Act1
rtTA-PIT2 Circuit, high Act1
rtTA-PIT2 Circuit, low Act1
Ef1a
PIT2
rtTA
TFF6 TFF3 (OFF)
T302a T302b (ON)
miR-FF4
+ Dox
PIRtight
TREtight
mCitrine
UBC
tTA
T375 T489 T708
mCherry
iRFP
PIT2-tTA Circuit - high Act1
rtTA-PIT2 Circuit - high Act1
rtTA-PIT2 Circuit - low Act1
H1
HES-2
Act1, 2.8ng
Act1, 8.3ng
Act1, 25ng
Act1, 0.92ng
Act1,8.3 ng
ON, mCherry (rel.U)
Off, mCherry (rel.U)
On, mCitrine (rel.U)
Off, mCitrine (rel.U)
mCherry (rel.U)
mCitrine (rel.U)
Act2 (ng)
B
mCitrine (rel.U)
Act1 (ng)
C
PIT2-tTA Circuit - high Act1 (H1)
rtTA-PIT2 Circuit - low Act1 (H1)
mCitrine (rel.U)
mCherry (rel.U)
ON
Off
Act1(PIT2) (ng) 2.8 2.8 2.8 8.3 8.3 8.3 25 25 25
Act2(tTA) (ng) 2.8 8.3 25 2.8 8.3 25 2.8 8.3 25
Act1(rtTA) (ng) 2.8 2.8 2.8 8.3 8.3 8.3 25 25 25
Act2(PIT2) (ng) 2.8 8.3 25 2.8 8.3 25 2.8 8.3 25
Molar ratio
PIT2 0.11 0.11 0.11 0.33 0.33 0.33 1 1 1
FF4 1 1 1 1 1 1 0.11 0.33 1
tTA 0.11 0.33 1 0.11 0.33 1 1 1 1
Out 1 1 1 1 1 1 0.11 0.33 1

Figure EV4.

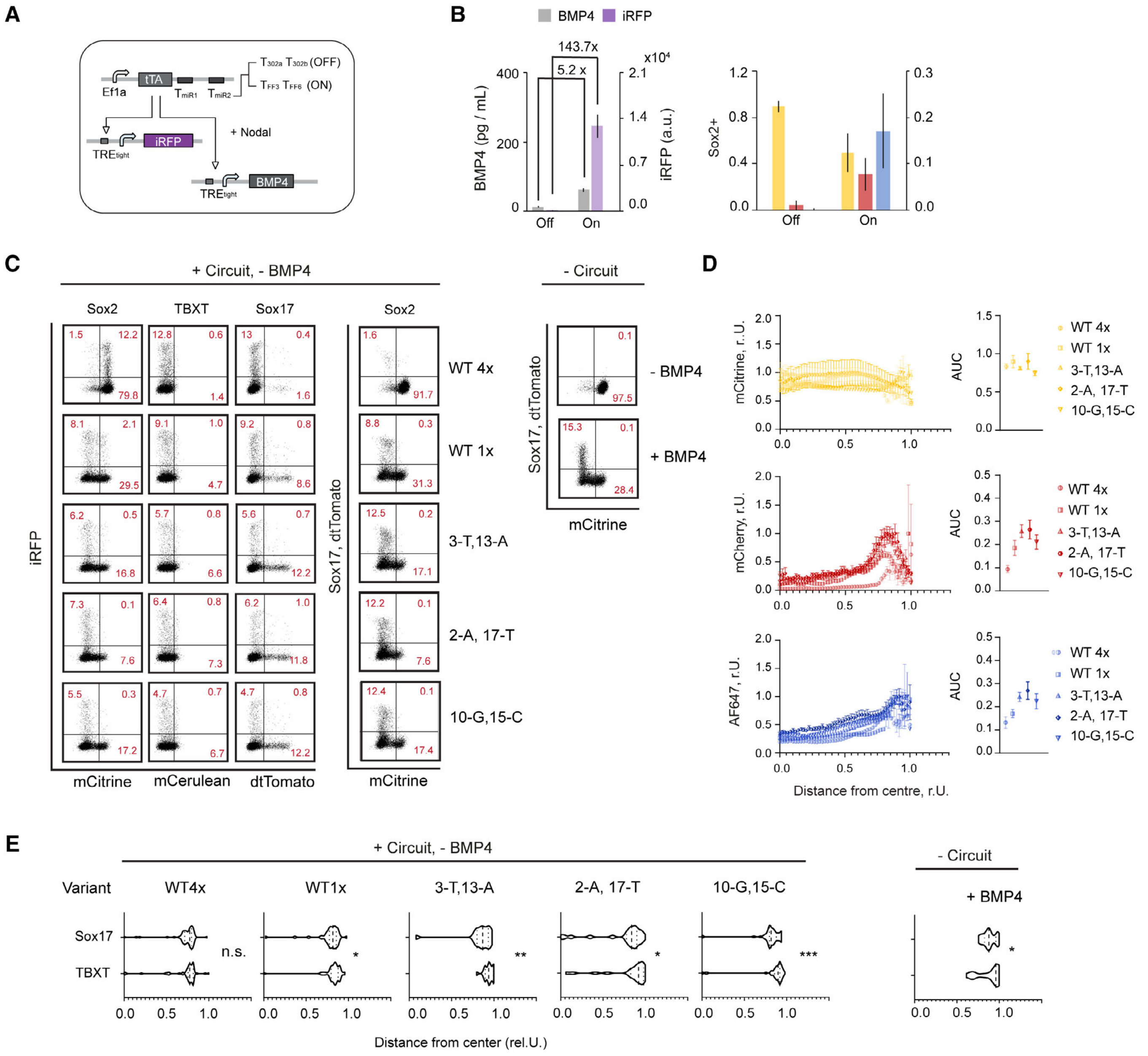

Figure EV5. Extended analysis of BMP4-mediated cell composition control.

A Circuit schematic.

B Control of BMP4 in response to endogenous miRNAs. Micropatterned RUES2 have been transfected with in (A) illustrated On and Off circuits using tTA at 2.8 ng/96 well (in contrast, data in Fig 6B used tTA construct at 25 ng/96 well). Bar chart on the left shows average BMP4 concentration released from each circuit as determined by ELISA and absolute iRFP expression as measured by Flow Cytometry. Each bar corresponds to mean ± s.d. from three biological replicates. Bar chart on the right shows the average fraction of cells expressing a given germ-layer marker analyzed from at least 20 colonies from Confocal Microscopy images. Related to Fig 6B.

C Representative Flow Cytometry scatter plots for microscopy data depicted in Fig 6D–F.

D Radial marker profiles of Sox2, TBXT and Sox17 for all miSFITs variants (left) and associated AUC values (right) of data depicted in Fig 6D–F.

E Spatial divergence of Sox17 and TBXT of data depicted in Fig 6D–F.

Data information: Charts in (C–E) show average ± standard error calculated from at least 20 colonies.

© 2022 The Authors
