# Supplementary material for: Synthetic gene circuits for cell state detection and protein tuning in human pluripotent stem cells
Source: Mol Syst Biol. 2022 Nov 11;18(11):e10886. doi: 10.15252/msb.202110886 (PMC9650275; doi:10.15252/msb.202110886)
Supplement: Supplementary file 11 [file MSB-18-e10886-s010.pdf]

Synthetic gene circuits for cell state detection and protein tuning in human pluripotent stem cells

Laura Prochazka, Yale Michaels, Charles Lau, Ross Jones, Mona Siu, Ting Yin, Diana Wu, Esther Jang, Mercedes Vázquez-Cantú, Penney Gilbert, Himanshu Kaul, Yaakov Benenson, and Peter Zandstra
DOI: 10.15252/msb.202110886

Corresponding author(s): Peter Zandstra (peter.zandstra@UBC.ca)

Review Timeline:

Submission Date:	21st Dec 21
Editorial Decision:	22nd Feb 22
Revision Received:	21st Aug 22
Editorial Decision:	14th Sep 22
Revision Received:	13th Oct 22
Accepted:	17th Oct 22

Editor: Jingyi Hou

Transaction Report:

(Note: With the exception of the correction of typographical or spelling errors that could be a source of ambiguity, letters and reports are not edited. Depending on transfer agreements, referee reports obtained elsewhere may or may not be included in this compilation. Referee reports are anonymous unless the Referee chooses to sign their reports.)

Thank you for submitting your work to Molecular Systems Biology. We have now heard back from the three reviewers who agreed to evaluate your manuscript. As you will see from the reports below, the reviewers acknowledge the interest of the study and appreciate the high quality of the presented data. They raise however a series of concerns, which we would ask you to address in a major revision.

The recommendations of the reviewers are rather clear, so there is no need to repeat the points listed below. In particular, most of Reviewer#1's concerns refer to the need to provide further clarifications and to improve the presentation of the study in order to make the data and the main conclusions easily accessible to the readers, which we would ask you to adequately address within reason. All other issues raised by the reviewers need to be satisfactorily addressed as well. As you may already know, our editorial policy allows in principle a single round of major revision, and it is therefore essential to provide responses to the reviewers' comments that are as complete as possible.

On a more editorial level, we would ask you to address the following issues:

Reviewer #1:

OVERVIEW
The manuscript by Prochazka and colleagues is a detailed exploration of potentially useful synthetic classifiers network circuits in hiPS cells for sensing of miRNA-based cell pluripotent stem cell state, and induction of user-defined graded responses of a reporter gene; moreover it contains also an application of simpler circuits for the production of functional morphogenetic proteins (BMP4), which shows phenotypic effects in a hiPS cell differentiation assay.

This area of research is extremely exciting given the impression in the field that circuits for controlling stem cell differentiation will be transformational; since these circuits are so far limited in scope and applicability, and particularly rational design of cell sensing and responses control circuits, those represent a high demand in the field. Although the paper is a thorough experimental and computational tour de force with extremely high quality data (highlights include validation of majority of experiments in 2 hiPS cell lines, quantitative comparison of model with data, thorough presentation of aggregate as well as raw metrics); in the current status of the manuscript, it presents a somewhat disjointed narrative that makes it feel rather far from being ready for publication; at this stage is even hard for me to imagine how the final product will look like. I try to explain below better what I mean by that, but for example, it almost seems that the authors have materials for 2 papers (or 1 and a half) in here, but the materials are presented in a non-well-organized manner. The major difficulty for me derived from the fact that it was very hard to identify what exactly the authors are claiming (I offer examples below). It is possible (even likely I would say) that, with a thorough rewriting of the presentation of the data, the manuscript could be of broad interest. In the current status though, I cannot provide a sound recommendation as I do not know exactly what the authors are claiming they found in their experimental exploration.

As a personal opinion, I offer that to me, these kinds of papers have the chances to be impactful in 2 ways: (1) someone else in the community will use the circuits presented here, as they are, for similar applications; (2) someone else in the community will use a pipeline similar to the one followed by the authors to develop novel circuits, for example for other state-specific circuits, improved version, etc.. In the current version impact (1) is reduced as the logic is quite hard to parse, and ultimately it is not clear what the authors would say about the diverse array of circuits that are presented in the paper, whether for example there is one specific architecture that is robust over some others that are not in the different contexts; (2) the pipeline followed by the authors is really hard to follow, as the logic progression of how the authors went about it (or rationalized their path) progresses in a expansion/restriction way, and meandering between in vitro and in silico was that seems hard to be able to follow. Given these limitations, it's hard for me to recommend this current manuscript for publication, although I repeat, the data are amazing. Another consideration that I thought about was whether the content of the paper is a good fit for the journal; my understanding is that the scope of Molecular Systems Biology in understanding the dynamics of natural living systems, and less towards creating and characterizing synthetic ones. I'll let the editor weigh into how much to take this into consideration, as is more of an editorial decision in my opinion. My technical opinion is that there is not much provided here that constitutes an advancement in understanding the dynamics of natural living systems.

SUMMARY
I provide here my understanding of the main messages of the figures of the paper, to facilitate the explanation of my difficulties with it.
Summary:
Fig. 1, abstract logic of the circuits design for complex, digital input detection and graded output responses.
Fig. 2: computational identification of components for input for discrimination of hiPS cell state vs differentiated cell.
Fig. 3B: confirmation that the sensors based on those 3 miRNA work in hiPS as expected.
Fig. 3C, validated modifiers for graded expression of output based on miRNA17 in hiPSC
[2 and 3 could go together]
Fig 4: computational exploration of parameter impact on network behaviors concerning the inputs
Fig. 5: implementation of 2 version of circuit with parameters identified computationally, which display input control; identification of a version that works better (rtTA-PIT2)
[Fig. 4 and 5 could go together]
Fig. 6, generation of a complete digital input graded output in hiPS. The best performer from Fig. 5 is chosen with the addition of T17 binding sites variants for output control; alongside 2 control circuits.
IMO 6E: is the climax of the paper which offers the example of combination of power of discrimination (input) + graded output!!
Fig. 7: implementation of one simple circuit for secretion of BMP4 based on miRNA-controlled expression, which is shown to affect differentiation.

EXAMPLES OF HARD-TO-PARSE TEXT/FIGURES PASSAGES

Introduction on miRNA-low/high (bottom of page 4): very hard to understand why one would want one or the other. And how it relates to the circuits that the authors will present during the rest of the paper: which of the circuits implemented in cells implement the miRNAlow/high logic?

Fig.1A describes a circuit that checks if cells are in hPSC state, and then produces a fine-tuned output; the text at the bottom of page 3 says "Here we describe the design- and implementation strategy of synthetic gene circuits that are capable of performing cell state specific control of desired proteins in hPSC", which is a much more general claim. What are the authors claiming their results show?

Bottom of page 3: "Mechanistically, our system integrates an input module that uses discrete logic gate computations [35] to detect a set of hPSC-specific miRNAs, with an output module that operates with miRNA silencing-mediated fine tuners (miSFITs) that allows to pre-select for desired output dose [45]." So the input is state-dependent, and the fine tuners are also state-dependent? Or user-decided? Not clear to me at this point.

Fig. 4. it is really hard for me to understand why this is important, as it seems it relies heavily on previous literature, and gives a rather hand-waving explanation here: Beginning of Page 9:"Reasons include the length of the signaling cascade, the unfavorable dynamics of certain components and substantial variation in expression strength, growth rate and transfection efficiency across different human cell types." That leaves me thinking: why do they need this? What exactly are they looking for in their computational screening? Fig. 4 is also the first time that I saw miR-FF4; which is not explained in the text how it relates to the previous circuits and why it is needed.

Last paragraph page 9 to end of Page 10; is really really hard to follow: goes from we're implementing in vitro the circuit we found computationally, to a setup of a combinatorial in vitro screen, to another computational evaluation (Fig. 5B), back to the description of the circuit for implementation (Fig. 5A).

In circuits in Fig. 5A there are a number of novel T, e.g. FF6, FF3, 302b, ... whose logic function have not been introduced or explained sufficiently in the text.

At the end of description of Fig. 5D would love to see a sentence that informs the reader about that the authors think about the 2 circuits presented in Fig.5A: do they like them? Are they good? One better than the other? For what reasons? Are they going to continue one or the other in the continuation of the paper? Why yes/why no?

And then after Fig. 5F: do the author think that miRNAlow is better than miRhigh sensors and so decide to continue with those? If the circuit functions even better with miRlow sensors only, rendering the miRhigh part obsolete, then the entire paper focus shifts towards miRNA-mediated output silencing instead of a synthetic circuit. In addition, the original classifier paper (Xie et al. 2011, Science) shows that classifier circuit performance increases with the inclusion of more miRNA markers, raising questions regarding the selectivity/specificity of this miRlow-only configuration in pluripotent vs differentiated hiPSCs. Despite this auspicious verdict of this miRlow-only configuration, Figure 6 uses a miRhigh+miRlow circuit, but Figure 7 uses a miRlow-only module, so it is unclear what the take-home message is.

Beginning of Page 13: "Because miRNA and LNA-administration into hPSC has proven difficult using our transfection protocol (Figure S6A-D), we characterized the circuit performance in response to all input miRNAs in HEK293 (Figures 6D and S6C)." But then in the figure I see a panel for H1 cells and one for HEK293; why the discrepancy? Did they do or did they not do the experiment in H1 cells?

on page 13: "we found that addition of miR-375 and miR-489 mimics can overcome the remaining leakage" What is defined as remaining leakage here? Leakage of what? I thought that the fact that mCherry goes down by adding miR375 was a feature of the system, not a bug?

Last paragraph of page 14. "To sum up, our data demonstrate that the hPSC-specific circuits produce substantially higher outputs compared to non-hPSC cells, such as HEK293, and are responsive to all four miRNA inputs." Is a key sentence, which I found really hard to parse. It is one of the key claims of the paper, and given I don't know how to parse it, it's very hard for me to develop an opinion as of whether the data support the claim or not. Examples of claims that could be clearer to wrap my head around: circuit XXX produces output specifically in hPSC cells and not HEK293 cells; circuit YYY can be responsive to 4 miRNA inputs. ...

6F: why is circuit 1 analysis in H1 cells missing? That seems it should be the climax of the paper so far. The data on circuit 3 presented for multi-output prevents the authors to use the broader claim that their circuit can do multi-input with multi-output.

Fig. 7A. Is the circuit introduced in hiPS for functional gene expression been presented/characterized before in this paper? Hard to tell. This seems a circuit that does not discriminate anything in input, it only does the output control; right? If that's the case, how does it relate to the class of circuit that the authors have been talking so far in the paper?

Fig. 7C what is in the y axis of the graph? Why is it so dramatically different between Sox2 and the other markers (4orders of magnitude)?

Fig. 7D/E What happens to the "black" regions in the circuit+, BMP4- conditions? Are there cells? Are cells alive/dead? Potentially interesting phenotype, lack of characterization makes it of questionable interest

Fig. 7E now checks a circuit with only input control (and discrete output=BMP4). Shows that the circuits with targeting vs non-targeting miRNA have the expected output and phenotypic effect regarding differentiation. These data do not show that one circuit is able to differentiate different cell states, but rather that the same cell state can be read differently by 2 circuits. To support a claim that a circuit is able to discriminate different cell states the right experimental setup seems (to me) show that the same circuit works differently when the cells are in 2 different states; e.g. if the authors differentiate those cells with the off circuit, do they start expressing BMP4? If the claim is that the functional circuits with input/output control can be implemented for affecting functional differentiation in hiPS, this needs to be shown. Or the claim can be something else.

Middle of Page 17."In summary, our data indicate that the minimal version of a discrete-to-analog circuit". What is the circuit that the authors have in mind here? 7A? or 7E? Or a combination of the 2? The conclusions here combine together results from different circuits, making it sound that the authors think that combining the different parts will yield the final combined circuit. Is that the case? If so what makes them confident that it is the case (from my position, I don't see why that would be the case giving difficulties with combining circuits parts, as argued by the authors in the introduction; but maybe some of the results presented here may support that confidence that I am missing?)

MINOR POINTS

Page 5 bottom:
"In order to restrict circuit action to discrete cell states or cell types, a set of endogenous miRNAs must be identified to allow clear discrimination of the cell-state of interest (positive samples, here hPSC) from the other cell states (negative samples, here hPSC- derived differentiated cells) within a testing space." Not clear to me from what is presented here why it "must" be identified, or what kind of endogenous miRNAs set are we looking for here (cell-type specific?); consider rewording; maybe it is "the set of endogenous miRNAs that allow clear discrimination of cell states of interest must be identified"? Hard to parse as it is.

Fig. 2
Figures 2A-B: It would be appreciated that the ordering of samples follows the intuitive course of differentiation, i.e. starting from Day0 on the left, and increasing incrementally until it reaches Day10 on the right. Abbreviations for MPC, NPC, RNCS, EB should be provided in the figure legend. The MPC, NPC, RNCS, and EB datasets, while included in the analyses, are not mentioned in the main text; what purpose do they serve regarding hiPSC-specific miRNA identification on top of Day1-10 differentiation datasets?
Figure 2B shows that miR-375 should supress the synthetic output when hiPSCs differentiate towards pancreatic cells or, at the very best, definitive endoderm. There are however no explorations or comments regarding how the output would be supressed in ectodermal and mesodermal lineages. Together with the fact that Figure 2 is purely computational without any experimental validation, these points raise questions regarding the circuit's performance.
In the graphs output (mol/cell) over Diff-hiPSC, there are always some point (cells I guess?) that give a high output for any circuit, for the non-hiPS cells; why? Is that a problem?
I am not familiar with this kind of clustering and have had hard time finding quantitative definitions as well as high-level interpretative help for the following: AUC, Truth, Expr ratio over t(log2)

Fig. 3
3B and C, would be informative I think to see histograms of DsRed alone
in figures: what does T489, T375, ... mean? Not defined in fig nor in figure legend nor in text (that I can find)
For Figure 3B and particularly 3C, was there any normalization applied to ensure that the test groups were equivalent in the stoichiometry of miRNA:construct, so that differences in the plotted results are rooted only in the variation of the miR-17 site sequence?
in figure3C: what does 3-C mean?
When testing the analog miR-17 variants in hiPSCs, the authors comment "Compared to the HES-2 line, the H1 line showed significantly higher DsRed expression for most sensors". This is interesting and deserves some elaboration, even if hypothetical: what is the reason, according to the authors, that different hiPSC lines have different miRNA activity/profiles? Would this line-to-line variation compromise the circuit's reliability in performance?

Fig. 4
"Act1 performs similarly to the model presented in Schreiber et al, achieving a high On/Off ratio whenever KD or Act1MAX is not particularly low". This seems counter-intuitive to me: wouldn't a low KD indicate high-affinity of Act1 for Pind1, therefore sensitivity of Pind1 to Act1, and a high or sudden On/Off ratio in response to increases in Act1 levels?
"Third, we chose a relatively weak promoter (UBC [52]) to express Act2 to keep Act1MAX levels low (Figures 4 and S3)" do the authors mean Act2MAX instead of Act1MAX?

Figure 4B, plot showing On:Off ratio vs MiR-FF4MAX concentration is interesting: firstly, it seems to be following Michaelis-Menten instead of Hill kinetics. Secondly, what practical information does the simulation give; e.g., what is the concentration or amount of miRNA cells should express for the circuit to function with a strong On:Off response?

Fig. 5
Figure 5A, 5E: Why are the miRlow sensors placed at the 3'UTR of Act2 and not the output?
Why is the best FF4 Off/On performance at low Act1MAX amounts (Figure 5B)? Is it the same reason as the output performing optimally with low Act2MAX (so that they are completely covered by the miRNAs)?
"We conclude that FF4 production is not high enough in most of the combinations to sufficiently repress Act2 in the PIT2-tTA circuit leading to unexpected circuit behaviour for this configuration (Figures 5C and S5A)" how are FF4 and Act2 quantified and compared stoichiometrically to reach this strong conclusion?
The trade-off between maximum expression levels vs maximum inducibility of the miRNA circuits reflects that of mammalian synthetic promoters (Ede et al 2016, ACS Synth Biol.). Seems could be great to hear the authors elaborate on this.

Fig. 6
Figure 6D: Addition of scrambled miR-Ctrl in "HEK-293 Ciruit 1" seems to slightly increase mCherry output. Also, in "H1 Circuit 1", addition of miR375 or miR489 seem to reduce mCitrine levels, which is unexpected given these miRNAs regulate UBC::PIT2 which lies downstream of the TRE::miRFF4-mCitrine module?
Bottom of page 12: typo (I think): (Figure 6B should read Figure 6C);
fig. 6C, what is on X axis of mCitrine graphs? If it is circuits, where is circuit 3? Zero/invisible?
"Because the highest On state is already weak, by further tuning the levels down, we observed not only a shift in the mean value of the expression intensity but also in the number of cells that scored as mCherry positive". This would seem to suggest a problem in using the analog miR-17 tuner in hiPSCs: fine-tuning makes sense when we have a substantial amount of gain already, and we'd like to perform some fine adjustments; when the output is overall low, the primary aim is to add gain, then do the fine-tuning. Having said that, normalized data in Fig6E look great.

Fig. 7
Curiosity: S7B shows what happens to the cells producing BMP4: any reasons why that may be the case? Where are these cells on the discs? Where is mCitrine in the circuit? (For interpretation of Fig. S7)

Reviewer #2:

The authors engineer genetic circuits with sense-and-respond capabilities to activate expression of a desired protein in a particular cell state. Here, they utilize the cell state-specific expression of microRNAs (miRNAs) to distinguish undifferentiated human pluripotent stem cells (hPSCs) from other, differentiated cell types. They develop an automated way of analyzing omics datasets to identify miRNAs that are specifically over-expressed or depleted in cell states, followed by utilizing those miRNAs to either repress or de-repress transcription factor expression (here, Act1 or Act2). The miRNA-dependent expression of the TFs is used to classify whether the cell is in the hPSC state. The authors then utilize the Act1 and Act2 transcription factors (activators) to regulate the expression of either a fluorescent protein reporter (an observable proxy) or the BMP4 morphogen. Their best genetic circuits are able to activate output protein expression by up to 7.7-fold for a relevant effector protein (BMP4).

The authors expand on an existing model of miR-dependent and TF-dependent transcriptional regulation to guide the design of their genetic circuit. With the model, they identified the key parameters that affect circuit function. They also leveraged the model to explain why the dynamic range of the circuit is lower than desired when using a miR with a high expression level in the hPSC cell state (miR high sensor).

Overall, the manuscript is clearly written, though quite lengthy. It was particularly important that each section ends with a brief summary of the results. The following comments will improve the readability of the manuscript.

Specific Comments

Several changes to the figures will improve clarity:

1. In Figure 1, the blue box in the circuit schematic is unlabeled. Presumably, it is the Act1 or Act2 transcription factor, but it needs to be labeled. It's also important to explicitly label the miR binding sites and the Act binding sites. The red box in the genetic circuit diagram is also unlabeled. It is not clear what the red box indicates; is it a reporter protein, the output protein (e.g. BMP4), or the "miSFIT" variants? The mechanism of the miSFIT variants are not well-described. Even though their development is from a prior paper, Figure 1 needs to clearly show that other constitutively expressed miRs are being used to tune the expression of the output through design of miR binding sites in the 3' UTR.

2. In all figures, the word "predicted" or "calculated" needs to be used to denote modeling results. For example, in Figure 2, it is

not readily apparent that the middle panels are modeling calculations. Same for Figure 4B and Figure 5B.

3. In Figure 2B, it is not clear why the miR-375 expression ratios are sorted (descending). They should be sorted by progression over Days. Otherwise, it is confusing and potentially misleading.

4. The measurement scales in Figure 5C and 5D vary greatly across the experiments, leading to potentially misleading color schemes. For example, for the PIT2-rTA circuit, the "Off" state varies from 0.3 to 0.7 for the mCitrine measurement and from 0 to 0.2 for the mCherry measurement. Even though mCitrine and mCherry measurements exist on different scales, the lower limit should always be zero. Further, it's not clear at all if the difference between 0 and 0.2 is statistically significant or whether it's all "zero" within noise. Unlike the authors use of bar plots in other panels, there are no error bars in Figure 5CD, which makes it impossible to determine significance.

Typos:

"The here reported designs, plasmid libraries and optimization approach provides" should be "Here, the reported designs, plasmid libraries and optimization approach provide"
Figure 5F. "senor" should be "sensor"

Reviewer #3:

This manuscript aims to develop gene circuits that sense and distinguish the stem cell state from the differentiated cell state and mount appropriate responses. Two genetic modules are designed to achieve this aim. The input module consists of sensors of high and low microRNA (miR) levels, which converge onto an AND gate to define cell state based on the presence and absence of various miRs. Here, computational algorithms identify the absence of miR-489 and miR-375, combined with the presence of miR-302 as a signature of human pluripotent stem cells (hPSCs). The output module can be off or on, according to the input module's decision. The output level is adjustable by using various numbers and sequence variants of the native miR-17 target site. Various circuit configurations are considered and optimized using a computational model introduced earlier (Ref. 46). Following the optimal design, circuit function is demonstrated in hPSC versus other cell lines, and applied to control endogenous BMP4 secretion in culture, which can affect cell composition and morphogenesis.

This is an interesting, application-oriented interdisciplinary study that is carefully executed and presented. Considering the potential for future applications, the manuscript is relevant to a diverse audience. However, certain parts and terminology are unclear and there are some concerns about the experimental and computational methodology and their interpretation. Therefore, the following comments should be addressed before the manuscript is suitable for publication in Molecular Systems Biology.

(1) The system is designed and tested using transient transfection with plasmids. This does not become clear until later in the manuscript, so it should be specified early in the Results. While it facilitates experiments, this methodology has multiple shortcomings: plasmids are taken up into cells and then lost, leading to temporal up- and downswings of plasmid copy numbers that need to be studied before precise implementation; various copies or no plasmids are taken up by different cells, causing drastic cell-cell variability that severely compromises the cell-to-cell reproducibility of circuit responses even after gating out cells without plasmids. All these effects and their effects on circuit function should be acknowledged and either studied at the single cell level to properly define the capabilities of the system, or their omission should be carefully justified and discussed. The possibility of integration, and its pluses and minuses should be discussed.
(2) According to the previous point, appropriate mathematical models are needed. To capture temporal changes in plasmid copy numbers and RNA/protein levels, time-dependent ordinary differential equations (ODEs) are needed. To capture cell-cell differences in plasmid copy numbers and RNA/protein levels, stochastic models are needed. As opposed to this, modeling in this manuscript is based on Schreiber et al. (46), which consists of a simplistic set of non-cooperative Hill function dependencies describing all interactions (transcription factor and miR effects), incapable of capturing either temporal changes or cell-cell differences. The description of the model in the Results should be clearer. Alternative modeling approaches should be considered, or their omission should be carefully justified and discussed.
(3) Using the term "digital-to-analog" in the title and manuscript is unjustified and misleading. Digital-to-analog conversion in electronics refers to a single device (such as a single modem or CD player) producing an analog signal from a digital one. In contrast, here the digital signal of the input module remains fully digital at the output for each individual cell line. An individual miR-17 site variant in the output module sets the mean level of the ON state, but the output remains completely digital: the output simply reproduces the AND gate's digital outcome at some preset amplitude, without any analog conversion. For truly analog conversion, time-dependent continuous signals such as sine waves, sinc or Gaussian pulses would be needed at the output, which does not seem to be the case. The terminology should be revised, and "digital-to-analog" claims should be removed from the title and throughout the manuscript.
(4) The claim that miR-17 target site-based miSFITs "tune expression at very fine levels" is not justified. Although it may be true that miSFITs tune the *mean* expression level, but expression in any clonal cell population is much more than its mean: it is a distribution, which has a standard deviation, a skewness, a kurtosis and so on. To claim "very fine-level tuning", histograms

should be plotted in every figure with scatter plots, and at least the standard deviation, or the coefficient of variation (CV) should be studied. Scatter plots as in Figure 3B,C, or Figure 6C,E are insufficient: These scatter plots should be converted into histograms of a single fluorescent channel, and the shape and CV of such histograms should be studied. In fact, some histograms in Figure 3C (3-C, 9-G) indicate bimodality, which not only directly contradicts expression tuning, but also obviates even the possibility of analog signals at the single cell level. For fine-level expression tuning in mammalian cells, a relevant reference that would be worth citing is PMID:32167761. This system may be noisy due to ultrasensitivity of responses and sequential cascades, see PMID:12149512 and PMID:15738412.
(5) What sort of mean was used to create bar graphs from fluorescence data? The arithmetic mean of log-normally distributed or highly skewed data will be biased towards higher values. Either the geometric mean of untransformed data or the arithmetic means of log-transformed data would be a proper representation.
(6) Multiplication by cell frequency is used to balance the cell count in each channel, but the final metric will be sensitive to variations in plasmid copy numbers if parts of the circuit are distributed to different co-transfected plasmids. The individual plasmid maps with circuit components they contain (and sequences as Supporting Data) would be highly helpful and should be provided. A possible issue is the lack of technical control where the cascaded elements of the circuit are decoupled by individual plasmid transfection (to get separate baseline intensities for each cascade level in the final circuit). Taking Figure 6, the relation between iRFP and mCitrine is highly nonlinear. If multiple plasmids were used, the analysis might be dominated by cell-to-cell variability in transfection and copy numbers of individual plasmid uptake, making the results hard to interpret, especially without controls.
(7) Some trends in the figures (such as the downward trend of On levels in Figure S4) are difficult to understand and require better explanation.

Reviewer #1:

OVERVIEW

The manuscript by Prochazka and colleagues is a detailed exploration of potentially useful synthetic classifiers network circuits in hiPS cells for sensing of miRNA-based cell pluripotent stem cell state, and induction of user-defined graded responses of a reporter gene; moreover it contains also an application of simpler circuits for the production of functional morphogenetic proteins (BMP4), which shows phenotypic effects in a hiPS cell differentiation assay.

This area of research is extremely exciting given the impression in the field that circuits for controlling stem cell differentiation will be transformational; since these circuits are so far limited in scope and applicability, and particularly rational design of cell sensing and responses control circuits, those represent a high demand in the field. Although the paper is a thorough experimental and computational tour de force with extremely high quality data (highlights include validation of majority of experiments in 2 hiPS cell lines, quantitative comparison of model with data, thorough presentation of aggregate as well as raw metrics); in the current status of the manuscript, it presents a somewhat disjointed narrative that makes it feel rather far from being ready for publication; at this stage is even hard for me to imagine how the final product will look like. I try to explain below better what I mean by that, but for example, it almost seems that the authors have materials for 2 papers (or 1 and a half) in here, but the materials are presented in a non-well-organized manner. The major difficulty for me derived from the fact that it was very hard to identify what exactly the authors are claiming (I offer examples below). It is possible (even likely I would say) that, with a thorough rewriting of the presentation of the data, the manuscript could be of broad interest. In the current status though, I cannot provide a sound recommendation as I do not know exactly what the authors are claiming they found in their experimental exploration.

Thank you for your kind words “the paper is a thorough experimental and computational tour de force with extremely high-quality data” and thoughtful recommendations and commentary. We have taken these to heart and reorganized the manuscript, streamlined the data presentation and highlighted our major contributions. We hope the manuscript now better fulfills the potential you have identified.

As a personal opinion, I offer that to me, these kinds of papers have the chances to be impactful in 2 ways: (1) someone else in the community will use the circuits presented here, as they are, for similar applications; (2) someone else in the community will use a pipeline similar to the one followed by the authors to develop novel circuits, for example for other state-specific circuits, improved version, etc.. In the current version impact (1) is reduced as the logic is quite hard to parse, and ultimately it is not clear what the authors would say about the diverse array of circuits that are presented in the paper, whether for example there is one specific architecture that is robust over some others that are not in the different contexts; (2) the pipeline followed by the authors is really hard to follow, as the logic progression of how the authors went about it (or rationalized their path) progresses in a expansion/restriction way, and meandering between in vitro and in silico was that seems hard to be able to follow. Given these limitations, it's hard for me to recommend this current manuscript for publication, although I repeat, the data are amazing.

Thank you for these comments. We have worked to present the paper as a pipeline approach, outlining the design principles. Specifically, we have streamlined the presentation of the circuits and clearly divided generic circuits (that include miRhigh sensor) and minimal circuits (that operate only on miRlow sensors). Figure 1 now captures both these circuits and also includes a flowchart on the content of the paper to better understand which circuit is used throughout the paper. Please see Summary of the new Figure below. We hope that this focused presentation improves the presentation of the work.

Another consideration that I thought about was whether the content of the paper is a good fit for the journal; my understanding is that the scope of Molecular Systems Biology in understanding the dynamics of natural living systems, and less towards creating and characterizing synthetic ones. I'll let the editor weigh into how much to take this into consideration, as is more of an editorial decision in my opinion. My technical opinion is that there is not much provided here that constitutes an advancement in understanding the dynamics of natural living systems.

We feel the paper is a strong candidate for MSB and that its combined computational and experimental approach, its biological rigor, and the use of synthetic biology approaches do both advance our understanding of different modes of signaling during stem cell differentiation (i.e. the role of BMP in tissue patterning) and provide a foundational pipeline for state control during stem cell differentiation, are very relevant to this journal. To support this perspective, we note that that MSB has published a number of impressive studies deeply rooted in synthetic biology (i.e., Gorochowski, 2017, Huang et al, 2016, Dunlop et al 2011, Schreiber et al 2016, Bacchus et al, 2013, Jewett et al 2008, Church 2005, Lou et al 2010 to mention a few). We believe MSB is a good place for this paper and we are excited to add this work to our long-standing contributions to this journal.

SUMMARY
I provide here my understanding of the main messages of the figures of the paper, to facilitate the explanation of my difficulties with it.
Summary:
Fig. 1, abstract logic of the circuits design for complex, digital input detection and graded output responses.
Fig. 2: computational identification of components for input for discrimination of hiPS cell state vs differentiated cell.
Fig. 3B: confirmation that the sensors based on those 3 miRNA work in hiPS as expected.
Fig. 3C, validated modifiers for graded expression of output based on miRNA17 in hiPSC
[2 and 3 could go together]
Fig 4: computational exploration of parameter impact on network behaviors concerning the inputs
Fig. 5: implementation of 2 version of circuit with parameters identified computationally, which display input control; identification of a version that works better (rtTA-PIT2)
[Fig. 4 and 5 could go together]
Fig. 6, generation of a complete digital input ◊ graded output in hiPS. The best performer from Fig. 5 is chosen with the addition of T17 binding sites variants for output control; alongside 2 control circuits.
IMO 6E: is the climax of the paper which offers the example of combination of power of discrimination (input) + graded output!!
Fig. 7: implementation of one simple circuit for secretion of BMP4 based on miRNA-controlled expression, which is shown to affect differentiation.

We have restructured the paper based on your suggestions (see below). We believe the restructuring improved clarity and readability of the manuscript immensely and would like to thank the Reviewer for the thorough feedback and suggestions that enabled it. We also want to highlight that, due to the positive feedback from Reviewers #2 and #3, main messages and conclusions were maintained during this restructuring process.

NEW Fig.1 Circuit design. We have updated previous Figure1 and now illustrate the generic and minimal circuit. We also added a flowchart that outlines an overview of the paper, highlighting when the generic and minimal circuits are being used (Figure 1D).

NEW Fig2: Automated circuit design and miRNA validation. (Previous Fig.2 and Fig.3 merged, excluding previous Fig.2B as we feel it distracted from the main message of the paper.)

NEW Fig.3. Model-guided combinatorial screening optimizes generic circuit performance in hPSC (Previous Fig.4 and Fig.5 merged). We have streamlined that part heavily in the text as well.

NEW Fig.4 Optimized generic circuits allow cell state specific output tuning in hPSC. (Previous Fig. 6 excluding the minimal circuit characterization part that has been moved to NEW Fig.5.) This structure allows clear separation of the two different circuits (generic and minimal) and the different approaches taken to build them.)

NEW Fig.5. Minimal circuit optimization (Previous Fig.6 E and 6F)

NEW Fig.6: Circuit-mediated BMP4 secretion enables control over cell composition and pattern formation in micropatterned hPSC colonies (Previous Fig.7)

EXAMPLES OF HARD-TO-PARSE TEXT/FIGURES PASSAGES

Introduction on miRNA-low/high (bottom of page 4): very hard to understand why one would want one or the other. And how it relates to the circuits that the authors will present during the rest of the paper: which of the circuits implemented in cells implement the miRNAlow/high logic?

Thank you for the comment. We have added additional clarification on selection of miRNAlow/high sensors (Page 4&5, see below) and modified Figure 1 that now illustrates the two circuits used in this paper (Figure 1A and 1B) and their use throughout the paper (Figure 1C).

Page 4: "To establish our platform, we designed I) a generic circuit that first detects a pluripotent state, restricting circuit actuation to undifferentiated hPSCs (hPSC-On circuit) (Figure 1A), and II) a minimal circuit that represses output production in hPSC (hPSC-Off circuit) (Figure 1B). "

Page 5: "The generic circuit contains an input module composed of two kinds of miRNA sensors, here named miR_{high} - and miR_{low} sensors (Figure 1A)."

Page 6 "The generic circuit has been rationally designed where the number and combination of miR_{high} and miR_{low} sensors has been computationally predicted from miRNA expression data of hPSC and hPSC-derived cell states (Figure 1E, left). The design of the minimal circuit, in contrast, was empirical based on miRNAs and learnings from the generic circuit implementation (Figure 1E, right). "

Fig.1A describes a circuit that checks if cells are in hPSC state, and then produces a fine-tuned output; the text at the bottom of page 3 says "Here we describe the design- and implementation strategy of synthetic gene circuits that are capable of performing cell state specific control of desired proteins in hPSC", which is a much more general claim. What are the authors claiming their results show? Bottom of page 3: "Mechanistically, our system integrates an input module that uses discrete logic gate computations [35] to detect a set of hPSC-specific miRNAs, with an output module that operates with miRNA silencing-mediated fine tuners (miSFITs) that allows to pre-select for desired output dose [45]." So the input is state-dependent, and the fine tuners are also state-dependent? Or user-decided? Not clear to me at this point.

We apologize if the summary was unclear. It is correct that the input is cell-state specific, and the output expression level is specified by the user. We have modified the sentence to more clearly state what we have implemented.

Page 4: *"The platform combines miRNA-based logic gate computations [35] for cell state detection with miRNA silencing-mediated fine tuners (miSFITs) [45] to enable precise tuning of the output dose by pre-selecting desired miSFITs targeted output constru**cts."***

Fig. 4. it is really hard for me to understand why this is important, as it seems it relies heavily on previous literature, and gives a rather hand-waving explanation here: Beginning of Page 9:"Reasons include the length of the signaling cascade, the unfavorable dynamics of certain components and substantial variation in expression strength, growth rate and transfection efficiency across different human cell types." That leaves me thinking: why do they need this? What exactly are they looking for in their computational screening? Fig. 4 is also the first time that I saw miR-FF4; which is not explained in the text how it relates to the previous circuits and why it is needed.

The specific goal in this section is to increase the dynamic range (on / off ratio) of the miR$_{high}$ sensor. We have described this on page 9

Page 9: *"Thus, to implement the generic circuit version that uses computationally predicted miRNA inputs and requires a miR$_{high}$ sensor, we undertook a model-guided circuit optimization approach with the goal of increasing the On / Off ratio (or dynamic range) of the miR$_{high}$ sensor within an hPSC embedded bow-tie circuit"*.

This is important because, when individual sensors within the circuit suffer from low dynamic range, logic integration and with that accurate discrimination (or classification) of cell states becomes difficult. A low dynamic range is mostly a problem for miR$_{high}$ sensors and can appear when genetic elements, cell types or miRNA inputs are changed (as described in the introduction on page 9) and thus require optimization. By describing this optimization in the paper, we hope that others interested in implementing these circuits can use our work as guidance for implementing their own cell state specific circuit.

Fig. 4 is also the first time that I saw miR-FF4; which is not explained in the text how it relates to the previous circuits and why it is needed.

Thank you for pointing out the lack of description on miR-FF4, we have now introduced miR-FF4 in circuit design part.

Page 5: "miR$_{high}$ sensors are double inversion modules, where the endogenous miRNA represses an Act1-inducible repressor. The repressor is synthetic miRNA (miR-FF4) that does not exist in human cells [35, 46] and is designed to repress Act2. Thus, Act2 expression is induced only if a given endogenous miRNA input is highly expressed."

Last paragraph page 9 to end of Page 10; is really really hard to follow: goes from we're implementing in vitro the circuit we found computationally, to a setup of a combinatorial in vitro screen, to another computational evaluation (Fig. 5B), back to the description of the circuit for implementation (Fig. 5A).

We agree that this section was difficult to follow. We have now restructured the section and added additional explanations to motivate the transitions between computational and experimental optimization. We would like to refer to Page 9-12 and NEW Figure 3 for the new section on circuit optimization.

In circuits in Fig. 5A there are a number of novel T, e.g. FF6, FF3, 302b, ... whose logic function have not been introduced or explained sufficiently in the text.

We thank the reviewer for highlighting the lack of description on these elements. In the revised version, the target sites have been described in the following sentences

Page 7: "....) but were significantly higher than constructs that contain mock miRNA target sites FF3 or FF6, target sites that will not be bound by any endogenous human miRNA*"*

Page 10 "*.... we designed a new with a construct that detects both miR-302a and miR-302b where miR-302b is, like miR-302a, highly expressed in hPSC. This was done by placing four fully complementary target sites of both miRNAs (T302a,T302b) in the 3'UTR of Act1, thereby forming an OR-gate*"

At the end of description of Fig. 5D would love to see a sentence that informs the reader about that the authors think about the 2 circuits presented in Fig.5A: do they like them? Are they good? One better than the other? For what reasons? Are they going to continue one or the other in the continuation of the paper? Why yes/why no?

We agree that such a summary is useful and added the following description at the end of the paragraph

Page 12: "*Overall, the rTA-PIT2 circuit is the preferred circuit configuration because it shows computationally predictable signaling performance. We selected the optimal molar ratio of components rtTA:FF4:PIT2:Cherry to be 0.11:1:0.33:1, at which we achieved the 2nd highest On/Off ratio and increased On state level compared to configuration with the highest On/ Off levels (Appendix Figure S5D)..*"

And then after Fig. 5F: do the author think that miRNAlow is better than miRhigh sensors and so decide to continue with those? If the circuit functions even better with miRlow sensors only, rendering the miRhigh part obsolete, then the entire paper focus shifts towards miRNA-mediated output silencing instead of a synthetic circuit. In addition, the original classifier paper (Xie et al. 2011, Science) shows that classifier circuit performance increases with the inclusion of more miRNA markers, raising questions regarding the selectivity/specificity of this miRlow-only configuration in pluripotent vs differentiated hiPSCs. Despite this auspicious verdict of this miRlow-only configuration, Figure 6 uses a

miRhigh+miRlow circuit, but Figure 7 uses a miRlow-only module, so it is unclear what the take-home message is.

Yes, technically, the circuit operating with only miR_{low} sensors (named minimal circuit in the revised version), as shown in NEW Figure 5, functions better (higher dynamic range and higher On state signal) than the circuit that includes a miR_{high} sensor (named generic circuit in the revised version). However, from an application perspective, this is not necessarily relevant because the number of miR_{high} and miR_{low} sensors that are required to discriminate a desired cell state (here PSC) from the rest of the samples (negative samples, here hPSC-derived differentiated cells) depends entirely on the application and with that on the number and kind of negative samples and their underlying miRNA expression profile. As outlined in NEW Figure 2A, in some cases, many miRNA inputs and combinations of miR_{high} and miR_{low} are required to properly classify. This is mostly the case if your cell state of interest needs to be discriminated from a high number of negative samples and / or from closely related cell states. In our paper, we exemplify this scenario with the generic circuit (that is composed of miR_{high} and miR_{low} sensors). Yet, in other cases, it might be sufficient to discriminate with only one, or multiple $miRNA_{low}$ sensors. This is often the case if your cell state needs to be discriminated from only a few and / or very distantly related cell states. In our paper, we exemplify this scenario with the minimal circuit that operates with only two miR_{low} sensors. In both cases, consistent with the classifier paper (Xie et al, 2011), the higher the number of miRNA inputs, the higher the specificity (see also Supplementary Information Figure S1).

Although, here we have selected the miRNAs for the minimal circuit empirically, we suggest, if miRNA expression data are available, to use the automated circuit design tool for miRNA selection as described for the generation of the generic circuit. As a guidance, we recommend to select first the simplest circuit version proposed by the algorithm (pruned version). If no classification can be achieved experimentally, more complex circuits can be tested with preference in adding additional miR_{low} sensors first and implementing miR_{high} sensors only if necessary.

In our revised version, we have implemented statements to clarify the use of the two different circuits.

Page 16 (New Figure 5): "*To sum up, minimal circuits can be rapidly created and optimized with a single titration experiment. These circuits are not just easier to create but also largely exceed the On state expression and dynamic range compared to generic circuit and are therefore recommended to use if the cell state can be effectively discriminated without miR_{high} miRNAs.*"

Page 20 (Discussion): "*Therefore, we recommend an implementation scheme, where the simplest circuit version proposed by the algorithm is being selected, with preference for circuit input combinations that do not require miR_{high} sensors.*"

Figure 6 (NEW Figure 4) only uses a miR_{low} circuit as a control circuit to measure maximal output production.

Figure 7(NEW Figure 6) uses only a miR_{low} module because the generic circuit (with miR_{high} sensor) leads to heavily reduced output (here BMP4 production) which would not allow us to test the effect on cell composition control using our transient transfection setup. Therefore, Figure 7 (NEW Figure 6) only showcases how the two functions, output tuning and cell state identification, can be applied to control physiological effector molecules for cell compositions as hPSC differentiate. Implementing a real-world

application with the entire circuit (i.e., one that can do both cell state identification and produce effector molecules at desired dose would be a natural next step in the development of our pipeline.

Beginning of Page 13: "Because miRNA and LNA-administration into hPSC has proven difficult using our transfection protocol (Figure S6A-D), we characterized the circuit performance in response to all input miRNAs in HEK293 (Figures 6D and S6C)." But then in the figure I see a panel for H1 cells and one for HEK293; why the discrepancy? Did they do or did they not do the experiment in H1 cells?

We apologize for this unclear description. We performed the experiment in H1, but found that miRNA mimics can repress circuit constructs only at very high (typically unphysiological) doses (NEW Figure 4D, Appendix Figure S2). LNAs, in contrast, did not show any de-repression at all (Appendix Figure S2D). Thus, we were not able to verify if our circuit, and in particular the miR_{high} sensor, was indeed functional and responsive to its input miRNAs. Thus, we tested the circuit responses to changes in miRNAs in HEK293. To make the description clearer, we have modified the sentence to the following:

Page 13: *"We found that the addition of miR-375 and miR-489 mimics each fully repress output production in hPSC (Figure 4D). However, we note that administration of miRNA mimics and siRNAs has proven difficult (Figue S6A-C) and LNA-administration completely ineffective (Figure S6D) using our transient transfection setup in hPSC (Appendix Figure S2A-C). Therefore, we have characterized the circuit performance in response to all input miRNAs in HEK293 (Figures 4D and S2C)."*

on page 13: "we found that addition of miR-375 and miR-489 mimics can overcome the remaining leakage" What is defined as remaining leakage here? Leakage of what? I thought that the fact that mCherry goes down by adding miR375 was a feature of the system, not a bug?

Thank you for this helpful comment. Yes, it is correct that the observed reduction of mCherry by adding miR-375 is a feature of the system. However, here we optimized the circuit to operate as predicted in hPSC and now validate the performance in response to miRNA inputs. In an ideal world, the miR_{high} sensor should function without producing an Off state. However, as described in previous reports, the leaky output expression in the Off state can arise from unfavorable expression dynamics of individual plasmids in a transient transfection setup (Lapique et al, Nature Chemical Biology, 2014) This means that technically, in our validation setup, miR375 reduces this technical leakage. Practically, if the circuit were to be applied for classification, addition of miR375 improves the cell-state specificity.

To avoid confusion, we have modified the sentence to the following:

Page 13: *"We found that the addition of miR-375 and miR-489 mimics each fully repress output production in hPSC (Figure 4D)."*

Page 13: *"Finally, consistent with observations in hPSC, miR-375 and miR-489 mimics can further reduce output production in HEK293."*

Last paragraph of page 14. "To sum up, our data demonstrate that the hPSC-specific circuits produce substantially higher outputs compared to non-hPSC cells, such as HEK293, and are responsive to all four miRNA inputs." Is a key sentence, which I found really hard to parse. It is one of the key claims of the paper, and given I don't know how to parse it, it's very hard for me to develop an opinion as of whether the data support the claim or not. Examples of claims that could be clearer to wrap my head around: circuit XXX produces output specifically in hPSC cells and not HEK293 cells; circuit YYY can be responsive

to 4 miRNA inputs. ...

We have modified this sentence based on your suggestion.

Page 14: *"To sum up, our data demonstrate that the generic circuit produces output specifically in hPSC but not in HEK293 and is responsive to all four miRNA inputs."*

6F: why is circuit 1 analysis in H1 cells missing? That seems it should be the climax of the paper so far. The data on circuit 3 presented for multi-output prevents the authors to use the broader claim that their circuit can do multi-input with multi-output.

We did, in fact, perform this experiment in H1 cells; this experiment required co-transfection of a large number of plasmids. Given the poor transfection efficiency in H1 relative to HEK293T at baseline, only a small number of H1 cells received the full complement of constructs. We now discuss this limitation in the text and note that realizing the full potential of these multi-component circuits will require, either improving transient delivery, or stable integration of all circuit components.

See limitation section on page 22.

Page 22: First, the current system works robustly with a high dynamic range only when miR$_{low}$ sensors are used (minimal circuits), but produces very low yield when the input module comprises a miR$_{high}$ sensor (generic circuits). Thus, the On state and dynamic range of the circuit output needs to be further increased when miR$_{high}$ sensors are required for cell state identification, in particular if more than 1 output needs to be controlled. This could be achieved with extended combinatorial screening using an extended library of genetic parts [67] and new one-pot circuit optimization techniques such as 'poly-transfection' [75]. Further improvements can be achieved by optimizing the dynamics of the parts, such as delaying the production of Act2 with recombinase-based systems, which can remove leakage in transient systems [9, 47].

Fig. 7A. Is the circuit introduced in hiPS for functional gene expression been presented/characterized before in this paper? Hard to tell. This seems a circuit that does not discriminate anything in input, it only does the output control; right? If that's the case, how does it relate to the class of circuit that the authors have been talking so far in the paper?

We thank the reviewer for this comment. We realize that it is difficult to understand which circuit had been used for Figure 7 (NEW Figure 6). In the revised version, we separately describe the two circuits implemented: the generic circuit and minimal circuit. The minimal circuit that has been used for Figure 7 (NEW figure 6) uses an inverted logic compared to the generic circuit and is Off in hPSC. We highlight this circuit as an example of a circuit that requires no miR$_{high}$ sensor and exemplify how we created it empirically based on knowledge we acquired from the generic circuit implementation. Please refer to NEW Figure 1 for an overview of the circuits and workflow of the paper. The optimization of the minimal circuit, which was previously part of the optimization of the generic circuit (Prev. Figure 5D) is now highlighted in a separate figure (NEW Figure 5).

Please refer to page 15 for an introduction to the minimal circuit characterization section:

Page15: *"Thus far, we have demonstrated the rational model-guided design of generic circuits detecting the hPSC state (hPSC On). Because a miR_{high} sensor is not always required to discriminate desired cell states, we next used an empirical approach to create a minimal circuit that operates only with miR_{low} sensors. Specifically, we used the inverted logic to repress the output in hPSC state (hPSC Off). Such circuits can be useful to eliminate hPSC in hPSC-derived therapeutics or to avoid expression of effector molecules that are required to act at the later stages of hPSC differentiation."*

It is correct that we show the cell state control decoupled of the fine tuning in previous Figure 7 (NEW Figure 6). Thus, this figure validates the components of the full circuit separately in an actual output control scenario. Full circuit integration in a functional demonstration would be an important next step in our pipeline. We have provided text in the discussion to make this point clear (see Limitation section page 22 as copied above)

Fig. 7C what is in the y axis of the graph? Why is it so dramatically different between Sox2 and the other markers (4 orders of magnitude)?

Thank you for pointing this out. The 10E4 on the right Y Axis is a labeling mistake, which we corrected in the revised version (NEW Figure 6E). The scale is only 4x smaller. The Y-axis of the graph shows the fraction (or percentage) of cells that are Sox2+ positive (left Y axis) and TBXT or Sox17+ positive (right Y axis) from total live cells as measured by flow cytometry.

Fig. 7D/E What happens to the "black" regions in the circuit+, BMP4- conditions? Are there cells? Are cells alive/dead? Potentially interesting phenotype, lack of characterization makes it of questionable interest –

Thank you for the comment. We were wondering the same and investigated our images a little further.

To provide you with some context: these colonies are undergoing peri-gastrulation patterning triggered by the release of endogenous BMP4 by the cells. This patterning event entails 1) the emergence of the three germ-layer markers (i.e. SOX2, TBXT, and SOX17) in the colonies and, importantly, 2) change in the colony morphology from a 2D monolayer (pre-patterning) to multi-layered cell structure (post-patterning). The images showing the hPSC colonies represent the maximum intensity projection (MIP) for the various markers and DAPI.

The "black" regions in the images indeed contain cells but appear "black" due to the MIP algorithm used. For clarity, see the multiple z-stacks below for a circuit+, BMP4- colony where DAPI labeled cells can be observed in gray. We observe the highest density of cells at the lowest z-plane and fewest as we go higher in the z-plane. We are confident these cells were alive at the time of fixing, much like the cells that show the germ-layer markers. This can be inferred from the brightfield images below.

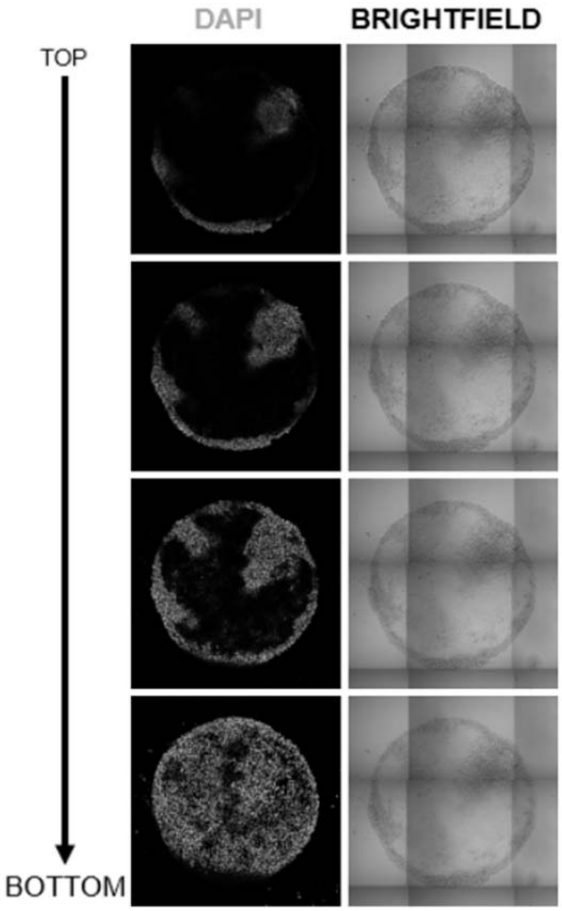

The figure shows multiple z-stacks of a circuit+, BMP4- colony at the end of the experiment. We note that there are more cells at the bottom of the colony than at the top. The cellular morphology can be observed in the brightfield panels. Cells at the centre of the bottom-most stack show non-apoptotic morphologies. These cells are more stretched vs the cells at the edge, which is likely an outcome of the spatially differential differentiation dynamics occurring in the colony.

Fig. 7E now checks a circuit with only input control (and discrete output=BMP4). Shows that the circuits with targeting vs non-targeting miRNA have the expected output and phenotypic effect regarding differentiation. These data do not show that one circuit is able to differentiate different cell states, but rather that the same cell state can be read differently by 2 circuits. To support a claim that a circuit is able to discriminate different cell states the right experimental setup seems (to me) show that the same circuit works differently when the cells are in 2 different states; e.g. if the authors differentiate those cells with the off circuit, do they start expressing BMP4? If the claim is that the functional circuits with input/output control can be implemented for affecting functional differentiation in hiPS, this needs to be shown. Or the claim can be something else.

We agree with this comment. As discussed above, in Figure 7 (NEW Figure 6) we have demonstrated different components of the full circuit independently, as a means to show that we can control physiological secreting effector molecules (BMP4) and with that can control differentiating cell compositions. Indeed, we only show repression of BMP4 in response the endogenous miRNA in hPSC and compare it to a mock circuit to simulate the On state. We agree that real cell state discrimination remains to be achieved but believe that showing the control of BMP4 in a mock cell state scenario is the

first key step towards this final pipeline and sufficient to provide evidence for the effectiveness of the circuits. To better reflect what we have achieved we have changed the claims to the following:

Page 16: *"First, we aimed to show that BMP4 production could be controlled in a discrete manner in response to endogenous miRNA inputs. For this, we used the best performing minimal hPSC Off circuit from Figure 5 (with tTA as Act2)."*

Middle of Page 17."In summary, our data indicate that the minimal version of a discrete-to-analog circuit". What is the circuit that the authors have in mind here? 7A? or 7E? Or a combination of the 2? The conclusions here combine together results from different circuits, making it sound that the authors think that combining the different parts will yield the final combined circuit. Is that the case? If so what makes them confident that it is the case (from my position, I don't see why that would be the case giving difficulties with combining circuits parts, as argued by the authors in the introduction; but maybe some of the results presented here may support that confidence that I am missing?)

We thank the reviewer for pointing this out. We agree, summarizing the sections with "a minimal version of the digital-to analog circuit" was misleading as we show the two functions separately. In the revised version we clearly introduce what is meant by the minimal circuit (NEW Figure 1) and how it is applied for BMP4 tuning (NEW Figure 6). Also, we removed all digital to analog terminology due to concerns from Reviewer#3.

Page 18: "*Additionally, BMP4 production can be effectively repressed in response to cell state-specific miRNAs, providing evidence that cell state-specific and fine-tuned control of secreting factors can be applied for control of cell compositions in differentiating hPSC*"

To clarify your questions: In NEW Figure 4, we show that adding the tuners to the output (and with that making the full circuit) is very straightforward as it requires only replacement of the output plasmid with one of the misFITS targeted plasmids. What is more challenging is to implement multi-component classifier circuits that incorporate several miRNA inputs, including miR_{high} sensors, while maintaining high levels of expression in the On-state. When On-state levels are low to begin with, the impact of tuning BMP4 expression down even further on differentiation is difficult to measure. Hence, we illustrated these two properties- tuning and state sensing, in two separate experiments.

MINOR POINTS

Page 5 bottom:
"In order to restrict circuit action to discrete cell states or cell types, a set of endogenous miRNAs must be identified to allow clear discrimination of the cell-state of interest (positive samples, here hPSC) from the other cell states (negative samples, here hPSC- derived differentiated cells) within a testing space." Not clear to me from what is presented here why it "must" be identified, or what kind of endogenous miRNAs set are we looking for here (cell-type specific?); consider rewording; maybe it is "the set of endogenous miRNAs that allow clear discrimination of cell states of interest must be identified"? Hard to parse as it is.

Thank you for the comments. We have modified the sentence to the following

*Page 6: "In order to restrict circuit action to discrete cell states or cell types, a set of cell type specific endogenous miRNAs **that can clearly discriminate the** cell-state of interest (here hPSC) and the other cell states within a testing space (hPSC- derived differentiated cells), require to be identified."*

Fig. 2

Figures 2A-B: It would be appreciated that the ordering of samples follows the intuitive course of differentiation, i.e. starting from Day0 on the left, and increasing incrementally until it reaches Day10 on the right.

We thank the reviewer for the comment. We hear your concern that was also raised by Reviewer #2. However, we think it is clearer to order the data the way we present it in Figure 2. The algorithm ranks the samples according to predicted circuit output production, which allows us to see which cell type can be easily discriminated from hPSC and which are more difficult to discriminate (the closer to hPSC, the less is the difference in output production to hPSC). This is the information we want to extract from the tool. In contrast, your proposed ranking would give us a nice overview on how miRNA expression evolves during differentiation. However, for our circuit design, we are not interested in the biological function of the miRNA, we only use miRNA as input cues for cell state identification and are looking for a way to compare the computationally predicted output production achieved in every cell state. Additionally, what you propose, ranking the data according to days of differentiation puts bias on the system based on biological pre-assumptions; naturally we assume that cells that are more differentiated from hPSC can be better discriminated against (and indeed we see this tendency in our data). However, because the miRNA identification algorithm uses an unbiased approach, it could also be (although unlikely) that the tool finds a miRNA profile that can discriminate early differentiated cells better than far differentiated cells. Indeed, in our data, we see that output production fluctuates during endoderm differentiation. This is why the data are not ordered from day 1- day10. If miRNA expression during endoderm differentiation is of interest for you, we would like to refer to the original paper where we derived the miRNA expression data from (Fogel et al, Gene 2015).

If the reviewer or editor still strongly feels this change is needed, we will do so.

Abbreviations for MPC, NPC, RNCS, EB should be provided in the figure legend. The MPC, NPC, RNCS, and EB datasets, while included in the analyses, are not mentioned in the main text; what purpose do they serve regarding hiPSC-specific miRNA identification on top of Day1-10 differentiation datasets?

We have provided the abbreviations for MPC, NPC, RNCS and EB in the Figure legend (NEW Figure 2A). The idea here was to include as many datasets as possible from hPSC and early hPSC-derived cell states. that are of sufficient in quality to serve as input into the search algorithm.

Figure 2B shows that miR-375 should supress the synthetic output when hiPSCs differentiate towards pancreatic cells or, at the very best, definitive endoderm. There are however no explorations or comments regarding how the output would be supressed in ectodermal and mesodermal lineages. Together with the fact that Figure 2 is purely computational without any experimental validation, these points raise questions regarding the circuit's performance.

We have experimentally characterized miR-375 suppression in hPSC, definitive endoderm and HEK293 (previous Figure 3B, NEW Figure 2C). Our data show that miR-375 significantly represses synthetic genes in definitive endoderm. In the revised version, to better connect the computational identification and experimental validations we have merged the two sections and provide all data together in NEW Figure 2. We hope this resolves your concerns.

It is correct that there is no data for ectoderm and mesoderm lineages. The reason for this is that we did not find miRNA expression data of hPSC-derived early ectoderm and mesoderm lineages in literature. With that we can only claim that the circuit is hPSC specific from cell types like MPC / NPC as demonstrated with HEK who have the same miRNA profile (Previous Figure 6, NEW Figure 4). The lack of ectoderm and mesoderm data was also the main reason why we apply only a mock ON/OFF performance for BMP4 control (previous Figure 7, NEW Figure 6). Despite this, we think that our paper serves as a valuable foundational pipeline platform that provides guidance to create desired circuits in hPSC and hPSC derived cell types.

In the graphs output (mol/cell) over Diff-hiPSC, there are always some point (cells I guess?) that give a high output for any circuit, for the non-hiPS cells; why? Is that a problem?

The data points with highest output are coming from EB, RNCS and day1 endoderm differentiated cells. These cell types show differentially expressed miRNA input compared to hPSC but because changes are small it does not result in full (or strong) repression of the output. Although the algorithm states a clear discrimination (FALSO or 0) of these cell types from hPSC, we think experimentally, in particular with a transient transfection setup, they might be difficult to discriminate. If this is a problem or not depends on the intended application. For this study it was not a problem, as we do not aim to discriminate hPSC from EB, RNCS or Day 1 differentiated cells.

I am not familiar with this kind of clustering and have had hard time finding quantitative definitions as well as high-level interpretative help for the following: AUC, Truth, Expr ratio over t(log2).

We have added the quantitative definition of the search tool outputs to the Methods section

Page 24: *"The definitions of the circuits and quantitative search tool outputs are the following:*

AUC: Area under the receiving operating characteristic curve. A measure to quantify the circuit output. It assesses circuit performance by scoring how well positive samples can be separated from negative samples.

cMargin: Classification margin. A circuit performance score assessing the ratio in output production between positive (here hPSC) and negative (here hPSC-derived cell states) samples. Specifically, the cMargin is the calculated average of the following two margins: the average margin (the overall ratio between outputs in positive versus negative sample sets) and the worst margin (the smallest ratio among any pair of positive and negative sample).

Truth: Binary truth value that annotates each sample that leads to output production above the classification margin as true (1) and below the classification margin as false (0).

Expression ratio over t: The ratio of expression above or below the binarization threshold t given as miRNA copies / cell. The binarization threshold t was set to 1 % of total miRNA abundance. Above this threshold a miRNA is considered to be biologically relevant (Farazi et al., 2011).

Pruning: a post-processing step using a bootstrap-based algorithm that prunes off miRNAs that do not significantly improve overall circuit performance

For more details on the model and definitions we would like to refer to Mohammadi et al. [39].”

Fig. 3
3B and C, would be informative I think to see histograms of DsRed alone ...

We agree that Histograms are informative and have added them to Figure 2D and Figure EV2.

in figures: what does T489, T375, ... mean? Not defined in fig nor in figure legend nor in text (that I can find)

We have added an explanation of T489 T375 to the Figure legend:

Figure 2C) Bar chart showing relative DsRed expression of sensors containing the indicated miRNA target sites recognizing the three identified miRNAs (T375, T489, T302a) and the misFITS miRNA (T17) as four fully complementary repeats (discrete miRNA sensing). The control vector (n.t.) does not contain any target sites.”

For Figure 3B and particularly 3C, was there any normalization applied to ensure that the test groups were equivalent in the stoichiometry of miRNA:construct, so that differences in the plotted results are rooted only in the variation of the miR-17 site sequence?

The data shown in Figure 3B and C (NEW Figure 2C and 2D) are normalized to Vector internal AmCyan control. This means that the targeted fluorescent reporter (DsRED) is placed on the same plasmid as the Fluorophore for normalization (the untargeted AmCyan). Both fluorescent reporters are controlled by the same promoter (TRE). Thus DsRED and Ctrl AmCyan have the same stoichiometry, allowing quantifying gene repression on single cell level. Because of this internal normalization, we can accurately capture the differences in repression when cloning different miR-17 target sites. For more information we would to refer to Michaels et al, Nat. Commun., 2019. We have to note however, that the values calculated are only relative and potentially underestimate the actual repression, in particular in cells that receive high copy number of plasmids. This happens because the expression of the two fluorescent reporters are competing for cellular resources.

in figure3C: what does 3-C mean?

This is the name and description of one of the variants. 3-C means that within the original fully complementary miR-17 target site (T17), the nucleotide at position 3 has been replaced with a C. Please refer to Appendix Table S1 for description and full sequence of the variant target sites. For more information on the misFITS, we refer to Michaels et al, Nat. Commun., 2019.

We have described this more clearly in the legend of Figure 3C (NEW Figure 2D). “Each target variant is labeled with the mutated nucleotide as listed in Table S1.”

When testing the analog miR-17 variants in hiPSCs, the authors comment "Compared to the HES-2 line, the H1 line showed significantly higher DsRed expression for most sensors". This is interesting and

deserves some elaboration, even if hypothetical: what is the reason, according to the authors, that different hiPSC lines have different miRNA activity/profiles? Would this line-to-line variation compromise the circuit's reliability in performance?

The heterogeneity between different hiPSC lines has been described (Harrison et al, Stem Cells, 2010). Differences in dsRED expression could be due to small differences in miRNA expression between cell lines. Given our in-depth characterization of circuit performance as a function of component dose, these differences do not compromise circuit performance; small changes in design could be useful for second generation optimization for each line.

Fig.4

"Act1 performs similarly to the model presented in Schreiber et al, achieving a high On/Off ratio whenever KD or Act1MAX is not particularly low". This seems counter-intuitive to me: wouldn't a low KD indicate high-affinity of Act1 for Pind1, therefore sensitivity of Pind1 to Act1, and a high or sudden On/Off ratio in response to increases in Act1 levels?

Not necessarily. Although, as you outlined, a low KD can lead to strong induction of FF4 which is beneficial to achieve a high On / Off ratio, a low KD can also mean that, repression of Act1 by endogenous miRNA requires to be extremely strong, else FF4 can still be induced resulting in a lower On state. Thus, to achieve optimal On/Off ratio finding the best combination of parameters is required.

"Third, we chose a relatively weak promoter (UBC [52]) to express Act2 to keep Act1MAX levels low (Figures 4 and S3)" do the authors mean Act2MAX instead of Act1MAX?

Excellent catch. Yes, we meant Act2MAX. Thank you for pointing this out. This has been corrected.

Figure 4B, plot showing On:Off ratio vs MiR-FF4MAX concentration is interesting: firstly, it seems to be following Michaelis-Menten instead of Hill kinetics. Secondly, what practical information does the simulation give; e.g., what is the concentration or amount of miRNA cells should express for the circuit to function with a strong On:Off response?

To the first comment: Michaelis-Menten model is a special case of Hill's model. MM is equal to Hill model, when Hill coefficient is n=1. To the second: We would like to refer to Appendix Figure S3, where we analyzed the output production in response to changing miRNA input concentration. A miRNA concentration between 10E2 and 10E4 mols / cell will lead to high output repression depending on what circuit parameters have been chosen (Appendix Figure S1).

Fig. 5

Figure 5A, 5E: Why are the miRlow sensors placed at the 3'UTR of Act2 and not the output?

Thank you for asking this important question. If we would place the miRNA low sensors in the output we would not be able to tune the output with misFITs as desired, as both the inputs cues (in a digital manner) and the miSFITs (in a fine-tuned manner) are targeting and regulating the output. Therefore,

we decouple input detection (or cell state identification) from output control using a bow-tie architecture. This approach increases modularity both 1) technically because output constructs do not need to be re-engineered and 2) from a gene regulatory perspective because the output production can be better predicted as changing miRNA inputs merge as a logic gate on the central knot (Act2) of the circuit and does no affect the output production directly.

Why is the best FF4 Off/On performance at low Act1MAX amounts (Figure 5B)? Is it the same reason as the output performing optimally with low Act2MAX (so that they are completely covered by the miRNAs)?

This is a good question. Yes, low Act1MAX means it is easier to achieve full repression with physiological concentration of the underlying targeted miRNA.

"We conclude that FF4 production is not high enough in most of the combinations to sufficiently repress Act2 in the PIT2-tTA circuit leading to unexpected circuit behaviour for this configuration (Figures 5C and S5A)" how are FF4 and Act2 quantified and compared stoichiometrically to reach this strong conclusion?

FF4 production is approximated by mCitrine production because FF4 is spliced intronically from mCitrine. Because FF4 is targeting Act2, the assumption is that the more FF4 (and the more mCitrine), the less Act2 is produced. In the PIT2-tTA circuit, FF4/mCitrine is lower and Output production is higher than in the rtTA-PIT2 circuit. This makes sense because we know from titration experiments of the separate modules that rtTA produces more FF4/mCitrine than PIT2, and tTA produces more output than PIT2 (see Figure EV3). As suggested by our mathematical model, a combination of low FF4 with high Ouptut production as shown in the PIT2-tTA circuit is unlikely to work.

We understand that the sentence has not been formulated clearly and thus we have modified the sentence to the following:

*Page 11,12: " The unexpected circuit behaviour of PIT2-tTA, a*s suggested by our mathematical model, results from an unfavorable combination of low FF4/mCitrine (low FF4MAX) and high Output production (due to low KD2 or high Act2MAX) (NEW Figure 3C, Appendix Figure S5A)"

The trade-off between maximum expression levels vs maximum inducibility of the miRNA circuits reflects that of mammalian synthetic promoters (Ede et al 2016, ACS Synth Biol.). Seems could be great to hear the authors elaborate on this.

Yes, the Max protein production of each construct is determined by the strength of the constitutive promoter or the "inducibility" of the inducible promoters. Here, we have tested two constitutive promoters (EF1a and UBC) and use inducible promoters that have been optimized for leakiness and dynamic range (Angelici et al, Cell Reports, 2016). The mathematical model we developed describes these dependencies on expression and repression (see Material and Methods).

Figure 6D: Addition of scrambled miR-Ctrl in "HEK-293 Ciruit 1" seems to slightly increase mCherry output. Also, in "H1 Circuit 1", addition of miR375 or miR489 seem to reduce mCitrine levels, which is unexpected given these miRNAs regulate UBC::PIT2 which lies downstream of the TRE::miRFF4-mCitrine module?

We thank the reviewer for highlighting this. Related to miR-Ctrl increasing mCherry output: sometimes we see that addition of additional genetic material boosts transfection. An alternative explanation is that miRCtrl acts as an inducing miRNA for the miRhigh sensor due to nonspecific binding. As for the miR-375 and miR-489 affecting mCitrine: we have to note that mCItrine expression is already very low without miR-375 and miR-489. So, what we see in the chart may as well be noise. It is hard to quantify as very few cells are mCitrine positive. Thus we would not give too much weight on these effects.

Bottom of page 12: typo (I think): (Figure 6B should read Figure 6C);
Thank you for highlighting this typo. This has been corrected (using the new Figure numbering).

fig. 6C, what is on X axis of mCitrine graphs? If it is circuits, where is circuit 3? Zero/invisible?

Correct, circuit 3 contains no mCitrine as illustrated in the Figure 6A (NEW Figure 4A). So, it is Zero and below detection limit. To make this clearer we have added a description (b.d. = below detection limit) to the data point in the figure and updated the Figure legend.

"Because the highest On state is already weak, by further tuning the levels down, we observed not only a shift in the mean value of the expression intensity but also in the number of cells that scored as mCherry positive". This would seem to suggest a problem in using the analog miR-17 tuner in hiPSCs: fine-tuning makes sense when we have a substantial amount of gain already, and we'd like to perform some fine adjustments; when the output is overall low, the primary aim is to add gain, then do the fine-tuning. Having said that, normalized data in Fig6E look great.

Yes, we agree with this statement. However, we want to add that only the circuit with miR_{high} sensor leads to very low output and therefore we suggest further improvements if miR_{high} sensors are required for cell state discrimination (see Limitation Section in Discussion). We also want to note that this is a problem that arises due to transient transfection setup which introduces considerable copy-number and expression heterogeneity. It is well possible that with stable integration of the current circuits, the output production could be high enough to exceed optimal physiological concentrations and thus benefit from tuning. Additionally, we show with the BMP4 example (NEW Figure 6) that when using a minimal circuit, even a transient transfection with a relatively low number of cells receiving all circuit components can produce desired physiological effects.

Fig.7

Curiosity: S7B shows what happens to the cells producing BMP4: any reasons why that may be the case? Where are these cells on the discs? Where is mCitrine in the circuit? (For interpretation of Fig. S7)

First, mCitrine does not come from the circuit, it is co-expressed with Sox2+ in the genome-edited cell line (RUES2). Thus, mCitrine signal represents an approximation for Sox2 expression.

Second, these cells are undergoing peri-gastrulation patterning: the process that results in the emergence of the three germ layers (i.e. SOX2, TBXT, and SOX17) due to colony exposure to BMP4. Previously this was achieved by exposing the micropatterned hPSC colonies to media containing BMP4 (i.e. homogeneous exposure to BMP4) (Warmflash et al., Nat. Methods, 2014, Tewary et al., Development, 2017). Consequently, cells in the colony lost their pluripotency markers (i.e. SOX2-OCT4++) to display markers linked to the three germ-layer fates: ectoderm-associated (SOX2), mesoderm-associated (TBXT), and endoderm-associated (SOX17). Our group has previously shown that this patterning, especially the emergence of TBXT+ and SOX17+ cells, is dependent on [BMP4] (Tewary et al., Development, 2017) as well as proposed a functional GRN circuitry mediating the emergence of these germ-layer markers (Kaul et al., bioRxiv, 2020). Prev. Figure S7 and new Figure EV5 confirms these colonies are undergoing peri-gastrulation patterning, due to the emergence of TBXT+ and SOX17+ cells following the introduction of the gene circuits in these cells. Further, this figure shows that circuits yielding lower BMP4 release result in fewer TBXT+ and SOX17+ cells, whereas the circuits yielding higher amounts of BMP4 yields more TBXT+ and SOX17+ cells illustrating that [BMP4] is directly linked to the extent of peri-gastrulation patterning even when BMP4 is being released by individual cells (as opposed to homogeneous exposure to BMP4).

Reviewer #2:

The authors engineer genetic circuits with sense-and-respond capabilities to activate expression of a desired protein in a particular cell state. Here, they utilize the cell state-specific expression of microRNAs (miRNAs) to distinguish undifferentiated human pluripotent stem cells (hPSCs) from other, differentiated cell types. They develop an automated way of analyzing omics datasets to identify miRNAs that are specifically over-expressed or depleted in cell states, followed by utilizing those miRNAs to either repress or de-repress transcription factor expression (here, Act1 or Act2). The miRNA-dependent expression of the TFs is used to classify whether the cell is in the hPSC state. The authors then utilize the Act1 and Act2 transcription factors (activators) to regulate the expression of either a fluorescent protein reporter (an observable proxy) or the BMP4 morphogen. Their best genetic circuits are able to activate output protein expression by up to 7.7-fold for a relevant effector protein (BMP4).

The authors expand on an existing model of miR-dependent and TF-dependent transcriptional regulation to guide the design of their genetic circuit. With the model, they identified the key parameters that affect circuit function. They also leveraged the model to explain why the dynamic range of the circuit is lower than desired when using a miR with a high expression level in the hPSC cell state (miR high sensor).

Overall, the manuscript is clearly written, though quite lengthy. It was particularly important that each section ends with a brief summary of the results. The following comments will improve the readability of the manuscript.

We thank the reviewer for the positive feedback. We hope the revised paper, which has been streamlined and edited for clarity, improves the readability of the manuscript.

Specific Comments

Several changes to the figures will improve clarity:

1. In Figure 1, the blue box in the circuit schematic is unlabeled. Presumably, it is the Act1 or Act2 transcription factor, but it needs to be labeled. It's also important to explicitly label the miR binding sites and the Act binding sites. The red box in the genetic circuit diagram is also unlabeled. It is not clear what the red box indicates; is it a reporter protein, the output protein (e.g. BMP4), or the "miSFIT" variants? The mechanism of the miSFIT variants are not well-described. Even though their development is from a prior paper, Figure 1 needs to clearly show that other constitutively expressed miRs are being used to tune the expression of the output through design of miR binding sites in the 3' UTR.

We thank the reviewer for the useful comments. We have changed Figure 1 to better describe the content of the paper. We added the missing descriptions to the new Figure 1. We also incorporated additional descriptions on the miSFIT variants in the text.

Page 6: "*To fine-tune the expression levels of each output, we applied miSFITs [45], a previously reported approach that operates on a library of mutated target sites for miRNA-17. miR-17 is ubiquitously and strongly expressed among most human cell types, including hPSC [48]. To tune protein outputs, one repeat of a miRNA-17 target site variant was placed in the 3'UTR of the protein outputs (Figure 1C). Each variant contains different mutations in the target site leading to reduced binding strength of endogenous miR-17 and thus reduced repression. The decrease in repression strength depends on the position and identity of the mismatched nucleotides [45]. Thus, by selecting a desired mutant variant from the miSFITs library, expression of the output proteins can be tuned to desired levels [45].*"

2. In all figures, the word "predicted" or "calculated" needs to be used to denote modeling results. For example, in Figure 2, it is not readily apparent that the middle panels are modeling calculations. Same for Figure 4B and Figure 5B.

We agree this is useful terminology. We have added predicted or calculated to the following sentences:

Related to NEW Figure 3

Page 9: " First, the model predicted that increasing miR-FF4^{MAX} as well increasing Act1^{MAX} concentration leads to higher dynamic range"

Page 10*: "Second, the model predicted that increasing miR-FF4 mediated repression of.."*

Page 10: " Third, the model predicted that the dynamic range is higher at low Act2^{MAX} concentrations."

Page 10:*: "Further, the model predicted that increasing inhibition of Act1 by miR-302a, …"*

Page 10:" Mathematical modeling of such a screen predicted that…"

Page 11: "The model further predicted that Output production.."

Page 11: "Thus, the unexpected circuit behaviour of PIT2-tTA, as predicted by our mathematical model,.."

3. In Figure 2B, it is not clear why the miR-375 expression ratios are sorted (descending). They should be sorted by progression over Days. Otherwise, it is confusing and potentially misleading.

We thank you for the comment. Reviewer #1 had a similar concern, however, in our opinion, arranging the data so the output production is ranked from high to low makes more sense. The reasons are the following:

The algorithm ranks the samples according to predicted circuit output production, which allows us to see which cell type can be easily discriminated from hPSC and which are more difficult to discriminate (the closer to hPSC, the less is the difference in output production to hPSC). This is the information we want to extract from the tool. In contrast, your proposed ranking would give us a nice overview on how miRNA expression evolves during differentiation. However, for our circuit design, we are not interested in the biological function of the miRNA, we only use miRNA as input cues for cell state identification and are looking for a way to compare the computationally predicted circuit output production achieved in every cell state. Additionally, what you propose, ranking the data according to days of differentiation

puts bias on the system based on biological pre-assumptions; naturally we assume that cells that are more differentiated from hPSC can be better discriminated against (and indeed we see this tendency in our data). However, because the miRNA identification algorithm uses an unbiased approach, it could also be (although unlikely) that the tool finds a miRNA profile that can discriminate early differentiated cells better than far differentiated cells. Indeed, in our data, we see that output production fluctuates during endoderm differentiation. This is why the data are not ordered from day 1- day10. If miRNA expression during endoderm differentiation is of interest for you, we would like to refer to the original paper where we derived the miRNA expression data from (Fogel et al, Gene 2015).

If the reviewer still strongly feels this change is needed, we will do so.

4. The measurement scales in Figure 5C and 5D vary greatly across the experiments, leading to potentially misleading color schemes. For example, for the PIT2-rTA circuit, the "Off" state varies from 0.3 to 0.7 for the mCitrine measurement and from 0 to 0.2 for the mCherry measurement. Even though mCitrine and mCherry measurements exist on different scales, the lower limit should always be zero. Further, it's not clear at all if the difference between 0 and 0.2 is statistically significant or whether it's all "zero" within noise. Unlike the authors use of bar plots in other panels, there are no error bars in Figure 5CD, which makes it impossible to determine significance.

We thank the reviewer for pointing this out. The scales have been chosen to better visualize the changes using colormaps. To address your concerns, we have added the barcharts that are associated with Figure 5C (NEW Figure 3C) to Figure EV4C. We also provide the Source Data of this Figure. We clearly see significant differences in mCherry as we change Act2 dose. We want to note that although mCherry is expression is weak for the circuits the values are artificially low because of normalization with internal transfection control that uses a very strong promoter (Ef1a).

Typos:

"The here reported designs, plasmid libraries and optimization approach provides" should be "Here, the reported designs, plasmid libraries and optimization approach provide"
Figure 5F. "senor" should be "sensor"

We have corrected the typos and thank the reviewer for pointing them out.

Reviewer #3:

This manuscript aims to develop gene circuits that sense and distinguish the stem cell state from the differentiated cell state and mount appropriate responses. Two genetic modules are designed to achieve this aim. The input module consists of sensors of high and low microRNA (miR) levels, which converge onto an AND gate to define cell state based on the presence and absence of various miRs. Here, computational algorithms identify the absence of miR-489 and miR-375, combined with the presence of miR-302 as a signature of human pluripotent stem cells (hPSCs). The output module can be off or on, according to the input module's decision. The output level is adjustable by using various numbers and sequence variants of the native miR-17 target site. Various circuit configurations are considered and optimized using a computational model introduced earlier (Ref. 46). Following the optimal design, circuit function is demonstrated in hPSC versus other cell lines, and applied to control endogenous BMP4 secretion in culture, which can affect cell composition and morphogenesis.

This is an interesting, application-oriented interdisciplinary study that is carefully executed and presented. Considering the potential for future applications, the manuscript is relevant to a diverse audience. However, certain parts and terminology are unclear and there are some concerns about the experimental and computational methodology and their interpretation. Therefore, the following comments should be addressed before the manuscript is suitable for publication in Molecular Systems Biology.

We thank the reviewer for the great summary and positive feedback.

(1) The system is designed and tested using transient transfection with plasmids. This does not become clear until later in the manuscript, so it should be specified early in the Results. While it facilitates experiments, this methodology has multiple shortcomings: plasmids are taken up into cells and then lost, leading to temporal up- and downswings of plasmid copy numbers that need to be studied before precise implementation; various copies or no plasmids are taken up by different cells, causing drastic cell-cell variability that severely compromises the cell-to-cell reproducibility of circuit responses even after gating out cells without plasmids. All these effects and their effects on circuit function should be acknowledged and either studied at the single cell level to properly define the capabilities of the system, or their omission should be carefully justified and discussed. The possibility of integration, and its pluses and minuses should be discussed.

We thank the reviewer for this comment. We have added descriptions on the transient transfection methodology to the introduction paragraph:

Page 4 "*Specifically, we provide a platform for engineering of gene circuits using transient transfections*"

We agree that transient transfection has shortcomings but think that the benefits largely outweigh them in the context of characterizing new circuit functions in human cells. Indeed, transient transfections lead to high cell to cell variability, however, we see this as an advantage for circuit development and characterization, because it enables validation that circuits work robustly across a range of copy numbers. This is especially relevant for cell state classifiers, which are most robust if they achieve output repression in Off states across a wide range of circuit copy numbers.

Also, we agree that due to the heterogeneity and dynamic instability of transient transfection, it is not a good methodology for all applications, in particular within differentiating hPSC cultures that are intended to be grown for extended periods of time and hence require stable integration of the circuit. miR_{high} sensors in particular encounter difficulties in transient transfections due to unfavorable dynamics of circuit component expression (Lapique et al, Nat. Biotec., 2008). However, certain applications are feasible with transient transfections. As demonstrated here, if a minimal circuit can be applied (without miR_{high} sensor), we found that secretion of effector proteins such as BMP4 can achieve desired effects (here differentiation) without requiring homogenous expression.

Unfortunately, reliable performance of stably-integrated circuits composed of multiple genes remains a major challenge. Reasons include cross-reaction between circuit elements, silencing and inefficient integration of large genetic elements. Therefore, stable integration is typically out of scope for early proof-of-concept implementation such as in this study.

To sum up, we believe transient transfection is an excellent methodology to demonstrate and characterize new circuit functions in human cells but falls short in reflecting the performance of stably-integrated circuits or applications in changing / differentiating hPSC cultures, which are significant areas for future research and optimization.

We have added a statement to summarize these points in the Limitation section

Page 22: "*Third, the current transient transfection setup has several shortcomings, including high cell-to cell variability and loss of plasmids over time, therefore limiting their applications. Stable integration of circuits of this complexity have proven challenging due to the low integration efficiency in hPSCs and because, once integrated, circuit silencing and unexpected cross-activation from genome to the circuit or within circuit components can occur [14, 69].*"

(2) According to the previous point, appropriate mathematical models are needed. To capture temporal changes in plasmid copy numbers and RNA/protein levels, time-dependent ordinary differential equations (ODEs) are needed. To capture cell-cell differences in plasmid copy numbers and RNA/protein levels, stochastic models are needed. As opposed to this, modeling in this manuscript is based on Schreiber et al. (46), which consists of a simplistic set of non-cooperative Hill function dependencies describing all interactions (transcription factor and miR effects), incapable of capturing either temporal changes or cell-cell differences. The description of the model in the Results should be clearer. Alternative modeling approaches should be considered, or their omission should be carefully justified and discussed.

We thank the reviewer for raising this important comment. We agree that ODE models are fundamentally important to describe, learn from and further improve circuit performance. In fact kinetic models of the classifier circuit (Xie et al.,Science, 2010) and bow-tie circuit (Haefliger et al., Nat.Commun. 2018), as well as new bin-based ODE models designed for transient transfections (Stelzer, PLOS Comp. Biol. 2020) have been created and applied by our team to improve our understanding of classifier circuits. Therefore, we did not feel the need to study the dynamics and stochastic behavior further in the current study. Instead, we believe that a simple steady-state model is appropriate to make useful predictions about circuit function across important experimentally-tunable parameters (as proposed by Schreiber et al. Mol Syst.Bio, 2016) and see this is a straightforward approach for circuit

optimization to different cell types and miRNA inputs and hope our approach can serve as a guide for researchers who want to create their own circuits with limited computational experience.

We agree that model description and presentation of data were not clear. In the revised version, we have improved the results section describing the model and associated data and restructured the Figures. Please see new Figure 3 and related results section on page 9-12.

Additionally, we have added the following sentence to the discussion to acknowledge the limitation of our modeling approach and guide the reader to other places where more complex models have been used.

Page 25 (Discussion) *"However, we would like to note that the model used herein does not capture inter- and intra cellular variabilities and expression dynamics. To gain a more accurate understanding of the circuit, stochastic ODE models [65, 66] or bin-dependent ODE models designed for transient transfections [67, 68] are recommended. For deterministic ODE models of the bow-tie circuit, we would like to refer to Haefliger et al [41].."*

(3) Using the term "digital-to-analog" in the title and manuscript is unjustified and misleading. Digital-to-analog conversion in electronics refers to a single device (such as a single modem or CD player) producing an analog signal from a digital one. In contrast, here the digital signal of the input module remains fully digital at the output for each individual cell line. An individual miR-17 site variant in the output module sets the mean level of the ON state, but the output remains completely digital: the output simply reproduces the AND gate's digital outcome at some preset amplitude, without any analog conversion. For truly analog conversion, time-dependent continuous signals such as sine waves, sinc or Gaussian pulses would be needed at the output, which does not seem to be the case. The terminology should be revised, and "digital-to-analog" claims should be removed from the title and throughout the manuscript.

We thank the reviewer for this comment. We had a lot of internal discussion about the terminology and agree that an alternative terminology for the paper and circuit is more appropriate.

We have changed the Title to:

"Synthetic gene circuits for cell state detection and protein tuning in human pluripotent stem cells"

Additionally, we have removed all digital-to-analog circuit terminology.

(4) The claim that miR-17 target site-based miSFITs "tune expression at very fine levels" is not justified. Although it may be true that miSFITs tune the *mean* expression level, but expression in any clonal cell population is much more than its mean: it is a distribution, which has a standard deviation, a skewness, a kurtosis and so on. To claim "very fine-level tuning", histograms should be plotted in every figure with scatter plots, and at least the standard deviation, or the coefficient of variation (CV) should be studied. Scatter plots as in Figure 3B,C, or Figure 6C,E are insufficient: These scatter plots should be converted into histograms of a single fluorescent channel, and the shape and CV of such histograms should be studied. In fact, some histograms in Figure 3C (3-C, 9-G) indicate bimodality, which not only directly contradicts expression tuning, but also obviates even the possibility of analog signals at the single cell

level. For fine-level expression tuning in mammalian cells, a relevant reference that would be worth citing is PMID:32167761. This system may be noisy due to ultrasensitivity of responses and sequential cascades, see PMID:12149512 and PMID:15738412.

The heterogeneity in fluorescence observed across the cell population is largely due to the nature of transient transfection. Specifically, lipid-based transfection reagents lead to multiple orders of magnitude variation in transfected plasmid copy number across the population. To correct for this copy number heterogeneity, we normalize the miSFIT-controlled DsRed expression levels to our internal AmCyan control reporter. When we perform this normalization on a per-cell basis and plot histograms of these normalized DsRed values, we see that the shift in mean expression is now much larger relative to the coefficient of variation (the distance between peaks is now much larger relative to the peak widths). This visualization also confirms that, when gating only the transfected population and normalizing to the internal control, DsRed expression is generally uni-modal rather than bi-modal. This normalization gives a good indication of the degree of tuning that could be expected for defined copy-number integration of our circuits which could be achieved using low MOI lentiviral transduction or precise genomic knock-in. Indeed, when mISFITs were previously used with lentiviral vectors, we did observe small CVs relative to the shift in population means (See Michaels et al 2019, Supplementary Figure 5c). Thus, we feel justified in describing this as fine-tuning.

We have added histograms to previous Figure 3 (NEW Figure 2D) and Figure EV2F.

(5) What sort of mean was used to create bar graphs from fluorescence data? The arithmetic mean of log-normally distributed or highly skewed data will be biased towards higher values. Either the geometric mean of untransformed data or the arithmetic means of log-transformed data would be a proper representation.

We have used the arithmetic mean. We agree that geometric mean is a less biased quantification of log-normally distributed data. However, we have two reasons why we feel the arithmetic mean is justified here:

First, we don't interpret our data solely from the arithmetic mean. Instead, we use a different metric to represent the performance. Our data include the cell frequency and are always normalized to the internal transfection control. Because the arithmetic mean of the internal transfection control is also biased, we typically observe very similar trends in normalized data regardless of the mean used.

Second, the metric we are using here has historically been used to describe similar circuits (e.g. Xie et al, Science 2010, Prochazka et al, Nat.Commun, 2016, Haefliger et al Nat.Commun 2020) and would prefer our measurements to be comparable to prior work.

(6) Multiplication by cell frequency is used to balance the cell count in each channel, but the final metric will be sensitive to variations in plasmid copy numbers if parts of the circuit are distributed to different co-transfected plasmids. The individual plasmid maps with circuit components they contain (and sequences as Supporting Data) would be highly helpful and should be provided. A possible issue is the lack of technical control where the cascaded elements of the circuit are decoupled by individual plasmid transfection (to get separate baseline intensities for each cascade level in the final circuit). Taking Figure 6, the relation between iRFP and mCitrine is highly nonlinear. If multiple plasmids were used, the

analysis might be dominated by cell-to-cell variability in transfection and copy numbers of individual plasmid uptake, making the results hard to interpret, especially without controls.

We thank the reviewer for this useful feedback and suggestions. We have created a folder with all plasmid maps that will be provided with the manuscript (Dataset EV1) and initiated a submission to Addgene. Please refer to Appendix Table S2 for list of plasmid.

It is true that there is significant nonlinearity between iRFP and mCitrine, and even more between iRFP and mCherry. This is due in part both to intentional differences in the relative amounts of each plasmid delivered and the digitizing nature of transcriptional cascades. As an example of the former, the circuit data depicted in NEW Figure 4 use a plasmid ratio of 0.1-1-0.3-1 (Act1-mCitrine/FF4-Act2-mCherry/Out) and the transfection control iRFP at 1. Co-transfected plasmids are delivered to human cells with very high correlation; though at low copy numbers there is more stochasticity, especially when the relative amount of a plasmid is reduced (Gam et al., Nucleic Acids Res.,2019). In our system, mCitrine/FF4+ and mCherry/Output+ cells have medium to high circuit copy numbers (as indicated by iRFP fluorescence), and as such we expect such stochasticity to have a relatively minor effect on cell-cell variation. More substantially, longer cascades impart increasingly stochastic and non-linear dose-responses between inputs and outputs (Hooshangi et al., Proc Natl Acad Sci USA, 2005). Indeed, we can see switch-like activation of mCherry and mCitrine as a function of circuit copy number (which correlates with expression of their activators Act1 and Act2, respectively). As expected for a multi-stage cascade, activation of mCherry/Output is nearly digital once circuit copy number/Act2 expression is sufficiently high.

We agree with the reviewer that output readouts by themselves can be difficult to interpret. This is why we only interpret our data as relative performance to control circuits. Specifically, we normalize our data to internal transfection control, which allows us to normalize for transfection to transfection variations. Additionally, we always compare circuit performance to that of carefully designed control circuits. For example, in NEW Figure 4, we designed control circuits to measure the maximal mCitine/FF4 production and output production respectively. Thus, our approach in this paper, for characterization purposes, is to give a measure of relative performance on population level. We agree that for certain applications, either stable integration at defined copy numbers or a much more rigorous single cell characterization might be required.

(7) Some trends in the figures (such as the downward trend of On levels in Figure S4) are difficult to understand and require better explanation.

Thank you for this suggestion - the text has been reviewed and edited to align the text with the data in the figures.

Page 11|: *Note, we observed strong inhibition of FF4 / mCitrine expression at high doses of Act1 in the Off state when using rtTA as Act1 (Figures EV3A and EV4B), due to cellular resource competition [54, 55].*

Page 21: *Our data also revealed that precise tuning of two or more output genes using plasmid transfection is challenging because of cellular and circuit-based resource competition. Recent studies characterized how synthetic genes compete with each other for cellular resources in human cells, with both computational models to capture and practical solutions to dampen this competition effect [54, 55, 64]. Our results are consistent with a previous report of translational-level resource redistribution by*

miRNAs, where miRNA suppression of one protein's translation increases production of other proteins [54]. Future efforts to mitigate or accurately predict cellular resource competition will help improve overall circuit performance and the utility of miSFITs for tuning multiple genes independently.

1st Revision - Editorial Decision 14th Sep 2022

Thank you for sending us your revised manuscript. We have now heard back from the two reviewers who agreed to evaluate your study. As you will see, the reviewers are overall satisfied with the modifications made.

Before we can formally accept your manuscript, please address the following issues:

1. the remaining minor suggestion from Reviewer #3.

on a more editorial level:

Reviewer #2:

The authors have responded well to the reviewers' comments by considerably restructuring their manuscript to improve its readability. They have also addressed my concerns regarding the way the data was previously plotted.

Reviewer #3:

I would like to thank the Authors for addressing my questions. I would like to recommend the manuscript for publication, pending a small modification. When referring to precise tuning by stably integrated gene circuits, even though it was not done in hPSCs, PMID:32167761 would be worth citing.

Thank you again for sending us your revised manuscript. We are now satisfied with the modifications made and I am pleased to inform you that your paper has been accepted for publication.

EMBO Press Author Checklist

Corresponding Author Name: Peter W. Zandstra
Journal Submitted to: Molecular Systems Biology
Manuscript Number: MSB-2021-10886R

USEFUL LINKS FOR COMPLETING THIS FORM
The EMBO Journal - Author Guidelines
EMBO Reports - Author Guidelines
Molecular Systems Biology - Author Guidelines
EMBO Molecular Medicine - Author Guidelines

Reporting Checklist for Life Science Articles (updated January
This checklist is adapted from Materials Design Analysis Reporting (MDAR) Checklist for Authors. MDAR establishes a minimum set of requirements in transparent reporting in the life sciences (see Statement of Task: 10.31222/osf.io/9sm4x). Please follow the journal's guidelines in preparing your manuscript.
Please note that a copy of this checklist will be published alongside your article.

Abridged guidelines for figures
1. Data
The data shown in figures should satisfy the following conditions:
- the data were obtained and processed according to the field's best practice and are presented to reflect the results of the experiments in an accurate and unbiased manner.
- ideally, figure panels should include only measurements that are directly comparable to each other and obtained with the same assay.
- plots include clearly labeled error bars for independent experiments and sample sizes. Unless justified, error bars should not be shown for technical
- if n<5, the individual data points from each experiment should be plotted. Any statistical test employed should be justified.
- Source Data should be included to report the data underlying figures according to the guidelines set out in the authorship guidelines on Data

2. Captions
Each figure caption should contain the following information, for each panel where they are relevant:
- a specification of the experimental system investigated (eg cell line, species name).
- the assay(s) and method(s) used to carry out the reported observations and measurements.
- an explicit mention of the biological and chemical entity(ies) that are being measured.
- an explicit mention of the biological and chemical entity(ies) that are altered/varied/perturbed in a controlled manner.
- the exact sample size (n) for each experimental group/condition, given as a number, not a range;
- a description of the sample collection allowing the reader to understand whether the samples represent technical or biological replicates (including how many animals, litters, cultures, etc.).
- a statement of how many times the experiment shown was independently replicated in the laboratory.
- definitions of statistical methods and measures:
  - common tests, such as t-test (please specify whether paired vs. unpaired), simple χ2 tests, Wilcoxon and Mann-Whitney tests, can be unambiguously identified by name only, but more complex techniques should be described in the methods section;
  - are tests one-sided or two-sided?
  - are there adjustments for multiple comparisons?
  - exact statistical test results, e.g., P values = x but not P values < x;
  - definition of 'center values' as median or average;
  - definition of error bars as s.d. or s.e.m.

Please complete ALL of the questions below.
Select "Not Applicable" only when the requested information is not relevant for your study.

Materials

Newly Created Materials	Information included in the manuscript?	In which section is the information available? (Reagents and Tools Table, Materials and Methods, Figures, Data Availability Section)
New materials and reagents need to be available; do any restrictions apply?	Yes	Data Availability Section, Appendix Table S2

Antibodies	Information included in the manuscript?	In which section is the information available? (Reagents and Tools Table, Materials and Methods, Figures, Data Availability Section)
For **antibodies** provide the following information:<br>- Commercial antibodies: RRID (if possible) or supplier name, catalogue number and or/clone number<br>- Non-commercial: RRID or citation	Yes	Material and Methods

DNA and RNA sequences	Information included in the manuscript?	In which section is the information available? (Reagents and Tools Table, Materials and Methods, Figures, Data Availability Section)
**Short novel DNA or RNA including primers, probes**: provide the sequences.	Yes	Appendix Table S1

Cell materials	Information included in the manuscript?	In which section is the information available? (Reagents and Tools Table, Materials and Methods, Figures, Data Availability Section)
**Cell lines:** Provide species information, strain. Provide accession number in repository **OR** supplier name, catalog number, clone number, and/**OR** RRID.	Yes	Material and Methods
**Primary cultures:** Provide species, strain, sex of origin, genetic modification status.	Not Applicable	
Report if the cell lines were recently **authenticated** (e.g., by STR profiling) and tested for mycoplasma contamination.	Yes	Material and Methods

Experimental animals	Information included in the manuscript?	In which section is the information available? (Reagents and Tools Table, Materials and Methods, Figures, Data Availability Section)
**Laboratory animals or Model organisms:** Provide species, strain, sex, age, genetic modification status. Provide accession number in repository **OR** supplier name, catalog number, clone number, **OR** RRID.	Not Applicable	
**Animal observed in or captured from the field:** Provide species, sex, and age where possible.	Not Applicable	
Please detail **housing and husbandry conditions**.	Not Applicable	

Plants and microbes	Information included in the manuscript?	In which section is the information available? (Reagents and Tools Table, Materials and Methods, Figures, Data Availability Section)
**Plants:** provide species and strain, ecotype and cultivar where relevant, unique accession number if available, and source (including location for collected wild specimens).	Not Applicable	
**Microbes:** provide species and strain, unique accession number if available, and source.	Not Applicable	

Human research participants	Information included in the manuscript?	In which section is the information available? (Reagents and Tools Table, Materials and Methods, Figures, Data Availability Section)
If collected and within the bounds of privacy constraints report on age, sex and gender or ethnicity for all study participants.	Not Applicable	

Core facilities	Information included in the manuscript?	In which section is the information available? (Reagents and Tools Table, Materials and Methods, Figures, Data Availability Section)
If your work benefited from core facilities, was their service mentioned in the acknowledgments section?	Yes	Acknowledgements

Design

Study protocol	Information included in the manuscript?	In which section is the information available? (Reagents and Tools Table, Materials and Methods, Figures, Data Availability Section)
If study protocol has been **pre-registered, provide DOI in the manuscript**. For clinical trials, provide the trial registration number **OR** cite DOI.	Not Applicable	
Report the **clinical trial registration number** (at ClinicalTrials.gov or equivalent), where applicable.	Not Applicable	

Laboratory protocol	Information included in the manuscript?	In which section is the information available? (Reagents and Tools Table, Materials and Methods, Figures, Data Availability Section)
Provide DOI OR other citation details if **external detailed step-by-step protocols** are available.	Not Applicable	

Experimental study design and statistics	Information included in the manuscript?	In which section is the information available? (Reagents and Tools Table, Materials and Methods, Figures, Data Availability Section)
Include a statement about **sample size** estimate even if no statistical methods were used.	Not Applicable	
Were any steps taken to minimize the effects of subjective bias when allocating animals/samples to treatment (e.g. **randomization procedure**)? If yes, have they been described?	Not Applicable	
Include a statement about **blinding** even if no blinding was done.	Not Applicable	
Describe **inclusion/exclusion criteria** if samples or animals were excluded from the analysis. Were the criteria pre-established? If sample or data points were omitted from analysis, report if this was due to attrition or intentional exclusion and provide justification.	Not Applicable	
For every figure, are **statistical tests** justified as appropriate? Do the data meet the assumptions of the tests (e.g., normal distribution)? Describe any methods used to assess it. Is there an estimate of variation within each group of data? Is the variance similar between the groups that are being statistically compared?	Yes	F-test using a P-value of 0.05 performed to determine if Homoscedastic o

Sample definition and in-laboratory replication	Information included in the manuscript?	In which section is the information available? (Reagents and Tools Table, Materials and Methods, Figures, Data Availability Section)
In the figure legends: state number of times the experiment was **replicated** in laboratory.	Yes	Figure Legends
In the figure legends: define whether data describe **technical or biological replicates**.	Yes	Figure Legends

Ethics

Ethics	Information included in the manuscript?	In which section is the information available? (Reagents and Tools Table, Materials and Methods, Figures, Data Availability Section)
Studies involving **human participants**: State details of **authority granting ethics approval** (IRB or equivalent committee(s), provide reference number for approval.	Not Applicable	
Studies involving **human participants**: Include a statement confirming that **informed consent** was obtained from all subjects and that the experiments conformed to the principles set out in the WMA Declaration of Helsinki and the Department of Health and Human Services Belmont Report.	Not Applicable	
Studies involving **human participants:** For publication of **patient photos**, include a statement confirming that consent to publish was obtained.	Not Applicable	
Studies involving experimental **animals**: State details of **authority granting ethics approva**l (IRB or equivalent committee(s), provide reference number for approval. Include a statement of compliance with ethical regulations.	Not Applicable	
Studies involving **specimen and field samples:** State if relevant **permits** obtained, provide details of authority approving study; if none were required, explain why.	Not Applicable	

Dual Use Research of Concern (DURC)	Information included in the manuscript?	In which section is the information available? (Reagents and Tools Table, Materials and Methods, Figures, Data Availability Section)
Could your study fall under dual use research restrictions? Please check biosecurity documents and list of **select agents and toxins** (CDC): https://www.selectagents.gov/sat/list.htm	Not Applicable	
If you used a select agent, is the security level of the lab appropriate and reported in the manuscript?	Not Applicable	
If a study is subject to dual use research of concern regulations, is the name of the **authority granting approval and reference number** for the regulatory approval provided in the manuscript?	Not Applicable	

Reporting

The MDAR framework recommends adoption of discipline-specific guidelines, established and endorsed through community initiatives. Journals have their own policy about requiring specific guidelines and recommendations to complement MDAR.

Adherence to community standards	Information included in the manuscript?	In which section is the information available? (Reagents and Tools Table, Materials and Methods, Figures, Data Availability Section)
State if relevant guidelines or checklists (e.g., **ICMJE, MIBBI, ARRIVE, PRISMA**) have been followed or provided.	Not Applicable	
For **tumor marker prognostic studies,** we recommend that you follow the **REMARK** reporting guidelines (see link list at top right). See author guidelines, under 'Reporting Guidelines'. Please confirm you have followed these guidelines.	Not Applicable	
For **phase II and III randomized controlled trials**, please refer to the **CONSORT** flow diagram (see link list at top right) and submit the CONSORT checklist (see link list at top right) with your submission. See author guidelines, under 'Reporting Guidelines'. Please confirm you have submitted this list.	Not Applicable	

Data Availability

Data availability	Information included in the manuscript?	In which section is the information available? (Reagents and Tools Table, Materials and Methods, Figures, Data Availability Section)
Have **primary datasets** been deposited according to the journal's guidelines (see 'Data Deposition' section) and the respective accession numbers provided in the Data Availability Section?	Not Applicable	Data availability
Were **human clinical and genomic datasets** deposited in a public access-controlled repository in accordance to ethical obligations to the patients and to the applicable consent agreement?	Not Applicable	
Are **computational models** that are central and integral to a study available without restrictions in a machine-readable form? Were the relevant accession numbers or links provided?	Yes	Material and Methods, Data availability
If publicly available data were reused, provide the respective **data citations in the reference list**.	Yes	Reference List
